# Supplementary material for: Effects of neuromuscular training on knee proprioception in individuals with anterior cruciate ligament injury: a systematic review and GRADE evidence synthesis
Source: BMJ Open. 2021 May 18;11(5):e049226. doi: 10.1136/bmjopen-2021-049226 (PMC8130739; doi:10.1136/bmjopen-2021-049226)
Supplement: Supplementary data [file bmjopen-2021-049226supp001.pdf]

Online supplemental file 1.

Database-specific search strategies

AMED

(Propriocep* OR (ZU "proprioception") OR Kinesthe* OR (ZU "kinesthesis") OR sensorimotor OR sensory-motor OR "joint position sense" OR "joint position detection" OR "threshold to detect passive motion" OR "passive motion direction discrimination" OR "passive motion detection threshold" OR "threshold for motion detection" OR "threshold hunting" OR "detection threshold" OR "discrimination threshold" OR "ipsilateral matching" OR "contralateral matching" OR "joint angle error" OR "distance estimation error" OR "passive recognition" OR "direction accuracy" OR "active reproduction" OR "joint reposition" OR "force sense" OR "force perception" OR "velocity sense" OR "active movement extent discrimination") AND (S1 AND S2 AND S3 AND S4)
S1: “Anterior Cruciate Ligament” OR (ZU "anterior cruciate ligament") OR “Knee joint” OR (ZU "knee joint)
S2: Injur* OR (ZU "injuries") OR (ZU "anterior cruciate ligament injuries") OR Reconstruction OR (ZU "anterior cruciate ligament reconstruction") OR
S3: Propriocep* OR (ZU "proprioception") OR Neuromuscular OR sensorimotor OR sensory-motor OR "Kinetic chain" OR (ZU "kinetics") OR Coordination OR Balance OR (ZU "balance") OR Plyometric (ZU "plyometric exercise") OR Vibration OR (ZU "vibration") OR Exercise* OR (ZU "exercise") OR Intervention OR Training OR Rehabilitation OR (ZU "rehabilitation") OR Therap* OR (ZU "therapy") OR Treatment
S4: Propriocep* OR (ZU "proprioception") OR Kinesthe* OR (ZU "kinesthesis") OR sensorimotor OR sensory-motor OR "joint position sense" OR "joint position detection" OR "threshold to detect passive motion" OR "passive motion direction discrimination" OR "passive motion detection threshold" OR "threshold for motion detection" OR "threshold hunting" OR "detection threshold" OR "discrimination threshold" OR "ipsilateral matching" OR "contralateral matching" OR "joint angle error" OR "distance estimation error" OR "passive recognition" OR "direction accuracy" OR "active reproduction" OR "Joint reposition" OR "force sense" OR "force perception" OR "velocity sense" OR "active movement extent discrimination"
Limiters - Language: English, Expanders - Apply related words, Search modes - Find any of my search terms, Interface - EBSCOhost Research Databases, Search Screen - Advanced Search, Database - AMED - The Allied and Complementary Medicine Database

CINAHL

Limiters - Peer Reviewed; Human; Language: English, Expanders - Apply related words, Search modes - Find any of my search terms, Interface - EBSCOhost Research Databases, Search Screen - Advanced Search, Database - CINAHL with Full Text
(Propriocep* OR (MH "Proprioception+") OR Kinesthe* OR (MH "Kinesthesis") OR sensorimotor OR sensory-motor OR "joint position sense" OR "joint position detection" OR "threshold to detect passive motion" OR "passive motion direction discrimination" OR "passive motion detection threshold" OR "threshold for motion detection" OR "threshold hunting" OR "detection threshold" OR "discrimination threshold" OR "ipsilateral matching" OR "contralateral matching" OR "joint angle error" OR "distance estimation error" OR "passive recognition" OR

"direction accuracy" OR "active reproduction" OR "Joint reposition" OR "Active movement extent discrimination" OR "force sense" OR "force perception" OR "velocity sense") AND (S6 AND S7 AND S8 AND S9)
S6: “Anterior Cruciate Ligament” OR (MH "Anterior Cruciate Ligament") “Knee joint” OR (MH "Knee Joint+"
S7: Injur* OR (MH "Anterior Cruciate Ligament Injuries") OR Reconstruction OR (MH "Anterior Cruciate Ligament Reconstruction") OR Rupture OR Tear OR (MH "Rupture+") OR Conservative OR Deficiency OR “Joint instability” OR (MH "Joint Instability+"
S8: Propriocep* OR (MH "Proprioception+") OR Neuromuscular OR (MH "Neuromuscular Control") OR sensorimotor OR "sensory-motor" OR "Kinetic chain" OR (MH "Closed Kinetic Chain Exercises") OR (MH "Open Kinetic Chain Exercises") OR Coordination OR Balance OR (MH "Balance Training, Physical") OR (MH "Balance, Postural") OR Plyometric OR Vibration OR (MH "Vibration" OR Exercise* OR (MH "Exercise+") OR Intervention OR Training OR Rehabilitation OR Therapy OR (MH "Physical Therapy+") OR Treatment
S9: Propriocep* OR (MH "Proprioception+") OR Kinesthe* OR (MH "Kinesthesis") OR sensorimotor OR sensory-motor OR "joint position sense" OR "joint position detection" OR "threshold to detect passive motion" OR "passive motion direction discrimination" OR "passive motion detection threshold" OR "threshold for motion detection" OR "threshold hunting" OR "detection threshold" OR "discrimination threshold" OR "ipsilateral matching" OR "contralateral matching" OR "joint angle error" OR "distance estimation error" OR "passive recognition" OR "direction accuracy" OR "active reproduction" OR "Joint reposition" OR "force sense" OR "force perception" OR "velocity sense" OR "Active movement extent discrimination"

Physical Education Index (ProQuest)

((("Anterior Cruciate Ligament" OR "Knee joint") AND (Injur* OR Trauma OR Reconstruct* OR Ruptur* OR Tear OR Conservative OR Deficienc* OR "Joint instabilit*") AND (Propriocep* OR Kinesthes* OR neuromuscular OR sensorimotor OR sensory-motor OR "Kinetic chain" OR Coordination OR Balance OR Plyometric OR Vibration OR Exercise* OR Intervention OR Training OR Rehabilitation OR Therap* OR Treatment) AND (Propriocep* OR Kinesthes* OR sensorimotor OR sensory-motor OR "joint position sense" OR "joint position detection" OR "threshold to detect passive motion" OR "passive motion direction discrimination" OR "passive motion detection threshold" OR "threshold for motion detection" OR "threshold hunting" OR "detection threshold" OR "discrimination threshold" OR "ipsilateral matching" OR "contralateral matching" OR "joint angle error" OR "distance estimation error" OR "passive recognition" OR "direction accuracy" OR "active reproduction" OR "Joint reposition" OR "active movement extent discrimination" OR "force sense" OR "force perception" OR "velocity sense"))) AND at.exact("Article") AND la.exact("ENG") AND PEER(yes)

PubMed

(((((Anterior Cruciate Ligament[Text Word] OR "Anterior Cruciate Ligament"[Mesh] OR Knee joint[Text Word] OR "knee joint"[MeSH Terms]) AND "loattrfull text"[sb]) AND (((injury[All Fields]) OR Reconstruction[Text Word] OR "Anterior Cruciate Ligament Reconstruction"[Mesh] OR "Anterior Cruciate Ligament Injuries"[Mesh] OR Rupture[Text Word] OR Tear[Text Word] OR "Rupture"[Mesh] OR Conservative[Text Word] OR

"Conservative Treatment"[Mesh] OR Deficiency[Text Word] OR Joint instability[Text Word] OR "Joint Instability"[Mesh]))) AND (((proprioception[All Fields]) OR "Proprioception"[Mesh] OR Neuromuscular[Text Word] OR sensorimotor[Text Word] OR sensory-motor[Text Word] OR Kinetic chain[Text Word] OR Coordination[Text Word] OR "Psychomotor Performance"[Mesh] OR Balance[Text Word] OR "Postural Balance"[Mesh] OR Plyometric[Text Word] OR "Plyometric Exercise"[Mesh] OR ("exercise"[MeSH Terms] OR "exercises"[All Fields] OR "exercise therapy"[MeSH Terms]) OR "Exercise Therapy"[Mesh] OR Intervention[Text Word] OR Training[Text Word] OR "Resistance Training"[Mesh] OR Rehabilitation[Text Word] OR "Rehabilitation"[Mesh] OR Therapy[Text Word] OR Treatment[Text Word] OR "Treatment Outcome"[Mesh]))) AND (((proprioception[All Fields]) OR "Proprioception"[Mesh] OR ("kinesthesis"[MeSH Terms] OR "kinesthesis"[All Fields]) OR "Kinesthesis"[Mesh] OR joint position sense[Text Word] OR (("joints"[MeSH Terms] OR "joints"[All Fields] OR "joint"[All Fields]) AND position detection[Text Word]) OR threshold to detect passive motion[Text Word] OR (passive[All Fields] AND motion direction discrimination[Text Word]) OR (passive[All Fields] AND motion detection threshold[Text Word]) OR (threshold[All Fields] AND motion detection[Text Word]) OR threshold hunting[Text Word] OR detection threshold[Text Word] OR discrimination threshold[Text Word] OR (ipsilateral[All Fields] AND matching[Text Word]) OR contralateral matching[Text Word] OR joint angle error[Text Word] OR distance estimation error[Text Word] OR passive recognition[Text Word] OR direction accuracy[Text Word] OR active reproduction[Text Word] OR Joint reposition[Text Word] OR force sense[Text Word] OR force perception[Text Word] OR velocity sense[Text Word] OR (active[All Fields] AND ("movement"[MeSH Terms] OR "movement"[All Fields]) AND extent[All Fields] AND ("discrimination (psychology)"[MeSH Terms] OR ("discrimination"[All Fields] AND "(psychology)"[All Fields]) OR "discrimination (psychology)"[All Fields] OR "discrimination"[All Fields])) OR sensorimotor[Text Word] OR sensory-motor[Text Word]) AND "loattrfull text"[sb])) AND "loattrfull text"[sb] AND ("loattrfull text"[sb] AND English[lang]) AND English[lang]

Scopus

("Anterior Cruciate Ligament" OR "Knee joint") AND (injur* OR trauma OR reconstruct* OR ruptur* OR tear OR conservative OR deficienc* OR "Joint instabilit*") AND (propriocep* OR kinesthes* OR neuromuscular OR sensorimotor OR sensory-motor OR "Kinetic chain" OR coordination OR balance OR plyometric OR vibration OR exercise* OR intervention OR training OR rehabilitation OR therap* OR treatment) AND (propriocep* OR kinesthes* OR "joint position sense" OR "joint position detection" OR "threshold to detect passive motion" OR "passive motion direction discrimination" OR "passive motion detection threshold" OR "threshold for motion detection" OR "threshold hunting" OR "detection threshold" OR "discrimination threshold" OR "ipsilateral matching" OR "contralateral matching" OR "joint angle error" OR "distance estimation error" OR "passive recognition" OR "direction accuracy" OR "active reproduction" OR "Joint reposition" OR "active movement extent discrimination" OR "force sense" OR "force perception" OR "velocity sense" OR sensorimotor OR sensory-motor) AND NOT INDEX (medline) AND (LIMIT-TO (SRCTYPE , "j")) AND (LIMIT-TO (DOCTYPE , "ar")) AND (LIMIT-TO (SUBJAREA , "MEDI") OR LIMIT-TO (SUBJAREA , "HEAL") OR LIMIT-TO (SUBJAREA , "NEUR")) AND (LIMIT-TO (LANGUAGE , "English")) AND (LIMIT-TO (EXACTKEYWORD , "Human") OR LIMIT-TO (EXACTKEYWORD ,

"Article") OR LIMIT-TO (EXACTKEYWORD , "Male") OR LIMIT-TO (EXACTKEYWORD , "Female") OR LIMIT-TO (EXACTKEYWORD , "Controlled Study") OR LIMIT-TO (EXACTKEYWORD , "Proprioception"))

SPORTDiscus

Limiters - Peer Reviewed; Language: English; Publication Type: Academic Journal; Document Type: Article, Expanders - Apply related words, Search modes - Find any of my search terms, Interface - EBSCOhost Research Databases, Search Screen - Advanced Search, Database - SPORTDiscus
(Propriocep* OR (DE "PROPRIOCEPTION") OR Kinesthe* OR sensorimotor OR sensory-motor OR "joint position sense" OR "joint position detection" OR "threshold to detect passive motion" OR "passive motion direction discrimination" OR "passive motion detection threshold" OR "threshold for motion detection" OR "threshold hunting" OR "detection threshold" OR "discrimination threshold" OR "ipsilateral matching" OR "contralateral matching" OR "joint angle error" OR "distance estimation error" OR "passive recognition" OR "direction accuracy" OR "active reproduction" OR "Joint reposition" OR "force sense" OR "force perception" OR "velocity sense" OR "active movement extent discrimination") AND (S1 AND S2 AND S3 AND S4)
S1: Anterior Cruciate Ligament OR (DE "CRUCIATE ligaments") OR (DE "ANTERIOR cruciate ligament") “Knee joint” OR (DE "KNEE"
S2: Injur* OR (DE "ANTERIOR cruciate ligament injuries") OR (DE "CRUCIATE ligament injuries) OR Reconstruction OR Rupture OR Tear OR Conservative OR Deficiency OR “Joint instabilit*”
S3: Propriocep* OR (DE "PROPRIOCEPTION") OR Neuromuscular OR sensorimotor OR sensory-motor OR Kinetic chain OR Coordination OR (DE "MOTOR ability") OR Balance OR Plyometric OR (DE "PLYOMETRICS) OR Vibration OR Exercise* OR Intervention OR Training OR Rehabilitation OR (DE "TREATMENT programs") OR (DE "REHABILITATION") OR Therap* OR Treatment OR (DE "KNEE injuries -- Treatment")
S4: Propriocep* OR (DE "PROPRIOCEPTION") OR Kinesthe* OR sensorimotor OR sensory-motor OR "joint position sense" OR "joint position detection" OR "threshold to detect passive motion" OR "passive motion direction discrimination" OR "passive motion detection threshold" OR "threshold for motion detection" OR "threshold hunting" OR "detection threshold" OR "discrimination threshold" OR "ipsilateral matching" OR "contralateral matching" OR "joint angle error" OR "distance estimation error" OR "passive recognition" OR "direction accuracy" OR "active reproduction" OR "Joint reposition" OR "force sense" OR "force perception" OR "velocity sense" OR "active movement extent discrimination"

Online supplemental file 2.

Screening protocol – to screen eligible studies at the title, abstract, and full-text screening stages

Questions for all stages: title, abstract and full-text screening (follow stages 1-9):

1) Is the study published in a scientific journal or published as a dissertation/thesis?
    a. No - exclude
    b. Yes or uncertain - go to step 2
2) Is the study written in English?
    a. No - exclude
    b. Yes or uncertain - go to step 3
3) Does the study deal with individuals who are 15 years of age and above?
    a. No - exclude
    b. Yes or uncertain - go to step 4
4) Does this study investigate individuals with an anterior cruciate ligament injury managed with conservative treatment or surgical reconstruction?
    a. No - exclude
    b. Yes or uncertain - go to step 5
5) Is the study a primary study (i.e. no letter to the editor, book reviews, published study designs/trial protocols, commentaries, editorials, interviews, newspaper articles, patient education handouts, consensus statements or clinical practice guidelines)?
    a. No - exclude
    b. Yes or uncertain - go to step 6
6) Does the intervention group in the study undergo neuromuscular training rehabilitation?
    a. No - exclude
    b. Yes or uncertain - go to step 7
7) Is the comparator/control group in the study include any of the following: any other therapy, conventional training, usual care, placebo or sham therapy?
    a. No - exclude
    b. Yes or uncertain - go to step 8
8) Does the study evaluate knee proprioception using a specific test (joint position sense, joint position detection, threshold to detect passive motion, passive motion direction discrimination, passive motion detection threshold, threshold for motion detection, threshold hunting, detection threshold, discrimination threshold, ipsilateral matching, contralateral matching, joint angle error, distance estimation error, passive recognition, direction accuracy, active reproduction, active movement extent discrimination, force sense, force perception, velocity sense or any other related tests)- before and after the intervention?
    a. No - exclude
    b. Yes or uncertain - go to step 9
9) Does the study report (objective) focal measures of knee proprioception for any of the specific tests mentioned in point 8?
    a. No - exclude
    b. Yes or uncertain - choose one of the following options:

i. Title and abstract screening stage - include
ii. Full-text screening stage - follow step 10-11

Additional questions for full-text stage only:

10) Does the study use at least one (appropriate) statistical test to compare the intervention and comparator/control groups for knee proprioception?
    a. No - exclude
    b. Yes or uncertain - go to step 11
11) Are the points 1-10 scored as "yes or uncertain"
    a. If all "yes" - include
    b. If any "uncertain" - discuss with another reviewer to come to an agreement whether to include the study or not

Online supplemental file 3.
Data extraction template

Publication details	Study citation, clinical trial registration, and published study protocol if available
Aim of the study	Primary and/or secondary aims relevant for the review.
Eligibility criteria	Inclusion and exclusion criteria for participants
Randomized controlled trial or controlled clinical trial	Randomization method?
Participant allocation	Concealed or not?
Number of participants identified	Identified, included and excluded?
All participants accounted for entire study	Yes or no?
Experimental group	Experimental intervention (type of neuromuscular rehabilitation training) given.
Comparator group	Comparator intervention given.
Assessment method, equipment used, and outcome measure(s) of interest	Those related to knee-specific proprioception senses.
Method(s) used for measuring the outcome(s) appropriate?	Authors quoted any data on reliability and validity based on the previous literature or their own data?
Multiple measurements of the same outcome measure within the outcome domain?	Different methods measuring same proprioception sense and different time points?
Participant characteristics	Anthropometric, demographic, physical activity and function levels, and any other relevant information to ACL injury and/or surgery.
Groups were similar at baseline	Anthropometrics, demographics, outcome measure(s) of interest, and any other prognostic indicators.
Blinding	Participants, investigators, therapists/clinicians/those delivering the interventions, and outcome assessors.
The outcome measure of interest was obtained from more than 85% of the participants initially allocated to groups	For continuous outcomes, availability of data from 95% (or possibly 90%) of the participants would often be sufficient.
If data were missing, how they were handled	‘Last observation carried forward’, ‘baseline observation carried forward’ or any other method?
Analyses preplanned	Information available from Registered trial protocol or any other relevant information available?
Between-group statistical comparisons	Statistical analysis for measurement of proprioception was done by “intention to treat” or “per-protocol” analysis? Multiple analysis of data? Corrected for multiple analysis of data? Selective reporting of analysis?
Results	Selective reporting of a particular outcome measurement?
Conclusion	Authors’ conclusions
