# Supplementary material for: Effects of neuromuscular training on knee proprioception in individuals with anterior cruciate ligament injury: a systematic review and GRADE evidence synthesis
Source: BMJ Open. 2021 May 18;11(5):e049226. doi: 10.1136/bmjopen-2021-049226 (PMC8130739; doi:10.1136/bmjopen-2021-049226)
Supplement: Supplementary data [file bmjopen-2021-049226supp002.pdf]

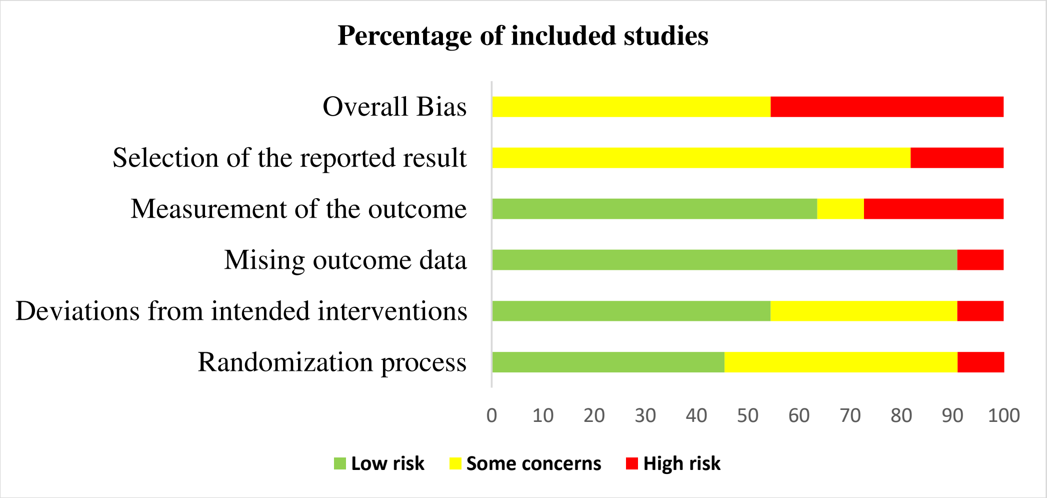

Online supplemental figure 1. Risk of bias assessment in each of the five domains and overall bias. Percentage of studies showing low risk of bias, some concerns and high risk of bias.

Note: For studies having more than one relevant outcome, each outcome is considered separately for risk of bias assessment.
