# Supplementary material for: Effects of neuromuscular training on knee proprioception in individuals with anterior cruciate ligament injury: a systematic review and GRADE evidence synthesis
Source: BMJ Open. 2021 May 18;11(5):e049226. doi: 10.1136/bmjopen-2021-049226 (PMC8130739; doi:10.1136/bmjopen-2021-049226)
Supplement: Author's manuscript [file bmjopen-2021-049226.draft_revisions.pdf]

BMJ Open

BMJ Open is committed to open peer review. As part of this commitment we make the peer review history of every article we publish publicly available.

When an article is published we post the peer reviewers' comments and the authors' responses online. We also post the versions of the paper that were used during peer review. These are the versions that the peer review comments apply to.

The versions of the paper that follow are the versions that were submitted during the peer review process. They are not the versions of record or the final published versions. They should not be cited or distributed as the published version of this manuscript.

BMJ Open is an open access journal and the full, final, typeset and author-corrected version of record of the manuscript is available on our site with no access controls, subscription charges or pay-per-view fees (http://bmjopen.bmj.com).

If you have any questions on BMJ Open's open peer review process please email info.bmjopen@bmj.com

BMJ Open

# Effects of neuromuscular training on knee proprioception in individuals with anterior cruciate ligament injury - A systematic review and GRADE evidence synthesis

| | |
|---|---|
| Journal: | *BMJ Open* |
| Manuscript ID | bmjopen-2021-049226 |
| Article Type: | Original research |
| Date Submitted by the Author: | 19-Jan-2021 |
| Complete List of Authors: | Arumugam, Ashokan; College of Health Sciences University of Sharjah, Department of Physiotherapy<br>Björklund, Martin; Umeå University, Department of Community Medicine and Rehabilitation – Physiotherapy Section; Centre for Musculoskeletal Research University of Gävle, Department of Occupational Health Sciences and Psychology, Centre for Musculoskeletal Research<br>Mikko, Sanna; Umeå University, Department of Community Medicine and Rehabilitation – Physiotherapy Section<br>Häger, Charlotte ; Umeå University, 1Department of Community Medicine and Rehabilitation – Physiotherapy Section |
| Keywords: | Knee < ORTHOPAEDIC & TRAUMA SURGERY, Orthopaedic sports trauma < ORTHOPAEDIC & TRAUMA SURGERY, REHABILITATION MEDICINE, SPORTS MEDICINE |
| | |

SCHOLARONE™
Manuscripts

BMJ

*I, the Submitting Author has the right to grant and does grant on behalf of all authors of the Work (as defined in the below author licence), an exclusive licence and/or a non-exclusive licence for contributions from authors who are: i) UK Crown employees; ii) where BMJ has agreed a CC-BY licence shall apply, and/or iii) in accordance with the terms applicable for US Federal Government officers or employees acting as part of their official duties; on a worldwide, perpetual, irrevocable, royalty-free basis to BMJ Publishing Group Ltd ("BMJ") its licensees and where the relevant Journal is co-owned by BMJ to the co-owners of the Journal, to publish the Work in this journal and any other BMJ products and to exploit all rights, as set out in our licence.*

*The Submitting Author accepts and understands that any supply made under these terms is made by BMJ to the Submitting Author unless you are acting as an employee on behalf of your employer or a postgraduate student of an affiliated institution which is paying any applicable article publishing charge ("APC") for Open Access articles. Where the Submitting Author wishes to make the Work available on an Open Access basis (and intends to pay the relevant APC), the terms of reuse of such Open Access shall be governed by a Creative Commons licence – details of these licences and which Creative Commons licence will apply to this Work are set out in our licence referred to above.*

*Other than as permitted in any relevant BMJ Author's Self Archiving Policies, I confirm this Work has not been accepted for publication elsewhere, is not being considered for publication elsewhere and does not duplicate material already published. I confirm all authors consent to publication of this Work and authorise the granting of this licence.*

For peer review only

**Effects of neuromuscular training on knee proprioception in individuals with anterior cruciate ligament injury - A systematic review and GRADE evidence synthesis**

**Authors:** Ashokan Arumugam (M.P.T., Ph.D.)[1], Martin Björklund (R.P.T., Ph.D.)[2, 3], Sanna Mikko (B.Sc.)[2], Charlotte K. Häger (R.P.T., Ph.D.)[2]

**Author Affiliations:**

[1]Department of Physiotherapy, College of Health Sciences, University of Sharjah, P.O. Box: 27272, Sharjah, United Arab Emirates;
[2]Department of Community Medicine and Rehabilitation – Physiotherapy Section, Umeå, University, SE-901 87, Umeå, Sweden;
[3]Department of Occupational Health Sciences and Psychology, Centre for Musculoskeletal Research, University of Gävle, Gävle, Sweden.

**Corresponding author:** Charlotte K. Häger

**Address for Correspondence:**

| | |
|---|---|
| Name | Charlotte K. Häger |
| Designation | Professor |
| Department | Community Medicine & Rehabilitation – Physiotherapy Section |
| Institution | Umeå, University |
| Country | Sweden |
| Phone | +971 503193843 |
| Email | charlotte.hager@umu.se |


**Manuscript Type:** A systematic review

**Word count:** 4517 (excluding abstract, tables, contributions, funding acknowledgements and references)

**Ethical approval:** Not required.

**Funding sources:** The Swedish Research Council (2017-00892); Region Västerbotten (ALF 7003575; Strategic funding VLL-358901; Project.No 7002795); The Swedish Research Council for Sports Science (CIF P2019 0068); and King Gustaf V and Queen Victoria's Foundation of Freemasons 2019 (Häger).

**Conflicts of interest:** None declared.

**Ethical approval:** Not required.

**Competing interests:** None declared.



For peer review only - http://bmjopen.bmj.com/site/about/guidelines.xhtml

# Effects of neuromuscular training on knee proprioception in individuals with anterior cruciate ligament injury - A systematic review and GRADE evidence synthesis


## Abstract

**Objective**

To systematically review and summarize the evidence for the effects of neuromuscular training on knee proprioception following ACL injury.

**Design**

Systematic Review

**Methods**

PubMed, CINAHL, SPORTDiscus, AMED, Scopus, and Physical Education Index were searched from inception to February 2020. Controlled or randomized clinical trials (RCTs) investigating the effects of neuromuscular training on knee-specific proprioception tests in individuals with a unilateral ACL injury were included. Two reviewers independently screened and extracted data and assessed risk of bias of the eligible RCTs using the Cochrane risk of bias 2 tool. Overall certainty in evidence was determined using the GRADE tool.

**Results**

Of 2706 articles retrieved, only nine RCTs, comprising in total 327 individuals with an ACL reconstruction (ACLR), met the inclusion criteria. Neuromuscular training interventions varied across studies: whole body vibration therapy, Nintendo-Wii-Fit training, balance training, sport-specific exercises, backward walking, etc. Outcome measures included joint position sense (JPS; n=7), thresholds to detect passive motion (TTDPM; n=3), or quadriceps force control (QFC; n=1). Overall, there were conflicting findings for reduced errors associated with JPS (one or more target angles), TTDPM or QFC of ACLR knee following neuromuscular training. Owing to


For peer review only



For peer review only - http://bmjopen.bmj.com/site/about/guidelines.xhtml

serious concerns with three or more GRADE domains (risk of bias, inconsistency, indirectness, or imprecision associated with the findings) for each outcome of interest across studies, the certainty of evidence was very low.

**Conclusions**

The heterogeneity of interventions, methodological limitations, inconsistency of effects (on JPS/TTDPM/QFC) preclude recommendation of one optimal neuromuscular training intervention for improving proprioception following ACL injury in clinical practice. The low evidence thus questions common clinical neuromuscular training programs in practice. Our review highlights the urgent need for methodologically-robust RCTs with homogenous populations with ACL injury (managed conservatively or with reconstruction), novel/well-designed neuromuscular training interventions, and valid proprioception assessments, which also seem to be lacking.

**PROSPERO registration number**

CRD42018107349

**Key words:** Joint position sense, threshold to detect passive motion, ACL, sensorimotor training, literature review, neuroplasticity

For peer review only



For peer review only - http://bmjopen.bmj.com/site/about/guidelines.xhtml

41 **Strengths and limitations of the study**

42 - A systematic review of neuromuscular training on knee proprioception following the Preferred Reporting Items for Systematic reviews and Meta-Analyses guidelines, using a broad search in six electronic databases.
45 - The risk of bias associated with the outcomes of interest (knee proprioception measures) in the included RCTs were assessed using the updated Cochrane risk of bias 2 tool.
47 - The overall certainty of evidence for the effects of neuromuscular training on knee joint position sense, threshold to detect passive motion, and quadriceps force control following ACL injury/reconstruction was ascertained using the GRADE tool.
50 - Only RCTs published in English were included.
51 - A meta-analysis was precluded because of clinical heterogeneity of interventions and outcome measures.

53



For peer review only - http://bmjopen.bmj.com/site/about/guidelines.xhtml

For peer review only

## Introduction

Anterior cruciate ligament (ACL) injury is a common musculoskeletal injury[1, 2] accounting for an annual incidence rate of 68.6/100,000 person-years in the United States.[3] ACL injury is most prevalent in young athletes.[3] The injury occurs more often during competition rather than training, with ~70% or more of the injuries representing noncontact mechanisms[4, 5] such as landing from a jump, sudden deceleration and/or while cutting.[6] Thus, the injury mechanisms are related to neuromotor control, amongst other factors, of the individual. ACL injury is followed by a long period of rehabilitation and yet many individuals do not return to pre-injury levels of activity[7] which challenges the efficacy of existing preventative and rehabilitative strategies.

Individuals with an ACL injury present with a decreased number of proprioceptive mechanoreceptors (Pacinian capsules, Ruffini nerve endings and Golgi tendon organs)[8, 9] in the knee which might alter the somatosensory input to the central nervous system (CNS)[9] leading to decreased knee proprioception. Disturbed proprioception might also be caused by acute inflammation and pain, and the capsule and surrounding ligaments getting affected following instability.[10, 11] Although there has been a debate regarding the effects of ACL injury on different knee proprioception tests,[2, 12] our recent systematic review[13] suggests that knee JPS tests have sufficient validity in discriminating ACL-injured knees from asymptomatic knees (under review following revision). When compared to non-injured controls, individuals with ACL injury demonstrate altered movement strategies,[4, 14] quadriceps muscle weakness,[15] and onset and progression of osteoarthrosis.[6, 16] Due to the potential serious consequences of the injury, much attention and clinical efforts have been dedicated to preventative and rehabilitative strategies for ACL injury[11], including various neuromuscular training (NT) methods believed to improve the proprioceptive ability.



For peer review only - http://bmjopen.bmj.com/site/about/guidelines.xhtml

Even if proprioceptive deficits may negatively affect the neuromotor control, the rationale, mechanisms, and plausibility for improving proprioception by training need to be verified. In the context of neuroplasticity, functional magnetic resonance imaging (fMRI) has revealed that individuals with ACL-deficient knees demonstrate less activation in several sensorimotor cortical areas and increased activation in pre-supplementary motor areas, posterior secondary somatosensory area, and posterior inferior temporal gyrus compared to controls with asymptomatic knees during a knee flexion-extension task.[1] It seems individuals with ACLR adapt a visual-sensory-motor strategy instead of a normal sensory-motor strategy owing to aberrant sensory feedback following ACL injury.[17] Nevertheless, neuroplastic reorganization ensues where other potential sensory sources are used to organize the movement or regulate neuromotor control, particularly in (sporting) tasks with higher complexity. Therefore, ACL injuries might be regarded as a neuromotor control dysfunction rather than a simple peripheral musculoskeletal injury.[11, 18] It remains unclear though whether NT can improve proprioception after an ACL injury[11, 19] and the neurophysiological mechanisms underpinning such interventions need further substantiation.

To date, there is no consensus on the most effective rehabilitation programs for ACL injury, and the prevalence of reinjury after returning to sport is ~30%.[18] Owing to the neuroplastic changes and possibly altered proprioception following an ACL injury, NT has received much attention to enhance dynamic joint stability and relearn movement patterns and skills.[20] In this context, both NT and sensorimotor training terms have been used in the literature to describe the same phenomenon. NT for e.g., is defined as "…training enhancing unconscious motor responses by stimulating both afferent signals and central mechanisms responsible for dynamic joint control" [20] and sensorimotor training has been described as aiming to improve "…function of the CNS in



For peer review only - http://bmjopen.bmj.com/site/about/guidelines.xhtml

regulating movement in order to reach proper firing patterns for maintaining joint stability…”.[21] Active knee motion will in any case stimulate proprioceptors, which in turn would alter the demands on the CNS.[10, 19] Henceforth we will use the term NT in this review.

There are different ways to challenge proprioception, for example: vibration may be used to alter afferent input from muscle spindles; an unstable surface can challenge input from the ankle; vision can be occluded or head position can be changed to disturb visual- and vestibular information,[10] or focus can be shifted to influence cognitive processing sources.[18] Due to a putative visual-sensory-motor strategy following ACL injury, a modified visual feedback training might decrease visual reliance and improve sensory-motor function.[18] Most studies exploring the effects of NT on proprioception combine different exercises and various outcome measures which precludes isolating the effects of a proprioception-specific exercise.[22] Therefore, this study aimed at systematically reviewing and summarizing the evidence for the effects of NT compared to comparator/control interventions on proprioception measured by knee-specific proprioception tests in individuals with ACL injury or reconstruction.

## Methods

We adhered to Preferred Reporting Items for Systematic review and Meta-Analysis (PRISMA) checklist.[23] The protocol was registered in PROSPERO (CRD42018107349).

### Eligibility criteria

The structure of PICOS[24] was used to frame the following criteria:

1. **Participants:** Individuals over 15 years of age (both sexes) with a history of a unilateral ACL rupture, managed conservatively or surgically reconstructed, with or without

For peer review only

concomitant meniscus and/or collateral ligament injuries, without any other lower extremity injuries/surgeries that would confound the outcomes of rehabilitation training;

2. **Intervention:** Specific NT, closed/open kinetic chain exercises, balance training, joint repositioning training, joint force sense training, co-ordination training, plyometric training, whole body vibration, virtual gaming training, an accelerated rehabilitation protocol or any other training programs focusing on improving the lower limb neuromuscular control and knee proprioception;
3. **Comparator:** Any other therapy, conventional training, usual care, placebo or sham therapy;
4. **Outcome measures:** Knee-specific proprioception tests targeting joint position sense (JPS), kinesthesia (threshold to detect passive motion [TTDPM]), force sense/perception, active movement extent discrimination, velocity sense, or psychophysical threshold methods;[13] they can be performed actively and/or passively with or without visual input in weight bearing or non-weight bearing positions;[10]
5. **Study design:** controlled or randomized clinical trials (RCTs).

**Data Sources and Searches**

Database-specific search terms (e.g. MeSH) were combined using Boolean operators ("AND" and "OR") under three conceptual domains: participants, interventions and outcomes. Six electronic databases were searched from their inception to 12 February 2020: PubMed, Cumulative Index to Nursing & Allied Health Literature (CINAHL), SPORTDiscus, the Allied and Complementary Medicine Database (AMED), Scopus, and Physical Education Index (via Proquest) (Online supplemental file 1).



For peer review only - http://bmjopen.bmj.com/site/about/guidelines.xhtml

**Study Selection**

One reviewer (SM) imported all titles and abstracts retrieved from the databases into EndNote X8. Two reviewers (AA and SM) independently checked titles, abstracts, and/or full text by following a screening questionnaire (online supplemental file 2). Any disagreements in inclusion of articles were adjudicated by two other reviewers (CH and MB) until consensus was reached. A manual search of the reference lists of included articles was performed.

**Data Extraction**

Data were extracted by one reviewer (SM) and verified by another reviewer (AA) using a customized data extraction sheet (online supplemental file 3). If any data were missing, the corresponding authors were contacted via email.

**Quality Assessment**

The risk of bias for each outcome of interest in the included studies was evaluated using the Cochrane ROB 2.[25] The tool has five domains: 1) randomization (number of signalling questions [n=3]), 2) deviations from intended interventions (n=7), 3) missing outcome data (n=5), 4) measurement of the outcomes (n=5), and 5) selection of the reported results (n=3). Each signalling question can be answered as 1) yes, 2) probably yes, 3) probably no, 4) no, and 5) no information. Responses to the questions provide the basis for judgement of the risk of bias at each domain level using a tool-specific algorithm resulting in one out of three possible judgements: 1) low risk of bias, 2) some concerns, or 3) high risk of bias. An overall risk of bias score for each outcome in a study can be low (with a low risk of bias for all domains), some concerns (if some concerns prevail in at least one domain without a high risk of bias for any

For peer review only



For peer review only - http://bmjopen.bmj.com/site/about/guidelines.xhtml

domain) or high (if a high risk of bias underpins at least one domain or some concerns remain in multiple domains, defining multiple as more than two).

**Evidence Synthesis**

We determined the overall evidence level in this review using the Grading of Recommendations, Assessment, Development and Evaluation (GRADE) recommendations considering risk of bias, inconsistency (heterogeneity) in results, indirectness of evidence, imprecision of results, and other domains (e.g. publication bias if applicable).[26] The overall evidence was rated as very low, low, moderate or high. A meta-analysis was precluded owing to clinical heterogeneity of interventions and outcome measurements.

**Patient and public involvement**

Neither patients nor public were involved.

**Results**

**Search Results**

Electronic databases search identified a total of 2706 articles (excluding duplicates: 2162). Following title and abstract screening, 22 articles were shortlisted for full-text screening and subsequently nine articles were found to meet the inclusion criteria (Figure 1). The other thirteen articles were excluded for the following reasons: not an RCT (n=1),[27] no knee-specific proprioception tests (n=6),[28-33] participants did not have an ACL injury (n=1),[34] knee proprioception data were missing and the corresponding author did not respond to our emails (n=1),[35] a comparison between different surgical intervention groups with the same identical



For peer review only - http://bmjopen.bmj.com/site/about/guidelines.xhtml

rehabilitation program (n=2),[36, 37] and finally, lack of a NT program (n=2).[38, 39] No additional relevant studies were identified through manual search of bibliographic references.

**Study Design and Participants**

All the nine studies included were RCTs with a total of 386 participants (only 327 were included in analysis), and two studies had their trial pre-registered.[40, 41] All participants had undergone a unilateral ACLR with either a bone-patellar-tendon-bone or a hamstring graft (Table 1).

**Quality Assessment**

The agreement (Cohen's kappa) of responses to the signalling questions between the two reviewers (AA and MB) was substantial ($0.69 \pm 0.047$, $p < 0.001$). Disagreements were discussed and resolved by the two reviewers. Online supplemental figure 1 shows the percentage of studies judged as low risk, some concerns and high risk of bias in the five domains, and Table 2 shows domain judgements of each study. The overall risk of bias judgement showed that four of the included studies had a high risk of bias,[42-45] four had some concerns,[41, 46-48] and one study[40] had a high risk of bias for JPS and some concerns for quadriceps force control (QFC). The domain that most consistently showed risk of bias across studies was bias in selection of the reported results (Online supplemental figure 1 and Table 2). The most common reason was the absence of information regarding pre-specified plan of analyses. None of the included studies reported trial protocol publication and only two[40, 41] reported trial registration. Furthermore, two studies were judged to perform inappropriate multiple analyses.[42, 43] Judgement of bias in measurement of the outcome (domain 4, Table 2) showed most scattered results across studies (Online supplemental figure 1). A high risk of bias was found in three studies of which one had no information on measurements[45] and two showed inappropriate measurement methods of the



For peer review only - http://bmjopen.bmj.com/site/about/guidelines.xhtml

outcome of interest.[40, 43] In the study by Zult et al., only one trial per target was performed to estimate JPS,[40] while Baltaci et al. used a test with presumably a high demand on motor and memory components,[43] without reporting its reliability or validity. The domain with least risk of bias was missing outcome data where all studies, except one,[44] had low risk of bias.

## Rehabilitation Programs

The studies included a spectrum of rehabilitation programs employed to influence knee proprioception (Table 1). Two studies[42, 47] explored the effects of whole-body vibration therapy (WBVT) combined with or without conventional rehabilitation compared to conventional rehabilitation alone. Cho et al. compared closed kinetic chain exercises on a balance pad versus on a stable floor.[45] Risberg et al. compared the effects of a NT compared to strength training. In their NT program, the first half of the rehabilitation focused on exercises on a wobble board or trampoline and exercises to increase the range of motion, while the end of the program focused on specific training of plyometric, agility and sport-specific skills.[46] Baltaci et al. investigated the effects of Nintendo-Wii-Fit compared to conventional rehabilitation.[43] Beynnon et al. evaluated the effects of accelerated (19 weeks) vs. non-accelerated (32 weeks) programs of conventional training.[48] The timeframe and exercises in their experimental program ranged from 1-7 weeks for range of motion and muscle activation, 8-11 weeks for dynamic functional activities such as biking and jogging, and finally, 12-19 weeks for plyometric and agility drill exercises.[48] Kaya et al. studied the effects of neuromuscular (motor control) exercises for the lower limbs combined with standard rehabilitation compared with standard rehabilitation alone.[44] Shen et al. examined the outcome of standard rehabilitation combined with backward walking at 1.3 km/h on a treadmill for four groups (at four inclination angles 0°, 5°, 10°, and 15°, respectively) compared to standard rehabilitation in a comparator group.[41] Nevertheless, Zult et



For peer review only
For peer review only - http://bmjopen.bmj.com/site/about/guidelines.xhtml

al. examined the effects of cross-education of strength training of the non-injured leg along with standard rehabilitation compared to standard rehabilitation alone.[40]

**Knee-specific Proprioceptive Measures**

Seven studies used active or passive JPS and all but one used (absolute) angular error as a variable to evaluate the outcome.[40-45, 47] Conversely, one study used monitored-rehab-system-software to define a virtual line/route to allow joint repositioning within 30-70% knee range of motion with and without visual feedback.[43] The differences between visual and blinded trails (2 each) based on the deviations from the computer-generated line (in mm) were used to measure proprioception.[43] All these studies used sitting or supine test position for assessing JPS. There were two to four predetermined target knee flexion angles across studies ranging from 15°-80°.[40-42, 44, 45, 47] Moreover, two studies[42, 43] used active knee motion and four used passive knee motion[40, 41, 44, 47] to set the target angle. Whether Cho et al. used active or passive knee motion to set/reproduce the target angle seems ambiguous. [45 45] Four studies[40, 42-45] used active knee motion and two[41, 47] used passive knee motion to reproduce the target angle. The JPS method used by Zult et al. was presumed based on their reference to Hortobagyi et al.[49]

The angular error was measured with 1-6 trials per each angle and one study[40] randomized the order of the joint angles used. Eyes were blinded during the test in six studies[40-42, 44, 45, 47] while one study used visual feedback when the individual was placing the knee in the target angle but no such feedback was given during reproduction of the target angle.[43] The difference between visual and non-visual trials was calculated in mm by the device as a measure of JPS.[43] A Biodex dynamometer (Biodex Medical Systems, Shirly, NY, USA) was used in five studies[40, 42, 44, 45, 47] to test JPS. Even so, one study used a continuous passive motion equipment[41] while another[43]



For peer review only - http://bmjopen.bmj.com/site/about/guidelines.xhtml

employed a functional squat system (Monitored Rehab System, Haarlem, and the Netherlands) with a leg press machine and an associated computer program for assessing JPS.

Three studies[41, 46, 48] evaluated knee kinaesthesia with TTDPM using a bespoke device,[46, 48] or a continuous passive motion equipment.[41] The knee was moved in flexion or extension at a constant angular velocity of 0.5°/s [46] or 0.1°/s.[41, 48] While the participants were blindfolded in two studies,[41, 48] the other study did not mention about visual feedback.[46] In all three studies, the tests were performed thrice in each direction (flexion and/or extension) for both legs but whether the order of direction or leg was randomized is not reported. In the study by Risberg et al.,[46] TTDPM data were missing for 27 out of 74 participants because of device failure, which might lower the power of the study.

**Assessing Certainty in Evidence**

There were serious concerns with three GRADE domains (risk of bias, indirectness, and imprecision associated with the findings) across the seven studies that measured JPS (Tables 3 and 4). The certainty of evidence found was very low for the effects of NT on improving JPS following ACLR.

There were further serious concerns with all GRADE domains (risk of bias, inconsistency, indirectness, and imprecision associated with the findings) across the three studies measuring TTDPM (Tables 3 and 4). Therefore, the certainty of evidence found was very low for improving TTDPM in individuals with ACLR following NT (Table 3).

An overall judgement of some concerns based on the Cochrane ROB 2 tool (Table 2) was found for the study reporting changes in QFC following NT.[40] Available population, the magnitude and direction of effect, and effect estimates of QFC (Tables 1 and 3) are derived from only one study



For peer review only - http://bmjopen.bmj.com/site/about/guidelines.xhtml

which reflect serious concerns. However, the participants with ACLR, intervention (cross-education of the quadriceps with standard rehabilitation), and QFC[40] are directly related to our research question. The certainty of evidence found was very low for improving QFC in individuals with ACLR following NT because only one relevant study was found.

## Discussion

This review is the first, as far as we are aware, to systematically review the level of evidence for effects of NT on knee proprioception in individuals with ACL injury. A previous review, however, summarized the effects of proprioceptive and balance exercises following ACL injury/reconstruction on certain outcome measures (muscle strength, hop test, etc.) but other than knee-specific proprioception tests.[50] Another similar review published in 2003 did not find any RCTs at all in this area.[51] As of today, we identified nine studies employing a range of NT methods, of which all but one[46] were published within the past decade. Nevertheless, there were serious concerns with two or more GRADE domains (risk of bias, inconsistency, indirectness, or imprecision associated with the findings) across studies implying a very low certainty of evidence for improving JPS, TTDPM, and QFC of ACLR knee following NT.

### *Effects of NT on Knee Proprioception in Individuals with ACLR*

Most of the employed NT programs did not influence proprioception compared to comparator interventions. Of the nine included articles, four studies reported reduction in JPS angular errors of ACLR knee at one or more target angles (JPS at 45° but not 15°;[45] JPS at 60° but not 30°;[42] JPS at 15°, 45°, 75°;[44] JPS 20°, 50°, 80°[41]) and/or contralateral non-injured knee (JPS at 30° and 60°[42]) favouring the NT group (exercises on a balance pad,[45] whole-body vibration therapy,[42] neuromotor control exercises [44] or backward treadmill walking[41]). Shen et al. also reported



For peer review only - http://bmjopen.bmj.com/site/about/guidelines.xhtml

improved TTDPM following backward treadmill walking.[41] When we calculated mean differences for author-reported post-operative[42, 44] or change (pre- vs. post-intervention) scores[45] between groups for the ACLR leg with the Review Manager 5.3 software (the Cochrane Collaboration), their 95% confidence intervals revealed no effects (see Table 1 and supplementary files). Moreover, the remaining five studies did not report significant differences in proprioception between groups.[40, 43, 46-48]

Potential reasons for insignificant between-group differences include: 1) experimental and comparator programs (with exercises that are wholly or partly similar) which potentially might stimulate similar effects on proprioception in both programs;[43-48] 2) the exercises did not adequately stimulate proprioception sense;[40] 3) a lack of proprioception deficit following ACL injury (TTDPM similar between ACL-injured and contralateral uninjured knee[48]); 4) a lack of valid, sensitive and responsive knee-specific proprioception test methods; 5) a short follow-up period (a follow-up at least 18 months post-ACLR might be needed to regain proprioceptive function[52]) in most studies except two studies;[44, 48] 6) type II errors arising from low sample sizes in most studies (with missing power or sample size calculations); and 7) adherence rates of participants to the prescribed program (only three studies have explicitly reported adherence rates to training sessions/exercises [Table 1]).[46-48] The heterogeneity of interventions, methodological limitations, inconsistency in the magnitude and direction of effects, and imprecision of effect estimates, found in this review, preclude recommendation of one optimal NT intervention for improving proprioception following ACL injury in clinical practice.

***Risk of Bias in the Included Studies***



For peer review only - http://bmjopen.bmj.com/site/about/guidelines.xhtml

Bias in selection of the reported variables/results due to absence of a pre-specified plan of analyses applied to all but one study,[40] and none had published a trial protocol in a scientific journal although two studies were registered in a trial registry.[40, 41] A possible reason for the absence of registration for most studies in this review may be that all but three studies were older than five years. Yet, for example, the latest published study did not report trial registration.[44]

Another concern was the method used to measure JPS. For instance, estimates of JPS based on 3-5 repetitions  may be insufficient in clinical trials.[53] Similarly, according to Selfe et al., five repetitions in active knee JPS test, and six when performed passively, are necessary to ensure a consistent proprioception score.[54] However, this was only met in two included studies.[42, 44]

Almost all studies used AE for measuring JPS acuity which represents a task-oriented approach to studying performance skill, in contrast to a process-orientation in which underlying processes are in focus. The inconsistency in performance, i.e., response variability (variable error), may reflect noise in sensory signal and its processing[55] and thus be a more process-oriented outcome than AE. To understand possible underlying mechanisms, it would be advantageous to combine task- and process-oriented measures.

In general, method descriptions of proprioception tests were short and, in some studies, deficient, lacking information about factors that could influence the results. One such factor was randomization of the order of target positions (cf. Zult et al.),[40] which is required to minimize the effect of memory and reduce motor elements of the test. This is particularly applicable in tests with active positioning, which was the case for most studies, enabling central motor programs.[56] Inadequate reporting of the proprioception tests would hinder their replication and raise risk of bias rating. Moreover, Kaya et al.  reported only post-intervention JPS scores, precluding baseline scores, despite claiming their study to be an RCT.[44]

For peer review only

### *Mechanisms Underpinning NT Following ACLR*

Two of the included studies evaluated the effects of WBVT;[42, 47] however, only one found a favourable effect on proprioception.[42] Two factors may contribute to the different findings between these studies. First, time point: Fu et al. evaluated JPS 3 months after the intervention, while Moezy et al. did it directly after the intervention period. Second, the use of active[42] or passive[47] knee movement when testing JPS. Active tests stimulate both joint and muscle-tendon mechanoreceptors and induce alpha-gamma co-activation while passive tests assess joint receptors to a higher degree[10, 57] which potentially could mean a higher sensitivity of the active test.

WBVT has shown effects on body posture, flexibility, proprioception (TTDPM in patients with osteoarthritis), coordination and muscle power.[58-60] It has been promoted as an effective method to induce a reflex muscle contraction in subjects with difficulties to evoke voluntary contractions.[61] The mechanism behind the improvements can be that the mechanical stimuli stimulate primary endings of muscle spindles, especially type II fibers, which activate a-motor neurons. This could potentially stimulate central motor command, which facilitates increased muscle activation and voluntary movements.[58]

Cho et al. showed a significant effect on knee proprioception (JPS and TTDPM) with closed kinetic chain exercises on a balance pad/board.[45] Exercises on a balance board are widely used to improve proprioception.[30, 50] In this review, a few NT programs included, amongst other exercises, balance training with or without a balance pad/board.[43-46, 48] Additionally, one study claimed backward walking to stimulate joint/muscle receptors and sensory afferents to the CNS and augment proprioceptive and balance training.[41] Among these studies, all but one,[41] did not show significant mean differences between groups in proprioception calculated using the



For peer review only - http://bmjopen.bmj.com/site/about/guidelines.xhtml

Review-Manager 5.3 software (the Cochrane Collaboration) (see Table 1 and supplementary files). Different designs and levels of difficulty of the execution were found (e.g. a simple static balance task [with and without visual input], dynamic exercises performed on the balance board, backward walking on a treadmill, etc.).

There is a challenge to transfer the rehabilitation in the clinic to automatic movements required for athletic activities.[18, 62] Wii Fit or similar games have the potential to combine feedback with an external focus in a sport-specific environment,[43] supporting the use of such training tools. However, a study on Nintendo Wii Fit training did not support its use for improving knee proprioception following ACLR.[43] Newer technology with stroboscopic-eyewear might have the potential to decrease visual input without fully occluding it, making it possible to use them in sport specific rehabilitation. To prepare the individual for complex athletic environments and reduce re-injury risk, rehabilitation might focuses on NT with reduced demands on visual inputs and enhance automatic movement control with cognitive demands included.[18] Whether such NT training improves knee proprioception and, how this should be assessed in the best way,[13] are yet to be determined.

***The Ability of Tests to Discern Changes in Proprioception Following NT***

There is neither a gold standard proprioception test (targeting JPS, kinaesthesia, force sense) nor a standard procedure with established psychometric properties to test each proprioception sense following ACL injury. In this review, JPS and TTDPM were commonly reported. The Ruffini and Golgi receptors are slow-adapting receptors, responding to a change in joint position. Nevertheless, the Pacinian receptors that respond to low degrees of joint stress are more sensitive to rapid changes in accelerations and contribute to a low TTDPM.[2, 63] JPS has been reported to



For peer review only - http://bmjopen.bmj.com/site/about/guidelines.xhtml

detect a greater difference in knee proprioception than TTDPM following an ACL injury.[2] However, our findings remain equivocal regarding the outcomes of JPS or TTDPM following NT.

Knee-specific proprioception tests provide an indirect measure of proprioception involving the process of the CNS.[10] Psychosocial factors,[64] pain and preinjury motor skills may influence the central mechanisms and the outcome of such tests following NT. Knee-specific proprioception tests are designed to exclude motor skills, but how successful that exclusion works, remains unclear.

***Limitations and Future Recommendations***

The nine included studies examined only individuals with ACLR but not those managed conservatively following ACL injury. Owing to clinical heterogeneity of interventions and outcomes, meta-analyses were precluded from the GRADE synthesis. The included studies had methodological limitations (high risk of bias or some concerns) and only two studies[40, 41] had pre-registered their protocol. There is a need for high quality RCTs with low risk of bias in this area.

The most common reason for exclusion of clinical trials in this review was that they did not evaluate the effects of NT following ACLR with a knee-specific proprioception test. Perhaps, the lack of consensus regarding the most appropriate, valid, reliable and responsive proprioception tests, number of target angles or most responsive target angles (low vs. high) precluded such outcomes in these studies. Therefore, psychometric properties of such tests must be established.[13]



For peer review only - http://bmjopen.bmj.com/site/about/guidelines.xhtml

When designing rehabilitation programs with long-term follow-up, aberrations in neuromotor control and neuroplastic changes should preferably be addressed. To reflect a wide spectrum of individual impairments, further research should investigate differences in individuals with ACL injuries managed with surgical (graft types) or conservative treatment, both sexes, athletes and non-athletes of different ages. Future studies might assess neuromotor control in functional tasks rather than relying on knee-specific proprioception tests, given the challenges of isolating the proprioceptive ability.

## Conclusion

The existing nine studies on individuals with ACLR using heterogeneous interventions and knee-specific proprioception measures revealed a very low certainty in current evidence for employing NT programs to improve knee proprioception. The GRADE evidence synthesis revealed a high risk of bias or some concerns, indirect evidence, conflicting findings, and imprecision of effect estimates in the included studies. Methodologically-robust RCTs with homogenous populations (having ACL injury managed with/without reconstruction), novel/well-designed NT interventions, and valid proprioception measures are warranted to substantiate conclusive evidence in this area.

## Contributors

AA and CKH conceived the idea of the project. AA, MB, SM and CKH were responsible for designing the review and conceptualising the initial review protocol. AA led the writing of the manuscript. MB, SM and CKH contributed to writing the manuscript. AA, MB and CKH have reviewed and revised the manuscript for intellectual content. All authors approved the final version of the manuscript. AA is the guarantor of this work.



For peer review only - http://bmjopen.bmj.com/site/about/guidelines.xhtml

For peer review only

**Funding**

The work was supported by the Swedish Research Council (2017-00892); Region Västerbotten (ALF 7003575; Strategic funding VLL-358901; Project. No. 7002795); the Swedish Research Council for Sports Science (CIF P2019 0068); and King Gustaf V and Queen Victoria's Foundation of Freemasons 2019 (Häger). The funders were not involved in the conception, design, execution and writing of the review.

For peer review only

## References

1. Kapreli E, Athanasopoulos S, Gliatis J, et al. Anterior Cruciate Ligament Deficiency Causes Brain Plasticity: A Functional MRI Study. *Am J Sports Med*. 2009;37:2419-26.

2. Relph N, Herrington L, Tyson S. The effects of ACL injury on knee proprioception: a meta-analysis. *Physiotherapy*. 2014;100:187-95.

3. Sanders TL, Maradit Kremers H, Bryan AJ, et al. Incidence of anterior cruciate ligament tears and reconstruction: a 21-year population-based study. *Am J Sports Med*. 2016;44:1502-7.

4. Kobayashi H, Kanamura T, Koshida S, et al. Mechanisms of the anterior cruciate ligament injury in sports activities: a twenty-year clinical research of 1,700 athletes. *J Sports Sci Med*. 2010;9:669-75.

5. Johnston JT, Mandelbaum BR, Schub D, et al. Video analysis of anterior cruciate ligament tears in professional American football athletes. *Am J Sports Med*. 2018;46:862-8.

6. Acevedo RJ, Rivera-Vega A, Miranda G, Micheo W. Anterior cruciate ligament injury: identification of risk factors and prevention strategies. *Curr Sports Med Rep*. 2014;13:186-91.

7. Ardern CL, Taylor NF, Feller JA, Whitehead TS, Webster KE. Sports participation 2 years after anterior cruciate ligament reconstruction in athletes who had not returned to sport at 1 year: a prospective follow-up of physical function and psychological factors in 122 athletes. *Am J Sports Med*. 2015;43:848-56.

8. Gao F, Zhou J, He C, et al. A Morphologic and Quantitative Study of Mechanoreceptors in the Remnant Stump of the Human Anterior Cruciate Ligament. *Arthroscopy*. 2016;32:273-80.



For peer review only - http://bmjopen.bmj.com/site/about/guidelines.xhtml

9. Dhillon MS, Bali K, Prabhakar S. Differences among mechanoreceptors in healthy and injured anterior cruciate ligaments and their clinical importance. *Muscles Ligaments Tendons J*. 2012;2:38-43.

10. Röijezon U, Clark NC, Treleaven J. Proprioception in musculoskeletal rehabilitation. Part 1: Basic science and principles of assessment and clinical interventions. *Man Ther*. 2015;20:368-77.

11. Kapreli E, Athanasopoulos S. The anterior cruciate ligament deficiency as a model of brain plasticity. *Med Hypotheses*. 2006;67:645-50.

12. Nakamae A, Adachi N, Ishikawa M, Nakasa T, Ochi M. No evidence of impaired proprioceptive function in subjects with anterior cruciate ligament reconstruction: a systematic review. *J ISAKOS*. 2017;2:191.

13. Arumugam A, Strong A, Tengman E, Röijezon U, Häger CK. Psychometric properties of knee proprioception tests targeting healthy individuals and those with anterior cruciate ligament injury managed with or without reconstruction: a systematic review protocol. *BMJ Open*. 2019;9:e027241.

14. Stensdotter AK, Tengman E, Olofsson LB, Häger C. Deficits in single-limb stance more than 20 years after ACL injury. *European Journal of Physiotherapy*. 2013;15:78-85.

15. Fukunaga T, Johnson CD, Nicholas SJ, McHugh MP. Muscle hypotrophy, not inhibition, is responsible for quadriceps weakness during rehabilitation after anterior cruciate ligament reconstruction. *Knee Surgery, Sports Traumatology, Arthroscopy*. 2019;27:573-9.

16. Zebis MK, Andersen LL, Bencke J, Kjaer M, Aagaard P. Identification of athletes at future risk of anterior cruciate ligament ruptures by neuromuscular screening. *Am J Sports Med*. 2009;37:1967-73.



For peer review only - http://bmjopen.bmj.com/site/about/guidelines.xhtml

17. Grooms DR, Page SJ, Nichols-Larsen DS, Chaudhari AM, White SE, Onate JA. Neuroplasticity Associated With Anterior Cruciate Ligament Reconstruction. *J Orthop Sports Phys Ther*. 2017;47:180-9.

18. Grooms D, Appelbaum G, Onate J. Neuroplasticity Following Anterior Cruciate Ligament Injury: A Framework for Visual-Motor Training Approaches in Rehabilitation. *J Orthop Sports Phys Ther*. 2015;45:381-93.

19. Clark NC, Röijezon U, Treleaven J. Proprioception in musculoskeletal rehabilitation. Part 2: clinical assessment and intervention. *Man Ther*. 2015;20:378-87.

20. Risberg MA, Mork M, Jenssen HK, Holm I. Design and implementation of a neuromuscular training program following anterior cruciate ligament reconstruction. *J Orthop Sports Phys Ther*. 2001;31:620-31.

21. Moutzouri M, Gleeson N, Billis E, Panoutsopoulou I, Gliatis J. What is the effect of sensori-motor training on functional outcome and balance performance of patients' undergoing TKR? A systematic review. *Physiotherapy*. 2016;102:136-44.

22. Ordahan B, Kucuksen S, Tuncay I, Salli A, Ugurlu H. The effect of proprioception exercises on functional status in patients with anterior cruciate ligament reconstruction. *J Back Musculoskelet Rehabil*. 2015;28:531-7.

23. Moher, D., Liberati, A., Tetzlaff, J., Altman, D. G., & Prisma Group. (2009). Preferred reporting items for systematic reviews and meta-analyses: the PRISMA statement. *PLoS med.* 2009;6:e1000097.

24. Methley AM, Campbell S, Chew-Graham C, McNally R, Cheraghi-Sohi S. PICO, PICOS and SPIDER: a comparison study of specificity and sensitivity in three search tools for qualitative systematic reviews. *BMC Health Serv Res.* 2014;14:579.

For peer review only



For peer review only - http://bmjopen.bmj.com/site/about/guidelines.xhtml

25. Sterne JAC, Savović J, Page MJ, et al. RoB 2: a revised tool for assessing risk of bias in randomised trials. *BMJ.* 2019;366.

26. Guyatt GH, Oxman AD, Vist GE, et al. GRADE: an emerging consensus on rating quality of evidence and strength of recommendations. *BMJ.* 2008;336:924-6.

27. Laboute E, Verhaeghe E, Ucay O, Minden A. Evaluation kinaesthetic proprioceptive deficit after knee anterior cruciate ligament (ACL) reconstruction in athletes. *J Exp Orthop.* 2019;6:1-7.

28. Feil S, Newell J, Minogue C, Paessler HH. The effectiveness of supplementing a standard rehabilitation program with, superimposed neuromuscular electrical stimulation after anterior cruciate ligament reconstruction. *Am J Sports Med*. 2011;39:1238-47.

29. Ihara H, Nakayama A. Dynamic joint control training for knee ligament injuries. *Am J Sports Med*. 1986;14:309-15.

30. Vathrakokilis K, Malliou P, Gioftsidou A, Beneka A, Godolias G. Effects of a balance training protocol on knee joint proprioception after anterior cruciate ligament reconstruction. *J Back Musculoskelet Rehabil.* 2008;21:233-7.

31. Saha S, Adhya B, Dhillon MS, Saini A. A Study on the Role of Proprioceptive Training in Non Operative ACL Injury Rehabilitation. *Indian J Physiother Occup Ther.* 2015;9:226-31.

32. Lim J-M, Cho J-J, Kim T-Y, Yoon B-C. Isokinetic knee strength and proprioception before and after anterior cruciate ligament reconstruction: A comparison between home-based and supervised rehabilitation. *J Back Musculoskelet Rehabil.* 2019;32:421-9.

33. Liu-Ambrose T, Taunton JE, MacIntyre D, McConkey P, Khan KM. The effects of proprioceptive or strength training on the neuromuscular function of the ACL reconstructed knee: a randomized clinical trial. *Scand J Med Sci Sports.* 2003;13:115-23.



For peer review only - http://bmjopen.bmj.com/site/about/guidelines.xhtml

34. Lee SJ, Ren Y, Chang AH-I, Geiger F, Zhang L-Q. Effects of pivoting neuromuscular training on pivoting control and proprioception. *Med Sci Sports Exerc.* 2014;46:1400-9.

35. Peultier-Celli L, Mainard D, Wein F, et al. Comparison of an innovative rehabilitation, combining reduced conventional rehabilitation with balneotherapy, and a conventional rehabilitation after anterior cruciate ligament reconstruction in athletes. *Front Surg.* 2017;4:61.

36. Faggal MS, Abdelsalam MS, Adel Elhakk SM, Mahmoud NF. Proprioceptive training after ACL reconstruction: Standard versus stump preservation technique. *Physiother Pract Res.* 2019;40:69-75.

37. San Martín-Mohr C, Cristi-Sánchez I, Pincheira PA, Reyes A, Berral FJ, Oyarzo C. Knee sensorimotor control following anterior cruciate ligament reconstruction: A comparison between reconstruction techniques. *PLoS One*. 2018;13:e0205658.

38. Büyükturan Ö, Büyükturan B, Kurt EE, Yetis M. Effects of Tai Chi on partial anterior cruciate ligament injury: A single-blind, randomized-controlled trial. *Turk J Phys Med Rehabil.* 2019;65:160-8.

39. Wang L. Immediate effects of neuromuscular joint facilitation intervention after anterior cruciate ligament reconstruction. *J Phys Ther Sci.* 2016;28:2084-7.

40. Zult T, Gokeler A, van Raay J, et al. Cross-education does not accelerate the rehabilitation of neuromuscular functions after ACL reconstruction: a randomized controlled clinical trial. *Eur J Appl Physiol*. 2018;118:1609-23.

41. Shen M, Che S, Ye D, Li Y, Lin F, Zhang Y. Effects of backward walking on knee proprioception after ACL reconstruction. *Physiother Theory Pract.* 2019.

42. Moezy A, Olyaei G, Hadian M, Razi M, Faghihzadeh S. A comparative study of whole body vibration training and conventional training on knee proprioception and postural stability



For peer review only - http://bmjopen.bmj.com/site/about/guidelines.xhtml

after anterior cruciate ligament reconstruction. *British journal of sports Medicine*. 2008;42:373-85.

43. Baltaci G, Harput G, Haksever B, Ulusoy B, Ozer H. Comparison between Nintendo Wii Fit and conventional rehabilitation on functional performance outcomes after hamstring anterior cruciate ligament reconstruction: prospective, randomized, controlled, double-blind clinical trial. *Knee Surg Sports Traumatol Arthrosc*. 2013;21:880-7.

44. Kaya D, Guney-Deniz H, Sayaca C, Calik M, Doral MN. Effects on Lower Extremity Neuromuscular Control Exercises on Knee Proprioception, Muscle Strength, and Functional Level in Patients with ACL Reconstruction. *Biomed Res Int.* 2019:1-7.

45. Cho SH, Bae CH, Gak HB. Effects of closed kinetic chain exercises on proprioception and functional scores of the knee after anterior cruciate ligament reconstruction. *J Phys Ther Sci.* 2013;25:1239-41.

46. Risberg MA, Holm I, Myklebust G, Engebretsen L. Neuromuscular training versus strength training during first 6 months after anterior cruciate ligament reconstruction: a randomized clinical trial. *Phys Ther*. 2007;87:737-50.

47. Fu CLA, Yung SHP, Law KYB, et al. The effect of early whole-body vibration therapy on neuromuscular control after anterior cruciate ligament reconstruction: a randomized controlled trial. *Am J Sports Med*. 2013;41:804-14.

48. Beynnon BD, Johnson RJ, Naud S, et al. Accelerated versus nonaccelerated rehabilitation after anterior cruciate, ligament reconstruction:A prospective, randomized, double-blind investigation evaluating knee joint laxity using roentgen, stereophotogrammetric analysis. *Am J Sports Med*. 2011;39:2536-48.



For peer review only - http://bmjopen.bmj.com/site/about/guidelines.xhtml

49. Hortobágyi T, Garry J, Holbert D, Devita P. Aberrations in the control of quadriceps muscle force in patients with knee osteoarthritis. Arthritis Care Res. 2004;51:562-9.

50. Cooper RL, Taylor NF, Feller JA. A systematic review of the effect of proprioceptive and balance exercises on people with an injured or reconstructed anterior cruciate ligament. *Res Sports Med.* 2005;13:163-78.

51. Baltaci G, Kohl HW. Does proprioceptive training during knee and ankle rehabilitation improve outcome? *Phys Ther Rev.* 2003;8:5-16.

52. Iwasa J, Ochi M, Adachi N, Tobita M, Katsube K, Uchio Y. Proprioceptive improvement in knees with anterior cruciate ligament reconstruction. *Clin Orthop Relat Res*. 2000:168-76.

53. Han J, Waddington G, Adams R, Anson J, Liu Y. Assessing proprioception: A critical review of methods. *J Sport Health Sci*. 2016;5:80-90.

54. Selfe J, Callaghan M, McHenry A, Richards J, Oldham J. An investigation into the effect of number of trials during proprioceptive testing in patients with patellofemoral pain syndrome. *J Orthop Res.* 2006;24:1218-24.

55. van Beers RJ, Sittig AC, van der Gon Denier JJ. How humans combine simultaneous proprioceptive and visual position information. *Exp Brain Res.* 1996;111:253-61.

56. Gandevia SC, Burke D. Does the nervous system depend on kinesthetic information to control natural limb movements? *Behav Brain Sci.* 1992;15:614-.

57. Clark NC, Akins JS, Heebner NR, et al. Reliability and measurement precision of concentric-to-isometric and eccentric-to-isometric knee active joint position sense tests in uninjured physically active adults. *Phys Ther Sport*. 2016;18:38-45.

For peer review only



For peer review only - http://bmjopen.bmj.com/site/about/guidelines.xhtml

58. Avelar NC, Costa SJ, da Fonseca SF, et al. The effects of passive warm-up vs. whole-body vibration on high-intensity performance during sprint cycle exercise. *J Strength Cond Res*. 2012;26:2997-3003.

59. Chow DHK, Lee TY, Pope MH. Effects of whole body vibration on spinal proprioception in healthy individuals. *Work*. 2018.

60. Trans T, Aaboe J, Henriksen M, Christensen R, Bliddal H, Lund H. Effect of whole body vibration exercise on muscle strength and proprioception in females with knee osteoarthritis. *Knee*. 2009;16:256-61.

61. Herrero AJ, Menendez H, Gil L, et al. Effects of whole-body vibration on blood flow and neuromuscular activity in spinal cord injury. *Spinal Cord*. 2011;49:554-9.

62. Benjaminse A, Gokeler A, Dowling AV, et al. Optimization of the anterior cruciate ligament injury prevention paradigm: novel feedback techniques to enhance motor learning and reduce injury risk. *J Orthop Sports Phys Ther*. 2015;45:170-82.

63. Ochi M, Iwasa J, Uchio Y, Adachi N, Sumen Y. The regeneration of sensory neurones in the reconstruction of the anterior cruciate ligament. *J Bone Joint Surg Br*. 1999;81:902-6.

64. Louw A, Zimney K, Puentedura EJ, Diener I. The efficacy of pain neuroscience education on musculoskeletal pain: A systematic review of the literature. *Physiother Theory Pract*. 2016;32:332-55.

For peer review only



For peer review only - http://bmjopen.bmj.com/site/about/guidelines.xhtml

**Table 1. Summary of study characteristics**

| Study citation | Sample size[a], age (mean ± SD), gender; ACLD/ACLR (Graft) | Intervention; Adherence to prescribed exercises/ training | Comparator; Adherence to prescribed exercises/ training | Knee-specific proprioception test; outcome | Between-group (experimental vs. control) comparisons of ACL-injured (reconstructed) limb - mean difference (95% confidence interval)[b] |
|---|---|---|---|---|---|
| **Baltaci et al. (2013)** [43] | Exp: n=15, 28.6±6.8 years, 15 men; Com: n=15, 29.3±5.7 years, 15 men; ACLR (hamstring tendon graft). | Nintendo Wii Fit training: 3 times/week; 60 min/session; from week 1-12 after ACLR. **Adherence:** NR | Conventional rehabilitation: Week 1-12 after ACLR; **Adherence:** NR | **Proprioception test:** JPS (ipsilateral replication method); **Body position:** NR; **Instrument:** Monitored Rehab System with a leg press machine and a computer game; **Procedure:** Active-active, with and without blindfolding of the eyes (2 trials each); **Starting angle (SA):** NR; **Target angle (TA):** NR; **Outcome measure:** absolute angular error (AAE; difference between visual and non-visual results for each leg) | **JPS (°)[c] at 12 weeks post-intervention:** 1.90 [-31.20, 35.00] 33.30 [-28.02, 94.62] |

For peer review only

| | | | | | |
|---|---|---|---|---|---|
| **Beynnon et al. (2011)** [48] | Int: n=19,<br>29.7±10.1 years,<br>13 males, 6 females;<br>Com: n=17,<br>30.2±9.9 years,<br>9 males, 8 females;<br>ACLR (patellar tendon graft) | Accelerated rehabilitation: daily exercises at home + 3 times/week exercises under supervision from week 1-19 after ACLR;<br>**Adherence:** 94% (range, 25%-292%) over 19 weeks | Non-accelerated rehabilitation: daily exercises at home + 3 times/week exercises under supervision from Week 1-32 after ACLR;<br>**Adherence:** 53% (range, 13%-108%) over 32 weeks | **Proprioception test:** TTDPM;<br>**Body position:** Seated;<br>**Instrument:** A customized joint motion detection system;<br>**Procedure:** passive movement of the knee into flexion or extension (3 trials for both ACL-reconstructed and contralateral uninjured knees) with eyes blindfolded;<br>**SA:** NR;<br>**Angular velocity:** 0.1°/s;<br>**Outcome measure:** Threshold angle (difference between the initial angle [SA] and the angle at which the test was stopped) to detect passive knee motion into flexion or extension (mean of the three trials in one direction). | **TTDPM (°)[c] at 24 months post-ACLR:**<br>**SA (NR):**<br>0.09 [-0.42, 0.60] |
| **Cho et al. (2013)** [45] | Int: n=14,<br>29.92±5.46 years; 14 males;<br>Com: n=14,<br>28.78±7.24 years; 14 males;<br>ACLR (NR). | Unstable exercise group: exercises performed on a balance pad or balance board; 60 min/session; 3 times/week early after injury, for 6 weeks;<br>**Adherence:** NR | Stable exercise group: exercises performed on a stable floor: 3 times/week Early after injury, for 6 weeks;<br>**Adherence:** NR | **Proprioception test:** JPS;<br>**Body position:** seated (?);<br>**Instrument:** Biodex dynamometer;<br>**Procedure:** NR-active, with eyes blindfolded;<br>**SA:** 90°;<br>**TA:** 15°, 45°;<br>**Outcome measure:** AAE (mean of the three trials at each angle). | **JPS (°)[d] at 6 weeks post intervention:**<br>TA 15°:<br>0.14 [-0.69, 0.97]<br>TA 45°:<br>-0.87 [-1.91, 0.17] |

| | | | | | |
|---|---|---|---|---|---|
| **Fu et al. (2013)** [47] | Int: n=24, 23.3±5.2 years; Com: n=24, 25.2±7.3 years; ACLR (hamstring graft). | Conventional rehabilitation program + Whole-body vibration therapy: 2 times/week from week 5-13 after ACLR; **Adherence:** 83.2% over 12 weeks | Conventional rehabilitation program: week 5-13 after ACLR; **Adherence:** 84.4% over 12 weeks | **Proprioception test:** JPS; **Body position:** seated; **Instrument:** Biodex dynamometer; **Procedure:** passive-passive, eyes blindfolded; **SA:** 90°; **TA:** 30°, 60°; **Outcome measure:** AAE (mean of the three trials at each angle) | **JPS (°)[c] at 6 months post-ACLR:** **TA 30°:** -0.82 [-2.69, 1.05] **TA 60°:** -0.70 [-2.31, 0.91] |
| **Kaya et al. (2019)** [44] | Int (Group 1): n=20; 29.35±9.71 years; 20 males; Com (Group 2): n=20; 31.60±8.45 years; 20 males; ACLR (tibialis anterior allograft). | Standard rehabilitation program (0-2 weeks) + neuromuscular control exercises (3-36 weeks); **Adherence:** NR | Standard rehabilitation program (0-36 weeks); **Adherence:** NR | **Proprioception test:** JPS; **Body position:** seated (?); **Instrument:** Biodex dynamometer; **Procedure:** passive-active, eyes blindfolded; **SA:** 90°; **TA:** 15°, 45°, 75°; **Outcome measure:** AAE (mean of six trials at each angle) | **JPS (°)[c] at 24 months post-ACLR:** **TA 15°:** -1.51 [-3.30, 0.28] **TA 45°:** -1.69 [-5.06, 1.68] **TA 75°:** -1.30 [-3.34, 0.74] |
| **Moezy et al. (2008)** [42] | Int: n=12, 24.51±3.38 years; Com: n=11, 22.70±3.77 years; ACLR (patellar tendon graft) | Whole-body vibration therapy: 3 times/week from week 12-16 after ACLR; **Adherence:** | Conventional strengthening exercises program: 3 sessions/week Week 12-16 after ACLR; | **Proprioception test:** JPS; **Body position:** seated; **Instrument:** Biodex dynamometer; **Procedure:** active-active, eyes blindfolded; **SA:** 90°; **TA:** 30°, 60°; | **JPS (°)[e,d] at 16 weeks post-ACLR:** **TA 30°:** 1.66 [-0.40, 3.72] **TA 60°:** 3.03 [1.54, 4.52] |

For peer review only

| | | | | | |
|---|---|---|---|---|---|
| | | NR | **Adherence:** NR | **Outcome measure:** AAE (mean of five trials at each angle for both ACL-reconstructed and contralateral uninjured knees) | |
| **Risberg et al. (2007)** [46] | Int: n = 39; 3 females - 27.2 (range: 20.6-37.9) years and 26 males - 27.7 (16.7-39.6) years; Com: n=35, 14 females - 26.5 (19.8-38.0) years and 21 males - 31.2 (19.4-40.3) years; ACLR (patellar tendon graft) | Neuromuscular training program: 2-3 times/week from week 1-24 after ACLR; **Adherence:** 71% over ~20 weeks | Traditional strength training: 2-3 times/week from week 1-24 after ACLR; **Adherence:** 91% over ~20 weeks | **Proprioception test:** TTDPM; **Body position:** NR; **Instrument:** a customized TTDPM device; **Procedure:** passive movement of the knee into flexion and extension (three trials for each direction for both ACL-injured knees and contralateral uninjured knees); no information on blindfolding of eyes; **SA:** 15°; **Angular velocity:** 0.5°/s; **Outcome measure:** threshold angle to detect passive knee motion into flexion and extension (mean of three trials for each angle in each direction). | **TTDPM (°)[c] at 6 months post-ACLR:** **SA 15°:** -0.02 [-0.39, 0.35] (Note: TTDPM data were available only for the first 47 participants out of 74 in total). |
| **Shen et al. (2019)** [41] | Int (A): n=10; 36.6±12.1 years; 5 male, 5 females. Int (B): n=11; 37.5±9.39 years; 6 male, 5 females. Int (C): n=11; 34±10.29 years; 7 male, 4 females. | Standard rehabilitation + backward walking on the treadmill: Int. groups A, B, C, and D underwent backward walking | Standard rehabilitation with range of motion exercises, power exercises, walking, and cycling (duration and other | **Proprioception test 1:** JPS; **Body position:** supine lying; **Instrument:** continuous passive motion device; **Procedure:** passive-passive, eyes blindfolded; **SA:** 0°; **TA:** 20°, 50°, 80°, **Outcome measure:** AAE (mean of the three trials at each angle for ACL-injured knees?). | **Int (A) vs. Com group at 4 weeks post-intervention[d]:** **JPS (°)[c]:** **TA 20°:** -1.40 [-2.59, -0.21] **TA 50°:** -1.36 [-2.35, -0.37] **TA 80°:** -1.28 [-2.31, -0.25] **TTDPM (°)[c]:** |

| | | | | | |
|---|---|---|---|---|---|
| | Int (D): (n=10); 32.9±11.45 years; 6 male, 4 females.<br>Com: n=10; 35.5±10.1 years; 7 male, 3 females;<br><br>ACLR (patellar tendon graft, hamstring tendon graft, allograft) | training at 1.3 km/h at different inclination angles of the treadmill (0°, 5°, 10°, and 15°, respectively); 20 min/day, 5 days/week for 4 weeks;<br>**Adherence:** NR | parameters: NR);<br>**Adherence:** NR | **Proprioception test 2:** TTDPM;<br>**Body position:** Supine lying;<br>**Instrument:** continuous passive motion device;<br>**Procedure:** passive movement of the knee into flexion (3 times for each angle for ACL-injured knees?) with eyes blindfolded;<br>**SA:** 20°, 50°, 80°;<br>**Angular velocity:** 1°/s;<br>**Outcome measure:** Threshold angle to detect passive knee motion into flexion (mean of three trials for each angle in one direction). | **SA 20°:**<br>-1.34 [-2.11, -0.57]<br>**SA 50°:**<br>-1.40 [-2.05, -0.75]<br>**SA 80°:**<br>-1.29 [-2.00, -0.58] |
| **Zult et al. (2018)** [40] | Int: n =29 (22), 28±9 years;<br>Com: n = 26 (21), 28±10 years<br>n=24 males n=20 females<br><br>ACLR (patellar tendon graft/ hamstring tendon graft (SSG)/ Artificial) | Standard rehabilitation + Strength training of the quadriceps of the non-injured leg; 2 quadriceps exercises, 8–12 reps. maximum, 3 sets; 2 times/week from week 1-12 after ACLR;<br>**Adherence:** | Standard rehabilitation: 2 times/week from week 1-12 after ACLR;<br>**Adherence:** NR explicitly; however, two participants who performed <26 sessions were excluded from analysis | **Proprioception test 1:** JPS[g]<br>**Body position:** seated (?);<br>**Instrument:** Biodex dynamometer (?);<br>**Procedure:** passive-active, eyes blindfolded (?);<br>**SA:** 90° (?);<br>**TA:** 15°, 30°, 45°, and 60°;<br>**Outcome measure:** AAE (one trial at each angle).<br><br>**Proprioception test 2:** Quadriceps force control (QFC);<br>**Body position:** seated (?);<br>**Instrument:** Biodex dynamometer (?);<br>**Procedure:** A target force matching task with the target set at 20% MVC for | **JPS (°)[e] at 26 weeks post-ACLR:**<br>**TA 15°:**<br>1.00 [-1.12, 3.12]<br>**TA 30°:**<br>2.00 [-0.12, 4.12]<br>**TA 45°:**<br>-1.00 [-3.39, 1.39]<br>**TA 60°:**<br>-1.00 [-2.79, 0.79]<br><br>**QFC (Nm)[e,f] at 26 weeks post-ACLR:**<br>**Concentric 60°/s:**<br>6.00 [0.67, 11.33]<br>**Eccentric 60°/s:** |

For peer review only

| | | | | |
|---|---|---|---|---|
| | NR explicitly; however, one participant who performed <26 sessions was excluded from analysis after week 26 | after week 26 | three isometric trials (at 65° of knee flexion [5 s duration]) and 40 Nm for dynamic trials (four concentric and eccentric trials at 20°/s from 10°-90° knee flexion) (20°/s between 10° and 90° of knee flexion);<br>**Outcome measure:** force accuracy (absolute error) determined over the terminal 3 s data for isometric trials (at 65° knee flexion) and over the middle 2 s data for concentric and eccentric trials. | -1.00 [-3.99, 1.99]<br>**Isometric:**<br>1.00 [-0.76, 2.76] |

[a]Included in ana

[b]Calculated with Review Manager (RevMan) 5.3 (The Cochrane Collaboration 2014, Nordic Cochrane Centre Copenhagen, Denmark);

[c]Mean difference between groups were calculated based on post-intervention/final follow-up scores reported by the authors;

[d]Difference between four intervention groups and the comparator group were same and so only one comparison is presented.

[e]Mean difference between groups were calculated based on change scores from baseline (pre- vs. post-intervention) reported by the authors;

[f]Quadriceps force accuracy; both legs (within each group) showed improved force control (22–34%) at 26 weeks post-surgery ($p < 0.050$) according to the authors;

[g]JPS method has been presumed based on authors' reference to the method employed by Hortobagyi et al. [49];

ACLR - anterior cruciate ligament reconstruction, Int – intervention group; com – comparator group; JPS - joint position sense, NR- not reported, TTDPM - threshold to detection of passive motion, min. - minutes, reps – repetitions.

For peer review only

**Table 2. Risk of bias assessment of included studies according to the Revised Cochrane risk-of-bias tool for randomized trials (RoB 2) - judgements in five domains and an overall judgement using the descriptors of low risk of bias (low), some concerns, and high risk of bias (High).**

| Included studies | Outcome variable | 1. Bias from the randomization process | 2. Bias due to deviations from intended interventions | 3. Bias due to missing outcome data | 4. Bias in measurement of the outcome | 5. Bias in selection of the reported result | O ju |
|---|---|---|---|---|---|---|---|
| **Baltaci et al. 2013** [43] | JPS | High | Some concerns | Low | High | High | H |
| **Beynnon et al. 2011** [48] | TTDPM | Low | Low | Low | Low | Some concerns | So |
| **Cho et al. 2013**[45] | JPS | Some concerns | Some concerns | Low | High | Some concerns | H |
| **Fu et al. 2013**[47] | JPS | Low | Low | Low | Low | Some concerns | So |
| **Kaya et al. 2019**[44] | JPS | Some concerns | High | High | Low | Some concerns | H |
| **Moezy et al. 2008**[42] | JPS | Some concerns | Low | Low | Some concerns | High | H |
| **Risberg et al. 2007** [46] | TTDPM | Low | Low | Low | Low | Some concerns | So |
| **Shen et al. 2019**[41] | JPS | Some concerns | Low | Low | Low | Some concerns | So |
| | TTDPM | Some concerns | Low | Low | Low | Some concerns | So |
| **Zult et al. 2018**[40] | JPS | Low | Some concerns | Low | High | Some concerns | H |
| | QFC | Low | Some concerns | Low | Low | Some concerns | So |

JPS - joint position sense, TTDPM - threshold to detect passive motion, QFC - quadriceps force control.

For peer review only

**Table 3. Applying the GRADE approach to rate the certainty in evidence found in the review**

| Certainty assessment | | | | | | | № of patients | | Certainty |
|---|---|---|---|---|---|---|---|---|---|
| № of studies | Study design | Risk of bias | Inconsistency | Indirectness | Imprecision | Other considerations | Neuromuscular Training | Comparator Intervention | |
| **Knee joint position sense (JPS)** | | | | | | | | | |
| 7 | Randomized trials | very serious[a] | serious[b] | serious[c] | serious[d] | none | 139 | 105 | ⊕◯◯◯ VERY LOW |
| **Knee joint threshold to detect passive motion (TTDPM)** | | | | | | | | | |
| 3 | Randomized trials | serious[a] | serious[b] | serious[c] | serious[d] | none | 84 | 51 | ⊕◯◯◯ VERY LOW |
| **Quadriceps force control (QFC)** | | | | | | | | | |
| 1 | Randomized trial | serious[a] | serious[e] | not serious | serious[e] | none | 22 | 21 | ⊕◯◯◯ VERY LOW |

Note: GRADE domains are explained further in Tables 5 and 6.

a. Included studies had a high risk of bias or some concerns based on the Cochrane ROB2 tool;
b. The direction and/or magnitude of effect was inconsistent across trials;
c. Clinical heterogeneity (of participants, interventions, and method of assessing outcome measures);
d. Number of participant <400 and/or wide 95% confidence intervals of effect size estimates;
e. Available population, the magnitude and direction of effect, and effect estimates come from only one study.

For peer review only

**Table 4. GRADE evaluation of the certainty in evidence for knee joint position sense (JPS) and threshold to detect passive motion (TTDPM) following neuromuscular training in individuals with anterior cruciate ligament reconstruction**

| GRADE domain | Reviewer judgment | Concerns about GRADE domains |
|---|---|---|
| | **Knee joint position sense (JPS)** | |
| **Risk of bias (methodological limitations)** | Among seven RCTs[40-45, 47] reporting changes in JPS following neuromuscular training, five RCTs were found to have a high risk of bias while the remaining two studies have some concerns based on the Cochrane ROB 2 tool (see Table 4). Indeed, we judged that the included RCTs have very serious methodological limitations. | Very serious |
| **Inconsistency** | The direction and/or magnitude of effect on JPS was inconsistent across most of the included RCTs. In summary, the between-group comparisons of five RCTs showed borderline or no change in JPS angular errors of the ACLR knee for one or more target angles following interventions. We noted significant differences in reduction of JPS angular errors for all target angles favoring the intervention groups (backward treadmill walking or motor control exercises) in only two RCTs as reported by the authors.[41, 44] In fact, Kaya et al. (2019) had reported only post-intervention scores but they neither reported nor compared the baseline scores (post-operative scores).[44] Two other studies [42, 45] presented with insignificant effects at a low target angle (15° or 30°) and significant effects at a high target angle (45° or 60°) of JPS favoring the intervention group (whole-body vibration therapy[42] or exercises on a balance pad[45]). When we calculated mean differences for author-reported post-operative[44] or change (pre- vs. post-intervention) scores,[42, 45] between groups for the ACLR leg with the Review Manager 5.3 software (the Cochrane Collaboration), their 95% confidence intervals revealed no effects. Overall, we judged the evidence to have serious inconsistency in the direction and/or magnitude of effects. | Serious |
| **Indirectness** | The participants (with ACLR [different grafts]), different neuromuscular training and comparator interventions, and knee specific JPS measures in the included studies provide evidence to the research question. However, the heterogeneity of interventions precludes recommendation of one optimal neuromuscular training intervention for | Serious |

For peer review only

| | | |
|---|---|---|
| | clinical practice. In addition, variations in the methods of JPS measurements (active vs. passive angle reproduction, low vs. high target angles, etc.) precluded a meta-analysis. However, forest plots have been presented for easy understanding of the confidence intervals and overall findings on JPS following neuromuscular rehabilitation training in individuals with ACLR (see appendices). We judged the evidence to have serious indirectness especially owing to variations in the interventions and outcome measures. | |
| **Imprecision** | A total of 244 patients was included from seven RCTs reporting changes in JPS following neuromuscular training (n = 139) or comparator interventions (n = 105). Most of the included trails reported non-significant results with wider 95% confidence intervals for one or more JPS (target) angles (see appendices). Therefore, we judged the evidence to have serious imprecision. | Serious |
| **Publication bias** | Since negative and positive findings have been published, and a comprehensive search for RCTs has been done, we did not suspect a publication bias. | None |
| | **Knee joint threshold to detect passive motion (TTDPM)** | |
| **Risk of bias (methodological limitations)** | Three RCTs[20, 41, 48] reporting changes in TTDPM following neuromuscular training were found to show some concerns in risk of bias based on the Cochrane ROB 2 tool (see Table 4). We judged the included RCTs to be of serious methodological limitations. | Serious |
| **Inconsistency** | The direction and/or magnitude of effect was conflicting between the three RCTs. As two trials reported insignificant effects and one[41] reported significant effects (see appendices), we judged the evidence to have serious inconsistency in the direction and/or magnitude of effects. | Serious |
| **Indirectness** | The participants (with ACLR [different grafts]), different neuromuscular training and comparator interventions, and knee specific TTDPM measures in the included studies provide some evidence to the research question in hand. However, the heterogeneity of interventions and TTDPM measurements (starting angles, angular velocity, etc.) precluded a meta-analysis. We judged the evidence to have serious indirectness especially owing to variations in the interventions and TTDPM methods. | Serious |

For peer review only

| | | |
|---|---|---|
| **Imprecision** | A total of 135 patients was included in three RCTs reporting the effects of neuromuscular training (n = 84) or comparator interventions (n = 51) on TTDPM. Two trails[46, 48] reported non-significant results while another one[41] reported significant effects which is evident with their confidence intervals (see appendices). However, Shen et al. (2019) reporting significant effects on TTDPM included only 10 to 11 participants in each group while the other two studies with a relatively larger sample size declared no significant effects on TTDPM. Therefore, we judged the evidence to have serious imprecision. | Serious |
| **Publication bias** | As both negative and positive findings have been published, and a comprehensive search for RCTs has been done, we did not suspect a publication bias. | None |

Note: Effect size estimates – a forest plot (see supplementary files) has been presented for easy understanding of the mean differences between groups and corresponding 95% confidence intervals related to outcome measures; however, a meta-analysis was not formally included in the review owing to heterogeneity of the interventions and JPS/TTDPM methods.

For peer review only

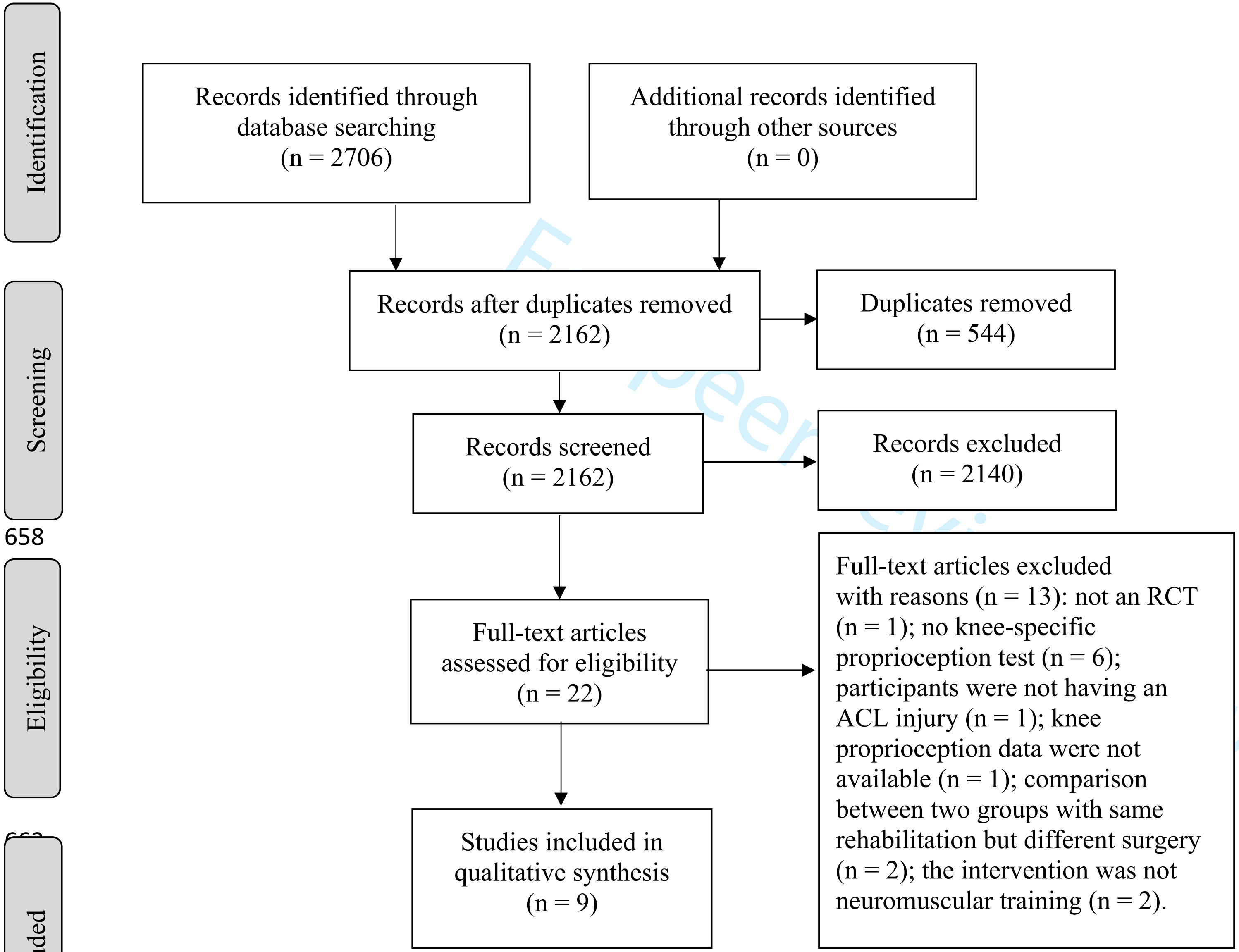


Figure 1. Flow diagram depicting the steps involved in screening and selection of eligible articles

**Online supplemental file 1.**

**Database-specific search strategies**

**AMED**

(Propriocep* OR (ZU "proprioception") OR Kinesthe* OR (ZU "kinesthesis") OR sensorimotor OR sensory-motor OR "joint position sense" OR "joint position detection" OR "threshold to detect passive motion" OR "passive motion direction discrimination" OR "passive motion detection threshold" OR "threshold for motion detection" OR "threshold hunting" OR "detection threshold" OR "discrimination threshold" OR "ipsilateral matching" OR "contralateral matching" OR "joint angle error" OR "distance estimation error" OR "passive recognition" OR "direction accuracy" OR "active reproduction" OR "joint reposition" OR "force sense" OR "force perception" OR "velocity sense" OR "active movement extent discrimination") AND (S1 AND S2 AND S3 AND S4)

S1: “Anterior Cruciate Ligament” OR (ZU "anterior cruciate ligament") OR “Knee joint” OR (ZU "knee joint)

S2: Injur* OR (ZU "injuries") OR (ZU "anterior cruciate ligament injuries") OR Reconstruction OR (ZU "anterior cruciate ligament reconstruction") OR

S3: Propriocep* OR (ZU "proprioception") OR Neuromuscular OR sensorimotor OR sensory-motor OR "Kinetic chain" OR (ZU "kinetics") OR Coordination OR Balance OR (ZU "balance") OR Plyometric (ZU "plyometric exercise") OR Vibration OR (ZU "vibration") OR Exercise* OR (ZU "exercise") OR Intervention OR Training OR Rehabilitation OR (ZU "rehabilitation") OR Therap* OR (ZU "therapy") OR Treatment

S4: Propriocep* OR (ZU "proprioception") OR Kinesthe* OR (ZU "kinesthesis") OR sensorimotor OR sensory-motor OR "joint position sense" OR "joint position detection" OR "threshold to detect passive motion" OR "passive motion direction discrimination" OR "passive motion detection threshold" OR "threshold for motion detection" OR "threshold hunting" OR "detection threshold" OR "discrimination threshold" OR "ipsilateral matching" OR "contralateral matching" OR "joint angle error" OR "distance estimation error" OR "passive recognition" OR "direction accuracy" OR "active reproduction" OR "Joint reposition" OR "force sense" OR "force perception" OR "velocity sense" OR "active movement extent discrimination"

Limiters - Language: English, Expanders - Apply related words, Search modes - Find any of my search terms, Interface - EBSCOhost Research Databases, Search Screen - Advanced Search, Database - AMED - The Allied and Complementary Medicine Database

**CINAHL**

Limiters - Peer Reviewed; Human; Language: English, Expanders - Apply related words, Search modes - Find any of my search terms, Interface - EBSCOhost Research Databases, Search Screen - Advanced Search, Database - CINAHL with Full Text

(Propriocep* OR (MH "Proprioception+") OR Kinesthe* OR (MH "Kinesthesis") OR sensorimotor OR sensory-motor OR "joint position sense" OR "joint position detection" OR "threshold to detect passive motion" OR "passive motion direction discrimination" OR "passive motion detection threshold" OR "threshold for motion detection" OR "threshold hunting" OR "detection threshold" OR "discrimination threshold" OR "ipsilateral matching" OR "contralateral matching" OR "joint angle error" OR "distance estimation error" OR "passive recognition" OR "direction accuracy" OR "active reproduction" OR "Joint reposition" OR "Active movement extent discrimination" OR "force sense" OR "force perception" OR "velocity sense") AND (S6 AND S7 AND S8 AND S9)

For peer review only

S6: “Anterior Cruciate Ligament” OR (MH "Anterior Cruciate Ligament") “Knee joint” OR (MH "Knee Joint+")

S7: Injur* OR (MH "Anterior Cruciate Ligament Injuries") OR Reconstruction OR (MH "Anterior Cruciate Ligament Reconstruction") OR Rupture OR Tear OR (MH "Rupture+") OR Conservative OR Deficiency OR “Joint instability” OR (MH "Joint Instability+"

S8: Propriocep* OR (MH "Proprioception+") OR Neuromuscular OR (MH "Neuromuscular Control") OR sensorimotor OR "sensory-motor" OR "Kinetic chain" OR (MH "Closed Kinetic Chain Exercises") OR (MH "Open Kinetic Chain Exercises") OR Coordination OR Balance OR (MH "Balance Training, Physical") OR (MH "Balance, Postural") OR Plyometric OR Vibration OR (MH "Vibration" OR Exercise* OR (MH "Exercise+") OR Intervention OR Training OR Rehabilitation OR Therapy OR (MH "Physical Therapy+") OR Treatment

S9: Propriocep* OR (MH "Proprioception+") OR Kinesthe* OR (MH "Kinesthesis") OR sensorimotor OR sensory-motor OR "joint position sense" OR "joint position detection" OR "threshold to detect passive motion" OR "passive motion direction discrimination" OR "passive motion detection threshold" OR "threshold for motion detection" OR "threshold hunting" OR "detection threshold" OR "discrimination threshold" OR "ipsilateral matching" OR "contralateral matching" OR "joint angle error" OR "distance estimation error" OR "passive recognition" OR "direction accuracy" OR "active reproduction" OR "Joint reposition" OR "force sense" OR "force perception" OR "velocity sense" OR "Active movement extent discrimination"

**Physical Education Index (ProQuest)**

((("Anterior Cruciate Ligament" OR "Knee joint") AND (Injur* OR Trauma OR Reconstruct* OR Ruptur* OR Tear OR Conservative OR Deficienc* OR "Joint instabilit*") AND (Propriocep* OR Kinesthes* OR neuromuscular OR sensorimotor OR sensory-motor OR "Kinetic chain" OR Coordination OR Balance OR Plyometric OR Vibration OR Exercise* OR Intervention OR Training OR Rehabilitation OR Therap* OR Treatment) AND (Propriocep* OR Kinesthes* OR sensorimotor OR sensory-motor OR "joint position sense" OR "joint position detection" OR "threshold to detect passive motion" OR "passive motion direction discrimination" OR "passive motion detection threshold" OR "threshold for motion detection" OR "threshold hunting" OR "detection threshold" OR "discrimination threshold" OR "ipsilateral matching" OR "contralateral matching" OR "joint angle error" OR "distance estimation error" OR "passive recognition" OR "direction accuracy" OR "active reproduction" OR "Joint reposition" OR "active movement extent discrimination" OR "force sense" OR "force perception" OR "velocity sense"))) AND at.exact("Article") AND la.exact("ENG") AND PEER(yes)

**PubMed**

(((((Anterior Cruciate Ligament[Text Word] OR "Anterior Cruciate Ligament"[Mesh] OR Knee joint[Text Word] OR "knee joint"[MeSH Terms]) AND "loattrfull text"[sb]) AND (((injury[All Fields]) OR Reconstruction[Text Word] OR "Anterior Cruciate Ligament Reconstruction"[Mesh] OR "Anterior Cruciate Ligament Injuries"[Mesh] OR Rupture[Text Word] OR Tear[Text Word] OR "Rupture"[Mesh] OR Conservative[Text Word] OR "Conservative Treatment"[Mesh] OR Deficiency[Text Word] OR Joint instability[Text Word] OR "Joint Instability"[Mesh]))) AND (((proprioception[All Fields]) OR "Proprioception"[Mesh] OR Neuromuscular[Text Word] OR sensorimotor[Text Word] OR sensory-motor[Text Word] OR Kinetic chain[Text Word] OR Coordination[Text Word] OR "Psychomotor Performance"[Mesh] OR Balance[Text Word] OR "Postural Balance"[Mesh] OR Plyometric[Text Word] OR "Plyometric

For peer review only

Exercise"[Mesh] OR ("exercise"[MeSH Terms] OR "exercises"[All Fields] OR "exercise therapy"[MeSH Terms]) OR "Exercise Therapy"[Mesh] OR Intervention[Text Word] OR Training[Text Word] OR "Resistance Training"[Mesh] OR Rehabilitation[Text Word] OR "Rehabilitation"[Mesh] OR Therapy[Text Word] OR Treatment[Text Word] OR "Treatment Outcome"[Mesh]))) AND (((proprioception[All Fields]) OR "Proprioception"[Mesh] OR ("kinesthesis"[MeSH Terms] OR "kinesthesis"[All Fields]) OR "Kinesthesis"[Mesh] OR joint position sense[Text Word] OR (("joints"[MeSH Terms] OR "joints"[All Fields] OR "joint"[All Fields]) AND position detection[Text Word]) OR threshold to detect passive motion[Text Word] OR (passive[All Fields] AND motion direction discrimination[Text Word]) OR (passive[All Fields] AND motion detection threshold[Text Word]) OR (threshold[All Fields] AND motion detection[Text Word]) OR threshold hunting[Text Word] OR detection threshold[Text Word] OR discrimination threshold[Text Word] OR (ipsilateral[All Fields] AND matching[Text Word]) OR contralateral matching[Text Word] OR joint angle error[Text Word] OR distance estimation error[Text Word] OR passive recognition[Text Word] OR direction accuracy[Text Word] OR active reproduction[Text Word] OR Joint reposition[Text Word] OR force sense[Text Word] OR force perception[Text Word] OR velocity sense[Text Word] OR (active[All Fields] AND ("movement"[MeSH Terms] OR "movement"[All Fields]) AND extent[All Fields] AND ("discrimination (psychology)"[MeSH Terms] OR ("discrimination"[All Fields] AND "(psychology)"[All Fields]) OR "discrimination (psychology)"[All Fields] OR "discrimination"[All Fields])) OR sensorimotor[Text Word] OR sensory-motor[Text Word]) AND "loattrfull text"[sb])) AND "loattrfull text"[sb] AND ("loattrfull text"[sb] AND English[lang]) AND English[lang]

**Scopus**

("Anterior Cruciate Ligament" OR "Knee joint" ) AND ( injur* OR trauma OR reconstruct* OR ruptur* OR tear OR conservative OR deficienc* OR "Joint instabilit*" ) AND ( propriocep* OR kinesthes* OR neuromuscular OR sensorimotor OR sensory-motor OR "Kinetic chain" OR coordination OR balance OR plyometric OR vibration OR exercise* OR intervention OR training OR rehabilitation OR therap* OR treatment ) AND ( propriocep* OR kinesthes* OR "joint position sense" OR "joint position detection" OR "threshold to detect passive motion" OR "passive motion direction discrimination" OR "passive motion detection threshold" OR "threshold for motion detection" OR "threshold hunting" OR "detection threshold" OR "discrimination threshold" OR "ipsilateral matching" OR "contralateral matching" OR "joint angle error" OR "distance estimation error" OR "passive recognition" OR "direction accuracy" OR "active reproduction" OR "Joint reposition" OR "active movement extent discrimination" OR "force sense" OR "force perception" OR "velocity sense" OR sensorimotor OR sensory-motor ) AND NOT INDEX (medline) AND (LIMIT-TO ( SRCTYPE , "j")) AND (LIMIT-TO ( DOCTYPE , "ar")) AND (LIMIT-TO ( SUBJAREA , "MEDI") OR LIMIT-TO ( SUBJAREA , "HEAL" ) OR LIMIT-TO ( SUBJAREA , "NEUR")) AND (LIMIT-TO ( LANGUAGE , "English")) AND ( LIMIT-TO (EXACTKEYWORD , "Human") OR LIMIT-TO (EXACTKEYWORD , "Article") OR LIMIT-TO (EXACTKEYWORD , "Male" ) OR LIMIT-TO ( EXACTKEYWORD , "Female") OR LIMIT-TO (EXACTKEYWORD , "Controlled Study") OR LIMIT-TO (EXACTKEYWORD , "Proprioception"))

**SPORTDiscus**

Limiters - Peer Reviewed; Language: English; Publication Type: Academic Journal; Document Type: Article, Expanders - Apply related words, Search modes - Find any of

For peer review only - http://bmjopen.bmj.com/site/about/guidelines.xhtml 

my search terms, Interface - EBSCOhost Research Databases, Search Screen - Advanced Search, Database - SPORTDiscus

(Propriocep* OR (DE "PROPRIOCEPTION") OR Kinesthe* OR sensorimotor OR sensory-motor OR "joint position sense" OR "joint position detection" OR "threshold to detect passive motion" OR "passive motion direction discrimination" OR "passive motion detection threshold" OR "threshold for motion detection" OR "threshold hunting" OR "detection threshold" OR "discrimination threshold" OR "ipsilateral matching" OR "contralateral matching" OR "joint angle error" OR "distance estimation error" OR "passive recognition" OR "direction accuracy" OR "active reproduction" OR "Joint reposition" OR "force sense" OR "force perception" OR "velocity sense" OR "active movement extent discrimination") AND (S1 AND S2 AND S3 AND S4)

S1: Anterior Cruciate Ligament OR (DE "CRUCIATE ligaments") OR (DE "ANTERIOR cruciate ligament") “Knee joint” OR (DE "KNEE"

S2: Injur* OR (DE "ANTERIOR cruciate ligament injuries") OR (DE "CRUCIATE ligament injuries) OR Reconstruction OR Rupture OR Tear OR Conservative OR Deficiency OR “Joint instabilit*”

S3: Propriocep* OR (DE "PROPRIOCEPTION") OR Neuromuscular OR sensorimotor OR sensory-motor OR Kinetic chain OR Coordination OR (DE "MOTOR ability") OR Balance OR Plyometric OR (DE "PLYOMETRICS) OR Vibration OR Exercise* OR Intervention OR Training OR Rehabilitation OR (DE "TREATMENT programs") OR (DE "REHABILITATION") OR Therap* OR Treatment OR (DE "KNEE injuries -- Treatment")

S4: Propriocep* OR (DE "PROPRIOCEPTION") OR Kinesthe* OR sensorimotor OR sensory-motor OR "joint position sense" OR "joint position detection" OR "threshold to detect passive motion" OR "passive motion direction discrimination" OR "passive motion detection threshold" OR "threshold for motion detection" OR "threshold hunting" OR "detection threshold" OR "discrimination threshold" OR "ipsilateral matching" OR "contralateral matching" OR "joint angle error" OR "distance estimation error" OR "passive recognition" OR "direction accuracy" OR "active reproduction" OR "Joint reposition" OR "force sense" OR "force perception" OR "velocity sense" OR "active movement extent discrimination"

For peer review only

**Online supplemental file 2.**

**Screening protocol – to screen eligible studies at the title, abstract, and full-text screening stages**

**Questions for all stages: title, abstract and full-text screening (follow stages 1-9):**

1) Is the study published in a scientific journal or published as a dissertation/thesis?
    a. No - exclude
    b. Yes or uncertain - go to step 2
2) Is the study written in English?
    a. No - exclude
    b. Yes or uncertain - go to step 3
3) Does the study deal with individuals who are 15 years of age and above?
    a. No - exclude
    b. Yes or uncertain - go to step 4
4) Does this study investigate individuals with an anterior cruciate ligament injury managed with conservative treatment or surgical reconstruction?
    a. No - exclude
    b. Yes or uncertain - go to step 5
5) Is the study a primary study (i.e. no letter to the editor, book reviews, published study designs/trial protocols, commentaries, editorials, interviews, newspaper articles, patient education handouts, consensus statements or clinical practice guidelines)?
    a. No - exclude
    b. Yes or uncertain - go to step 6
6) Does the intervention group in the study undergo neuromuscular training rehabilitation?
    a. No - exclude
    b. Yes or uncertain - go to step 7
7) Is the comparator/control group in the study include any of the following: any other therapy, conventional training, usual care, placebo or sham therapy?
    a. No - exclude
    b. Yes or uncertain - go to step 8
8) Does the study evaluate knee proprioception using a specific test (joint position sense, joint position detection, threshold to detect passive motion, passive motion direction discrimination, passive motion detection threshold, threshold for motion detection, threshold hunting, detection threshold, discrimination threshold, ipsilateral matching, contralateral matching, joint angle error, distance estimation error, passive recognition, direction accuracy, active reproduction, active movement extent discrimination, force sense, force perception, velocity sense or any other related tests)- before and after the intervention?
    a. No - exclude
    b. Yes or uncertain - go to step 9
9) Does the study report (objective) focal measures of knee proprioception for any of the specific tests mentioned in point 8?
    a. No - exclude
    b. Yes or uncertain - choose one of the following options:
        i. Title and abstract screening stage - include
        ii. Full-text screening stage - follow step 10-11

**Additional questions for full-text stage only:**

10) Does the study use at least one (appropriate) statistical test to compare the intervention and comparator/control groups for knee proprioception?

For peer review only

a. No - exclude
b. Yes or uncertain - go to step 11

11) Are the points 1-10 scored as “yes or uncertain”

a. If all “yes” - include
b. If any “uncertain” - discuss with another reviewer to come to an agreement whether to include the study or not

For peer review only

**Online supplemental file 3.**

**Data extraction template**

| Publication details | Study citation, clinical trial registration, and published study protocol if available |
|---|---|
| Aim of the study | Primary and/or secondary aims relevant for the review. |
| Eligibility criteria | Inclusion and exclusion criteria for participants |
| Randomized/controlled clinical trial | Randomization method? |
| Participant allocation | Concealed or not? |
| Number of participants identified | Identified, included and excluded? |
| All participants accounted for entire study | Yes or no? |
| Experimental group | Experimental intervention (type of neuromuscular rehabilitation training) given. |
| Comparator group | Comparator intervention given. |
| Assessment method, equipment used, and outcome measure(s) of interest | Those related to knee-specific proprioception senses. |
| Method(s) used for measuring the outcome(s) appropriate? | Authors quoted any data on reliability and validity based on the previous literature or their own data? |
| Multiple measurements of the same outcome measure within the outcome domain? | Different methods measuring same proprioception sense and different time points? |
| Participant characteristics | Anthropometric, demographic, physical activity and function levels, and any other relevant information to ACL injury and/or surgery. |
| Groups were similar at baseline | Anthropometrics, demographics, outcome measure(s) of interest, and any other prognostic indicators. |
| Blinding | Participants, investigators, therapists/clinicians/those delivering the interventions, and outcome assessors. |
| The outcome measure of interest was obtained from more than 85% of the participants initially allocated to groups | For continuous outcomes, availability of data from 95% (or possibly 90%) of the participants would often be sufficient. |
| If data were missing, how they were handled | ‘Last observation carried forward’, ‘baseline observation carried forward’ or any other method? |
| Analyses preplanned | Information available from Registered trial protocol or any other relevant information available? |
| Between-group statistical comparisons | Statistical analysis for measurement of proprioception was done by “intention to treat” or “per-protocol” analysis? Multiple analysis of data? Corrected for multiple analysis of data? Selective reporting of analysis? |
| Results | Selective reporting of a particular outcome measurement? |
| Conclusion | Authors’ conclusions |

For peer review only

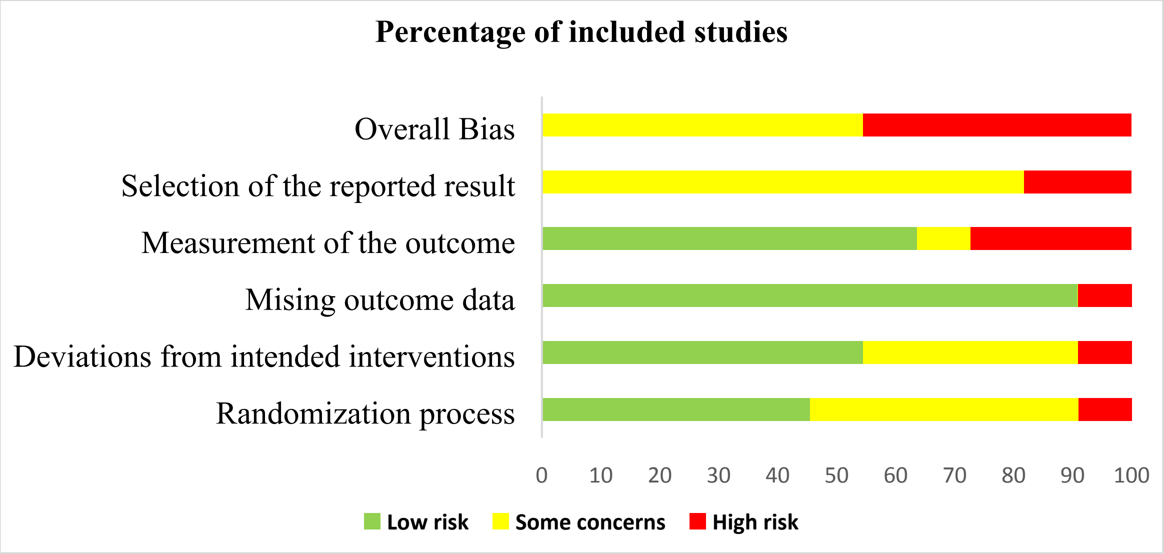


**Online supplemental figure 1.** Risk of bias assessment in each of the five domains and overall bias. Percentage of studies showing low risk of bias, some concerns and high risk of bias.

Note: For studies having more than one relevant outcome, each outcome is considered separately for risk of bias assessment.

peer review only

| Study or Subgroup | Neuromuscular training Mean | SD | Total | Comparator Mean | SD | Total | Weight | Mean Difference IV, Random, 95% CI |
|---|---|---|---|---|---|---|---|---|
| Baltaci JPS 1 | 2.4 | 61 | 15 | 0.5 | 23.6 | 15 | | Not estimable |
| Baltaci JPS 2 | 47 | 114.9 | 15 | 13.7 | 38.5 | 15 | | Not estimable |
| Cho JPS 15 | 1.95 | 1.04 | 14 | 1.81 | 1.2 | 14 | 8.9% | 0.14 [-0.69, 0.97] |
| Cho JPS 45 | 0.64 | 1.71 | 14 | 1.51 | 1.01 | 14 | 8.2% | -0.87 [-1.91, 0.17] |
| Fu JPS 30 | 4.43 | 3.09 | 19 | 5.25 | 2.84 | 20 | 5.7% | -0.82 [-2.69, 1.05] |
| Fu JPS 60 | 4.07 | 2.17 | 19 | 4.77 | 2.93 | 20 | 6.4% | -0.70 [-2.31, 0.91] |
| Kaya JPS 15 | 15.44 | 1.74 | 17 | 16.95 | 3.13 | 15 | 5.9% | -1.51 [-3.30, 0.28] |
| Kaya JPS 45 | 45.63 | 2.94 | 17 | 47.32 | 6.05 | 15 | 2.8% | -1.69 [-5.06, 1.68] |
| Kaya JPS 75 | 74.55 | 1.54 | 17 | 75.85 | 3.77 | 15 | 5.2% | -1.30 [-3.34, 0.74] |
| Moezy JPS 30 | 1.93 | 2.93 | 10 | 0.27 | 1.58 | 10 | 5.2% | 1.66 [-0.40, 3.72] |
| Moezy JPS 60 | 3.47 | 1.88 | 10 | 0.44 | 1.49 | 10 | 6.8% | 3.03 [1.54, 4.52] |
| Shen JPS 20 | 4.47 | 1.22 | 10 | 5.87 | 1.48 | 10 | 7.8% | -1.40 [-2.59, -0.21] |
| Shen JPS 50 | 4.37 | 1.07 | 10 | 5.73 | 1.18 | 10 | 8.4% | -1.36 [-2.35, -0.37] |
| Shen JPS 80 | 4.49 | 0.92 | 10 | 5.77 | 1.39 | 10 | 8.2% | -1.28 [-2.31, -0.25] |
| Zult JPS 15 | 0 | 3 | 22 | -1 | 4 | 21 | 5.0% | 1.00 [-1.12, 3.12] |
| Zult JPS 30 | 2 | 3 | 22 | 0 | 4 | 21 | 5.0% | 2.00 [-0.12, 4.12] |
| Zult JPS 45 | -2 | 4 | 22 | -1 | 4 | 21 | 4.4% | -1.00 [-3.39, 1.39] |
| Zult JPS 60 | 0 | 3 | 22 | 1 | 3 | 21 | 5.9% | -1.00 [-2.79, 0.79] |
| **Total (95% CI)** | | | **255** | | | **247** | **100.0%** | **-0.34 [-1.01, 0.32]** |

Heterogeneity: Tau² = 1.13; Chi² = 46.08, df = 15 (P < 0.0001); I² = 67%
Test for overall effect: Z = 1.01 (P = 0.31)

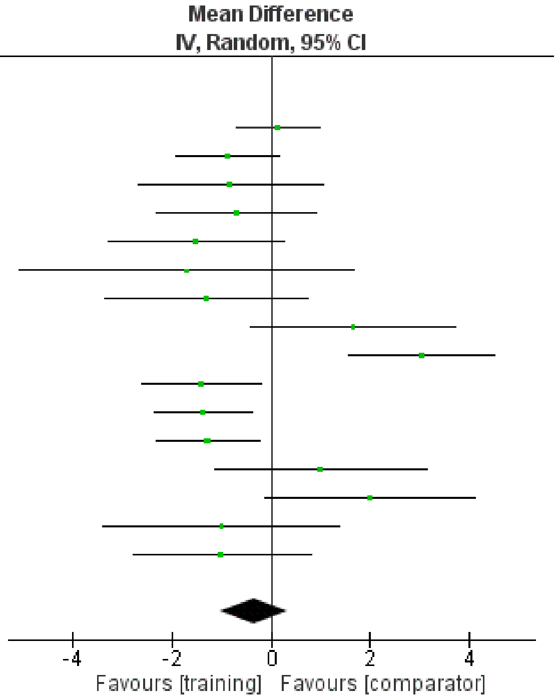


**Online supplementary figure 2. A meta-analysis comparing mean differences between the neuromuscular training and comparator groups for knee joint position sense.**

| Study or Subgroup | Neuromuscular training Mean | SD | Total | Comparator Mean | SD | Total | Weight | Mean Difference IV, Random, 95% CI |
|---|---|---|---|---|---|---|---|---|
| Beynnon TTDPM | 1.35 | 0.87 | 19 | 1.26 | 0.7 | 17 | 21.0% | 0.09 [-0.42, 0.60] |
| Risberg TTDPM 15 | 1.2 | 0.76 | 23 | 1.22 | 0.52 | 24 | 22.2% | -0.02 [-0.39, 0.35] |
| Shen TTDPM 20 | 2.26 | 0.89 | 10 | 3.6 | 0.87 | 10 | 18.2% | -1.34 [-2.11, -0.57] |
| Shen TTDPM 50 | 2.07 | 0.75 | 10 | 3.47 | 0.74 | 10 | 19.5% | -1.40 [-2.05, -0.75] |
| Shen TTDPM 80 | 2.27 | 0.7 | 10 | 3.56 | 0.9 | 10 | 19.0% | -1.29 [-2.00, -0.58] |
| **Total (95% CI)** | | | **72** | | | **71** | **100.0%** | **-0.75 [-1.43, -0.07]** |

Heterogeneity: $Tau^2 = 0.50$; $Chi^2 = 27.98$, df = 4 ($P < 0.0001$); $I^2 = 86\%$
Test for overall effect: $Z = 2.16$ ($P = 0.03$)

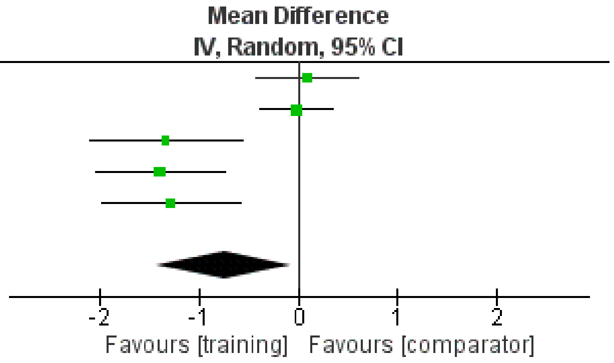


**Online supplementary figure 3. A meta-analysis comparing mean differences between the neuromuscular training and comparator groups for knee joint threshold to detect passive motion.**

For peer review only

| Study or Subgroup | Neuromuscular training Mean | SD | Total | Comparator Mean | SD | Total | Weight | Mean Difference IV, Random, 95% CI |
|---|---|---|---|---|---|---|---|---|
| Zult QFC Con 60 deg/s | -1 | 6 | 22 | -7 | 11 | 21 | 18.8% | 6.00 [0.67, 11.33] |
| Zult QFC Ecc 60 deg/s | -5 | 5 | 22 | -4 | 5 | 21 | 34.7% | -1.00 [-3.99, 1.99] |
| Zult QFC Isometric | 0 | 1 | 22 | -1 | 4 | 21 | 46.5% | 1.00 [-0.76, 2.76] |
| **Total (95% CI)** | | | **66** | | | **63** | **100.0%** | **1.25 [-1.58, 4.07]** |

Heterogeneity: Tau² = 3.66; Chi² = 5.08, df = 2 (P = 0.08); I² = 61%
Test for overall effect: Z = 0.86 (P = 0.39)

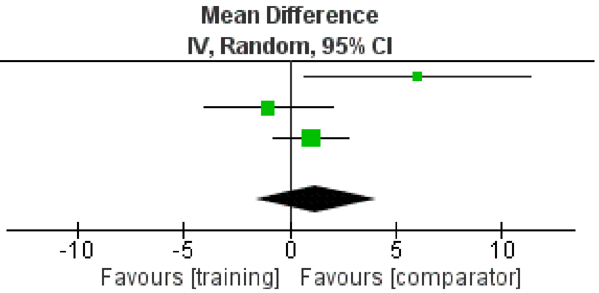


**Online supplementary figure 4. A meta-analysis comparing mean differences between the neuromuscular training and comparator groups for knee joint quadriceps force control.**

For peer review only

# PRISMA 2009 Checklist

| Section/topic | # | Checklist item | Reported on page # |
|---|---|---|---|
| **TITLE** | | | |
| Title | 1 | Identify the report as a systematic review, meta-analysis, or both. | Page 1 |
| **ABSTRACT** | | | |
| Structured summary | 2 | Provide a structured summary including, as applicable: background; objectives; data sources; study eligibility criteria, participants, and interventions; study appraisal and synthesis methods; results; limitations; conclusions and implications of key findings; systematic review registration number. | Page 2 |
| **INTRODUCTION** | | | |
| Rationale | 3 | Describe the rationale for the review in the context of what is already known. | Pages 5-7 |
| Objectives | 4 | Provide an explicit statement of questions being addressed with reference to participants, interventions, comparisons, outcomes, and study design (PICOS). | Page 7 |
| **METHODS** | | | |
| Protocol and registration | 5 | Indicate if a review protocol exists, if and where it can be accessed (e.g., Web address), and, if available, provide registration information including registration number. | PROSPERO registration number CRD42018107349 |
| Eligibility criteria | 6 | Specify study characteristics (e.g., PICOS, length of follow-up) and report characteristics (e.g., years considered, language, publication status) used as criteria for eligibility, giving rationale. | Pages 7-8 |
| Information sources | 7 | Describe all information sources (e.g., databases with dates of coverage, contact with study authors to identify additional studies) in the search and date last searched. | Page 8 |
| Search | 8 | Present full electronic search strategy for at least one database, including any limits used, such that it could be repeated. | Online supplemental file 1 (pages 43-46) |
| Study selection | 9 | State the process for selecting studies (i.e., screening, eligibility, included in systematic review, and, if applicable, included in the meta-analysis). | Pages 9-10, online supplemental file 2 (page 47-48) |
| Data collection process | 10 | Describe method of data extraction from reports (e.g., piloted forms, independently, in duplicate) and any processes for obtaining and confirming data from investigators. | Page 9, Online supplemental file 3. Data extraction template |

For peer review only

# PRISMA 2009 Checklist

| | | | |
|---|---|---|---|
| | | | (page 49) |
| Data items | 11 | List and define all variables for which data were sought (e.g., PICOS, funding sources) and any assumptions and simplifications made. | Online supplemental file 3 (page 49) |
| Risk of bias in individual studies | 12 | Describe methods used for assessing risk of bias of individual studies (including specification of whether this was done at the study or outcome level), and how this information is to be used in any data synthesis. | Pages 9-10 |
| Summary measures | 13 | State the principal summary measures (e.g., risk ratio, difference in means). | Pages 9-10 |
| Synthesis of results | 14 | Describe the methods of handling data and combining results of studies, if done, including measures of consistency (e.g., $I^2$) for each meta-analysis. | Pages 9-10 |

Page 1 of 2

| Section/topic | # | Checklist item | Reported on page # |
|---|---|---|---|
| Risk of bias across studies | 15 | Specify any assessment of risk of bias that may affect the cumulative evidence (e.g., publication bias, selective reporting within studies). | Pages 9-10 |
| Additional analyses | 16 | Describe methods of additional analyses (e.g., sensitivity or subgroup analyses, meta-regression), if done, indicating which were pre-specified. | NA |
| **RESULTS** | | | |
| Study selection | 17 | Give numbers of studies screened, assessed for eligibility, and included in the review, with reasons for exclusions at each stage, ideally with a flow diagram. | Figure 1 (page 42) |
| Study characteristics | 18 | For each study, present characteristics for which data were extracted (e.g., study size, PICOS, follow-up period) and provide the citations. | Pages 10-11, Table 1 (pages 31-36) |
| Risk of bias within studies | 19 | Present data on risk of bias of each study and, if available, any outcome level assessment (see item 12). | Pages 11-12, Table 2 (page 37) |
| Results of individual studies | 20 | For all outcomes considered (benefits or harms), present, for each study: (a) simple summary data for each intervention group (b) effect estimates and confidence intervals, ideally with a forest plot. | Table 1 (pages 31-36), online |

For peer review only

# PRISMA 2009 Checklist

| | | | |
|---|---|---|---|
| | | | supplemental figures 2-4 |
| Synthesis of results | 21 | Present results of each meta-analysis done, including confidence intervals and measures of consistency. | online supplemental figures 2-4 |
| Risk of bias across studies | 22 | Present results of any assessment of risk of bias across studies (see Item 15). | Pages 11-12, Table 2 (page 37), Table 3 (page 38), Table 4 (pages 39-41) |
| Additional analysis | 23 | Give results of additional analyses, if done (e.g., sensitivity or subgroup analyses, meta-regression [see Item 16]). | NA |
| **DISCUSSION** | | | |
| Summary of evidence | 24 | Summarize the main findings including the strength of evidence for each main outcome; consider their relevance to key groups (e.g., healthcare providers, users, and policy makers). | Pages 15-16 |
| Limitations | 25 | Discuss limitations at study and outcome level (e.g., risk of bias), and at review-level (e.g., incomplete retrieval of identified research, reporting bias). | Page 17, Pages 20-21 |
| Conclusions | 26 | Provide a general interpretation of the results in the context of other evidence, and implications for future research. | Page 21 |
| **FUNDING** | | | |
| Funding | 27 | Describe sources of funding for the systematic review and other support (e.g., supply of data); role of funders for the systematic review. | Page 22 |

*From:* Moher D, Liberati A, Tetzlaff J, Altman DG, The PRISMA Group (2009). Preferred Reporting Items for Systematic Reviews and Meta-Analyses: The PRISMA Statement. PLoS Med 6(7): e1000097. doi:10.1371/journal.pmed1000097

For more information, visit: **www.prisma-statement.org**.



For peer review only - http://bmjopen.bmj.com/site/about/guidelines.xhtml

# Effects of neuromuscular training on knee proprioception in individuals with anterior cruciate ligament injury - A systematic review and GRADE evidence synthesis

| | |
|---|---|
| Journal: | *BMJ Open* |
| Manuscript ID | bmjopen-2021-049226.R1 |
| Article Type: | Original research |
| Date Submitted by the Author: | 25-Mar-2021 |
| Complete List of Authors: | Arumugam, Ashokan; University of Sharjah College of Health Sciences, Department of Physiotherapy<br>Björklund, Martin; Umea University, Department of Community Medicine and Rehabilitation – Physiotherapy Section; University of Gavle Department of Occupational Health and Psychology, Department of Occupational Health Sciences and Psychology, Centre for Musculoskeletal Research<br>Mikko, Sanna; Umea University, Department of Community Medicine and Rehabilitation – Physiotherapy Section<br>Häger, Charlotte ; Umea University, 1Department of Community Medicine and Rehabilitation – Physiotherapy Section |
| <b>Primary Subject Heading</b>: | Sports and exercise medicine |
| Secondary Subject Heading: | Rehabilitation medicine |
| Keywords: | Knee < ORTHOPAEDIC & TRAUMA SURGERY, Orthopaedic sports trauma < ORTHOPAEDIC & TRAUMA SURGERY, REHABILITATION MEDICINE, SPORTS MEDICINE |
| | |

SCHOLARONE™
Manuscripts

BMJ

*I, the Submitting Author has the right to grant and does grant on behalf of all authors of the Work (as defined in the below author licence), an exclusive licence and/or a non-exclusive licence for contributions from authors who are: i) UK Crown employees; ii) where BMJ has agreed a CC-BY licence shall apply, and/or iii) in accordance with the terms applicable for US Federal Government officers or employees acting as part of their official duties; on a worldwide, perpetual, irrevocable, royalty-free basis to BMJ Publishing Group Ltd ("BMJ") its licensees and where the relevant Journal is co-owned by BMJ to the co-owners of the Journal, to publish the Work in this journal and any other BMJ products and to exploit all rights, as set out in our licence.*

*The Submitting Author accepts and understands that any supply made under these terms is made by BMJ to the Submitting Author unless you are acting as an employee on behalf of your employer or a postgraduate student of an affiliated institution which is paying any applicable article publishing charge ("APC") for Open Access articles. Where the Submitting Author wishes to make the Work available on an Open Access basis (and intends to pay the relevant APC), the terms of reuse of such Open Access shall be governed by a Creative Commons licence – details of these licences and which Creative Commons licence will apply to this Work are set out in our licence referred to above.*

*Other than as permitted in any relevant BMJ Author's Self Archiving Policies, I confirm this Work has not been accepted for publication elsewhere, is not being considered for publication elsewhere and does not duplicate material already published. I confirm all authors consent to publication of this Work and authorise the granting of this licence.*

For peer review only

# Effects of neuromuscular training on knee proprioception in individuals with anterior cruciate ligament injury - A systematic review and GRADE evidence synthesis

**Authors:** Ashokan Arumugam (M.P.T., Ph.D.)[1], Martin Björklund (R.P.T., Ph.D.)[2, 3], Sanna Mikko (B.Sc.)[2], Charlotte K. Häger (R.P.T., Ph.D.)[2]

**Author Affiliations:**

[1]Department of Physiotherapy, College of Health Sciences, University of Sharjah, P.O. Box: 27272, Sharjah, United Arab Emirates;
[2]Department of Community Medicine and Rehabilitation – Physiotherapy Section, Umeå, University, SE-901 87, Umeå, Sweden;
[3]Department of Occupational Health Sciences and Psychology, Centre for Musculoskeletal Research, University of Gävle, Gävle, Sweden.

**Corresponding author:** Charlotte K. Häger

**Address for Correspondence:**

| | |
|---|---|
| Name | Charlotte K. Häger, R.P.T., Ph.D. |
| Designation | Professor |
| Department | Community Medicine and Rehabilitation – Physiotherapy Section |
| Institution | Umeå University |
| Country | Sweden |
| Mobile | +46 90 786 9275 |
| Email | charlotte.hager@umu.se |

**Manuscript Type:** A systematic review

**Word count:** 5135 (excluding abstract, tables, contributions, funding acknowledgements and references)

**Ethical approval:** Not required.

**Funding sources:** The Swedish Research Council (2017-00892); Region Västerbotten (ALF 7003575; Strategic funding VLL-358901; Project.No 7002795); The Swedish Research Council for Sports Science (CIF P2019 0068); and King Gustaf V and Queen Victoria's Foundation of Freemasons 2019 (Häger).

**Conflicts of interest:** None declared.

**Ethical approval:** Not required.

**Competing interests:** None declared.



For peer review only - http://bmjopen.bmj.com/site/about/guidelines.xhtml

# Effects of neuromuscular training on knee proprioception in individuals with anterior cruciate ligament injury - A systematic review and GRADE evidence synthesis


## Abstract

**Objective**

To systematically review and summarize the evidence for the effects of neuromuscular training compared to any other therapy (conventional training/sham) on knee proprioception following anterior cruciate ligament (ACL) injury.

**Design** Systematic Review

**Data Sources**

PubMed, CINAHL, SPORTDiscus, AMED, Scopus, and Physical Education Index were searched from inception to February 2020.

**Eligibility Criteria**

Randomized controlled trials (RCTs) and controlled clinical trials investigating the effects of neuromuscular training on knee-specific proprioception tests following a unilateral ACL injury were included.

**Data extraction and synthesis**

Two reviewers independently screened and extracted data and assessed risk of bias of the eligible studies using the Cochrane risk of bias 2 tool. Overall certainty in evidence was determined using the GRADE tool.

**Results** Of 2706 articles retrieved, only nine RCTs, comprising 327 individuals with an ACL reconstruction (ACLR), met the inclusion criteria. Neuromuscular training interventions varied across studies: whole body vibration therapy, Nintendo-Wii-Fit training, balance training, sport-specific exercises, backward walking, etc. Outcome measures included joint position sense (JPS; n=7), thresholds to detect passive motion (TTDPM; n=3), or quadriceps force control (QFC; n=1). Overall, between-group mean differences indicated inconsistent


For peer review only

findings with an increase or decrease of errors associated with JPS by ≤2º, TTDPM by ≤1.5º, and QFC by ≤6 Nm in the ACLR knee following neuromuscular training. Owing to serious concerns with three or more GRADE domains (risk of bias, inconsistency, indirectness, or imprecision associated with the findings) for each outcome of interest across studies, the certainty of evidence was very low.

**Conclusions** The heterogeneity of interventions, methodological limitations, inconsistency of effects (on JPS/TTDPM/QFC) preclude recommendation of one optimal neuromuscular training intervention for improving proprioception following ACL injury in clinical practice. There is a need for methodologically-robust RCTs with homogenous populations with ACL injury (managed conservatively or with reconstruction), novel/well-designed neuromuscular training, and valid proprioception assessments, which also seem to be lacking.

**PROSPERO registration number**

CRD42018107349

**Key words:** Joint position sense, threshold to detect passive motion, ACL, sensorimotor training, literature review, neuroplasticity

For peer review only

**Strengths and limitations of the study**

- A systematic review of neuromuscular training on knee proprioception following the Preferred Reporting Items for Systematic reviews and Meta-Analyses guidelines, using a broad search in six electronic databases.
- The risk of bias associated with the outcomes of interest (knee proprioception measures) in the included RCTs were assessed using the updated Cochrane risk of bias 2 tool.
- The overall certainty of evidence for the effects of neuromuscular training on knee joint position sense, threshold to detect passive motion, and quadriceps force control following ACL injury/reconstruction was ascertained using the GRADE tool.
- Only RCTs published in English were included.
- A meta-analysis was precluded because of clinical heterogeneity of interventions and outcome measures.

For peer review only

## Introduction

Anterior cruciate ligament (ACL) injury is a common musculoskeletal injury [1,2] accounting for an annual incidence rate of 68.6/100,000 person-years in the United States.[3] ACL injury is most prevalent in young athletes (14-18 years for females and 19-25 years for males).[3] The injury occurs more often during competition rather than training, with ~70% or more of the injuries representing noncontact mechanisms[4,5] such as landing from a jump, sudden deceleration and/or while cutting.[6] Thus, the injury mechanisms are related to neuromotor control, amongst other factors, of the individual. ACL injury is predominantly treated by surgical reconstruction,[3] and followed by a long period of rehabilitation and yet many individuals do not return to pre-injury levels of activity[7] which challenges the efficacy of existing preventative and rehabilitative strategies.

Individuals with an ACL injury present with a decreased number of proprioceptive mechanoreceptors (Pacinian capsules, Ruffini nerve endings and Golgi tendon organs)[8,9] which might alter somatosensory input to the central nervous system (CNS)[9] leading to decreased knee proprioception. Disturbed proprioception might also be caused by acute inflammation and pain, and the capsule and surrounding ligaments getting affected following instability.[10,11] Although there has been a debate regarding the effects of ACL injury on different knee proprioception tests,[2,12] our recent systematic review[13] suggests that knee JPS tests have sufficient validity in discriminating ACL-injured knees from asymptomatic knees (accepted). When compared to non-injured controls, individuals with ACL injury demonstrate altered movement strategies,[4,14] quadriceps muscle weakness,[15] and onset and progression of osteoarthrosis.[6,16] Due to the potential serious consequences of the injury, much attention and clinical efforts have been dedicated to preventative and rehabilitative strategies for ACL injury,[11] including various neuromuscular training (NT) methods believed to improve the proprioceptive ability.

For peer review only

Even if proprioceptive deficits could affect neuromotor control, the rationale, mechanisms, and plausibility for improving proprioception by training need to be verified. In the context of neuroplasticity, functional magnetic resonance imaging has revealed that individuals with ACL-deficient knees demonstrate less activation in several sensorimotor cortical areas and increased activation in pre-supplementary motor areas, posterior secondary somatosensory area, and posterior inferior temporal gyrus compared to controls with asymptomatic knees during a knee flexion-extension task.[1] It seems individuals with ACL reconstruction adapt a visual-sensory-motor strategy instead of a normal sensory-motor strategy owing to aberrant sensory feedback following ACL injury.[17] Nevertheless, neuroplastic reorganization ensues where other potential sensory sources are used to organize the movement or regulate neuromotor control, particularly in (sporting) tasks with higher complexity. Therefore, ACL injuries might be regarded as a neuromotor control dysfunction rather than a simple peripheral musculoskeletal injury.[11,18] It is yet unclear though whether neuromuscular training (NT) can improve proprioception after an ACL injury[11,19] and the neurophysiological mechanisms underpinning such interventions need further substantiation.

To date, there is no consensus on the most effective rehabilitation programs for ACL injury, and the prevalence of reinjury after returning to sport is up to 30%.[18] Owing to the neuroplastic changes and possibly altered proprioception following an ACL injury, NT has received much attention to enhance dynamic joint stability and relearn movement patterns and skills.[20] In this context, both NT and sensorimotor training terms have been used. NT is defined as "…training enhancing unconscious motor responses by stimulating both afferent signals and central mechanisms responsible for dynamic joint control" [20] and sensorimotor training aims to improve "…function of the CNS in regulating movement in order to reach proper firing patterns for maintaining joint stability…".[21] Active knee motion will in any case

For peer review only

stimulate proprioceptors, which in turn would alter the demands on the CNS,[10,19]. Henceforth we will use the term NT in this review.

There are different ways to challenge proprioception, for example: vibration may be used to alter afferent input from muscle spindles; an unstable surface can challenge input from the ankle; vision can be occluded or head position can be changed to disturb visual- and vestibular information,[10] or focus can be shifted to influence cognitive processing sources.[18] Due to a putative visual-sensory-motor strategy following ACL injury, a modified visual feedback training might decrease visual reliance and improve sensory-motor function.[18] Most studies exploring the effects of NT on proprioception combine different exercises and various outcome measures which precludes isolating the effects of a proprioception-specific exercise.[22] Therefore, this study aimed at systematically reviewing and summarizing the evidence for the effects of NT compared to comparator/control interventions on proprioception measured by knee-specific proprioception tests in individuals with anterior cruciate ligament injury or reconstruction.

## Methods

We adhered to the Preferred Reporting Items for Systematic review and Meta-Analysis (PRISMA) checklist[23] and the reporting guidelines for Synthesis Without Meta-analysis (SWiM) in systematic reviews.[24] The protocol was registered in PROSPERO (CRD42018107349). A list of acronyms used in the review is summarized in Box 1.

### Eligibility criteria

The structure of PICOS[25] was used to frame the following criteria:

1. **Participants:** Individuals aged over 15 years of age (both sexes) with a history of a unilateral ACL rupture, managed conservatively or surgically reconstructed, with or

without concomitant meniscus and/or collateral ligament injuries on the injured leg, without any other lower extremity injuries/surgeries that would confound the outcomes of rehabilitation training;

2. **Intervention:** Specific neuromuscular training, closed or open kinetic chain exercises, balance training, joint repositioning training, joint force sense training, co-ordination training, plyometric training, whole body vibration, virtual gaming training, an accelerated rehabilitation protocol or any other training programs focusing on improving the lower limb neuromuscular control and knee proprioception;
3. **Comparator:** Any other therapy, conventional training, usual care, placebo or sham therapy;
4. **Outcome measures:** Knee-specific proprioception tests targeting joint position sense (JPS), kinesthesia (threshold to detect passive motion [TTDPM]), force sense/perception, active movement extent discrimination, velocity sense, or psychophysical threshold methods;[13] they can be performed actively and/or passively with or without visual input in weight bearing or non-weight bearing positions;[10]
5. **Study design:** randomized controlled trials (RCTs) or controlled clinical trials.

## Data Sources and Searches

Database-specific search terms (e.g. MeSH) were combined using Boolean operators (“AND” and “OR”) under three conceptual domains: participants, interventions and outcomes. Six electronic databases were searched from their inception to 12 February 2020: PubMed, Cumulative Index to Nursing & Allied Health Literature (CINAHL via EBSCOhost), SPORTDiscus (via EBSCOhost), the Allied and Complementary Medicine

For peer review only

Database (AMED via EBSCOhost), Scopus, and Physical Education Index (via Proquest) (Online supplemental file 1).

**Study Selection**

One reviewer (SM) imported all titles and abstracts retrieved from the databases into EndNote X8. Two reviewers (AA and SM) independently checked titles, abstracts, and/or full text by following a screening questionnaire (online supplemental file 2). Any disagreements in inclusion of articles were adjudicated by two other reviewers (CH and MB) until consensus was reached. A manual search of the reference lists of included articles was performed.

**Data Extraction**

Data were extracted by one reviewer (SM) and verified by another reviewer (AA) using a customized data extraction sheet (online supplemental file 3). If any data were missing, the corresponding authors were contacted via email.

**Quality Assessment**

The risk of bias for each outcome of interest in the included studies was evaluated using the Cochrane ROB 2.[26] The tool has five domains: 1) randomization (number of signaling questions (n=3), 2) deviations from intended interventions (n=7), 3) missing outcome data (n=5), 4) measurement of the outcomes (n=5), and 5) selection of the reported results (n=3). Each signaling question can be answered as 1) yes, 2) probably yes, 3) probably no, 4) no, and 5) no information. Responses to the questions provide the basis for judgement of the risk of bias at each domain level using a tool-specific algorithm resulting in one out of three possible judgements: 1) low risk of bias, 2) some concerns, or 3) high risk of bias. An overall risk of bias score for each outcome in a study can be low (with a low risk of bias for all

For peer review only

domains), some concerns (if some concerns prevail in at least one domain without a high risk of bias for any domain) or high (if a high risk of bias underpins at least one domain or some concerns remain in multiple domains, defining multiple as more than two).

**Evidence Synthesis**

The overall evidence level in this review was determined using the Grading of Recommendations, Assessment, Development and Evaluation (GRADE) tool considering the following five domains: 1. risk of bias: high risk, some concerns, or low risk associated with knee proprioception measures based on the Cochrane ROB 2 tool; 2. Inconsistency of findings: similar or conflicting direction of effect, effect estimates and overlap of confidence intervals for knee proprioception measures from different studies; 3. indirectness of evidence: appropriateness of participants, interventions, and outcomes used to answer the review question; 4. imprecision of results: the length of 95% confidence intervals of effect estimates and overall sample (number of participants) from which effect estimates are derived; and other domains: e.g. publication bias if applicable.[27] The overall evidence was rated as very low, low, moderate or high.

A meta-analysis was precluded owing to clinical heterogeneity of interventions and outcome measurements (JPS, TTDPM and QFC). For instance, despite seven studies targeting JPS, a meta-analysis was not appropriate because at most two studies used the same method (active-active,[28,29] passive-passive[30,31] or passive-active[32,33]) but the starting and target angles and the number of trials per each angle varied between these proprioception tests in the included studies. Further, the neuromusuclar training interventions, targeting JPS, widely varied between studies[28-34]: closed kinetic chain exercises on a balance pad,[34] whole-body vibration therapy (WBVT),[29,30] motor control exercises for the lower limbs,[32] backward walking on a treadmill,[31] Nintendo Wii Fit training,[28] and cross-education of strength training of the non-

For peer review only

injured leg along with standard rehabilitation.[33] Further, in addition to inconsistent findings among the studies, a significant statistical heterogeneity ($I^2$>60%) in a random-effects meta-analysis was evident. Although meta-analyses were excluded, the Review Manager 5.3 software (the Cochrane Collaboration) was used to calculate between-group mean differences (effect sizes) and their 95% confidence intervals for summarizing the findings for each outcome of interest in Table 1.

**Patient and public involvement**

Neither patients nor public were involved.

**Results**

**Search Results**

Electronic databases search led to a total of 2706 articles (excluding duplicates: 2162). After title and abstract screening, 22 articles were shortlisted for full-text screening and subsequently nine articles met the inclusion criteria (Figure 1). Thirteen articles were excluded owing to the following reasons: not an RCT (n= 1),[35] no knee-specific proprioception tests (n= 6),[36-41] participants were without an ACL injury (n = 1),[42] knee proprioception data were missing and the corresponding author did not respond to our emails (n = 1),[43] a comparison between different surgical intervention groups with same rehabilitation program (n = 2),[44,45] and lack of a neuromuscular rehabilitation training program (n = 2).[46,47] No additional relevant studies were identified through manual search of bibliographic references.

**Study Design and Participants**

For peer review only

All the nine studies included were RCTs with a total of 386 participants and two studies had their trial pre-registered in a clinical trial registry.[31,33] All participants had undergone an ACL reconstruction with a bone-patellar-tendon-bone or a hamstring graft (Table 1).

**Quality Assessment**

The agreement (Cohen's kappa) of responses to the signaling questions between the two reviewers (AA and MB) was substantial ($0.69 \pm 0.047$, $p < 0.001$). Disagreements were discussed and resolved by the two reviewers. Online supplemental figure 1 shows the percentage of studies judged as low risk, some concerns and high risk of bias in the five domains, and Table 2 shows domain judgements of each study. The overall risk of bias judgement showed that four of the included studies had a high risk of bias,[28,29,32,34] four had some concerns,[30,31,48,49] and one study[33] had a high risk of bias for JPS and some concerns for quadriceps force control (QFC). The domain that most consistently showed risk of bias across studies was bias in selection of the reported results (Online supplemental figure 1 and Table 2). The most common reason was the absence of information regarding pre-specified plan of analyses. None of the included studies reported trial protocol publication and only two[31,33] reported trial registration. Furthermore, two studies were judged to perform inappropriate multiple analyses.[28,29] Judgement of bias in measurement of the outcome (domain 4, Table 2) showed most scattered results across studies (Online supplemental figure 1). A high risk of bias was found in three studies of which one had no information on measurements[34] and two showed inappropriate measurement methods of the outcome of interest.[28,33] In the study by Zult et al., only one trial per target was performed to estimate JPS,[33] while Baltaci et al.  used a test with presumably a high demand on motor and memory components,[28] without reporting its reliability or validity. The domain with least risk of bias was missing outcome data where all studies, except one,[32] had low risk of bias.

For peer review only - http://bmjopen.bmj.com/site/about/guidelines.xhtml

**Rehabilitation Programs**

The studies included a spectrum of rehabilitation programs employed to influence knee proprioception (Table 1). Only one study by Baltaci et al. investigated the effects of using feedback with an external focus in a simulated sport-specific gaming environment with Nintendo Wii Fit compared to conventional rehabilitation.[28] On the contrary, the remaining eight studies focused on having an internal focus (mainly related to the position of specific body parts) for neuromuscular training. Two studies[29,30] explored the effects of whole-body vibration therapy (WBVT) combined with or without conventional rehabilitation compared to conventional rehabilitation alone. Cho et al. compared closed kinetic chain exercises on a balance pad versus on a stable floor.[34] Risberg et al. compared the effects of a NT compared to strength training. In their neuromuscular program, the first half of the rehabilitation focused on exercises on a wobble board or trampoline and exercises to increase the range of motion, while the end of the program focused on specific training of plyometric, agility and sport-specific skills.[48] Beynnon et al. evaluated the effects of accelerated (19 weeks) vs. non-accelerated (32 weeks) programs of conventional training.[49] The timeframe and exercises in their experimental program ranged from 1-7 weeks for range of motion and muscle activation, 8-11 weeks for dynamic functional activities such as biking and jogging, and finally, 12-19 weeks for plyometric and agility drill exercises.[49] Kaya et al. studied the effects of neuromuscular (motor control) exercises for the lower limbs combined with standard rehabilitation compared with standard rehabilitation alone.[32] Shen et al. examined the outcome of standard rehabilitation combined with backward walking at 1.3 km/h on a treadmill for four groups (at four inclination angles 0°, 5°, 10°, and 15°, respectively) compared to standard rehabilitation in a comparator group.[31] Nevertheless, Zult et al. examined the effects of cross-education of strength training of the non-injured leg along with standard rehabilitation compared to standard rehabilitation alone.[33]

For peer review only

**Knee-specific Proprioceptive Measures**

Seven studies used active or passive JPS and all but one used (absolute) angular error as a variable to evaluate the outcome.[28-34] Conversely, one study used a computer program (monitored-rehab-system-software) to define a virtual line/route to allow joint repositioning within 30-70% knee range of motion with and without visual feedback.[28] The differences between visual and blinded trials (2 each) based on the deviations from the computer-generated line (in mm) were used to give information about the sense of proprioception.[28] All these studies used sitting or supine test position for assessing JPS. There were two to four predetermined target knee flexion angles across studies ranging from 15°-80°.[29-34] Moreover, two studies[28,29] used active knee motion and four used passive knee motion[30-33] to set the target angle. Whether Cho et al. used active or passive knee motion to set/reproduce the target angle seems ambiguous.[34] Four studies[28,29,32-34] used active knee motion and two[30,31] used passive knee motion to reproduce the target angle. The JPS method used by Zult et al. was presumed based on their reference to Hortobagyi et al.[50]

The angular error was measured with 1-6 trials per each angle and one study[33] randomized the order of the joint angles used. Eyes were blinded during the test in six studies[29-34] while one study used visual feedback when the individual was placing the knee joint in the target angle but no such feedback was given during reproduction of the target angle.[28] The difference between visual and non-visual trials was calculated in mm by the device as a measure of JPS.[28] A Biodex dynamometer (Biodex Medical Systems, Shirly, NY, USA) was used in five studies[29,30,32-34] to test JPS. Even so, one study used a continuous passive motion equipment[31] while another[28] employed a functional squat system (Monitored Rehab System, Haarlem, and the Netherlands) with a leg press machine and an associated computer program for assessing JPS.

For peer review only

291 Three studies[31,48,49] evaluated knee kinesthesia with the threshold to detect passive motion
292 (TTDPM) using a bespoke device,[48,49] or a continuous passive motion equipment.[31] The knee
293 joint was moved in flexion or extension at a constant angular velocity of 0.5°/s [48] or
294 0.1°/s.[31,49] While the participants were blindfolded in two studies,[31,49] the other study did not
295 mention about visual feedback.[48] In all three studies, the tests were performed three times in
296 each direction (flexion and/or extension) for both legs but whether the order of direction or
297 leg was randomized is not reported. In the study by Risberg et al.,[48] TTDPM data were
298 missing for 27 out of 74 participants because of device failure, which might lower the power
299 of the study.

300 ***Effects of NT on Knee Proprioception in Individuals with ACLR***

301 There were conflicting findings among the included studies for the effects of NT on
302 improving JPS, TTDPM and QFC. Overall, mean differences between groups indicated
303 inconsistent findings with an increase or decrease of JPS angular errors (one or more target
304 angles) by ≤2º, TTDPM by ≤1.5º, and QFC (concentric/eccentric/isometric contractions) by
305 ≤6 Nm following neuromuscular training.

306 Of the nine included articles, four reported reduction in JPS angular errors of ACLR knee at
307 one or more target angles (JPS at 45° but not 15° [34]; JPS at 60° but not 30° [29]; JPS at 15°, 45°,
308 75° [32]; JPS 20°, 50°, 80° [31]) and/or contralateral non-injured knee (JPS at 30° and 60° [29])
309 favoring the NT group (exercises on a balance pad [34], whole-body vibration therapy [29],
310 neuromotor control exercises [32] or backward treadmill walking [31]). Shen et al. also reported
311 improved TTDPM following backward treadmill walking.[31] When we calculated mean
312 differences for author-reported post-operative [29,32] or change (pre- vs. post-intervention)
313 scores [34] between groups for the ACLR leg with the Review Manager 5.3 software (the
314 Cochrane Collaboration), their 95% confidence intervals revealed no effects (see Table 1).

For peer review only

Moreover, the remaining five studies did not report significant differences in proprioception between groups.[28,30,33,48,49]

**Assessing Certainty in Evidence**

There were serious concerns with three GRADE domains (risk of bias, indirectness, and imprecision associated with the findings) across the seven studies that measured JPS (Tables 3 and 4). The certainty of evidence found was very low for the effects of NT on improving JPS following ACLR.

There were further serious concerns with all GRADE domains (risk of bias, inconsistency, indirectness, and imprecision associated with the findings) across the three studies measuring TTDPM (Tables 3 and 4). Therefore, the certainty of evidence found was very low for improving TTDPM in individuals with ACLR following NT (Table 3).
An overall judgement of some concerns based on the Cochrane ROB 2 tool (Table 2) was found for the study reporting changes in QFC following NT.[33] Available population, the magnitude and direction of effect, and effect estimates of QFC (Tables 1 and 3) are derived from only one study which reflect serious concerns. However, the participants with ACLR, intervention (cross-education of the quadriceps with standard rehabilitation), and QFC[33] are directly related to our research question. The certainty of evidence found was very low for improving QFC in individuals with ACLR following NT because only one relevant study was found.

**Discussion**

This review is the first, as far as we are aware, to address effects of neuromuscular rehabilitation training on knee proprioception in individuals with ACL injury. A previous review, however, summarized the effects of proprioceptive and balance exercises following

For peer review only

ACL injury/reconstruction on certain outcome measures (muscle strength, hop test, etc.) but other than knee-specific proprioception tests.[51] Another similar review did not find any RCTs in this area.[52] We identified nine studies employing a range of NT methods, of which all but one[48] were published within the past decade. Nevertheless, there were serious concerns with two or more GRADE domains (risk of bias, inconsistency, indirectness, or imprecision associated with the findings) across studies implying a very low certainty of evidence for improving JPS, TTDPM, and QFC of ACLR knee following NT.

***Effects of NT on Knee Proprioception in Individuals with ACLR***

Most of the employed NT programs did not influence proprioception compared to comparator interventions. Potential reasons for insignificant between-group differences include: 1) experimental and comparator programs (with exercises that are wholly or partly similar) which potentially might stimulate similar effects on proprioception in both programs;[28,30,32,34,48,49] 2) the exercises did not adequately stimulate proprioception sense;[33] 3) a lack of proprioception deficit following ACL injury (TTDPM similar between ACL-injured and contralateral uninjured knee[49]); 4) a lack of valid, sensitive and responsive knee-specific proprioception test methods; 5) a short follow-up period (a follow-up at least 18 months post-ACLR might be needed to regain proprioceptive function[53]) in most studies except two studies;[32,49] 6) type II errors arising from low sample sizes in most studies (with missing power or sample size calculations); and 7) adherence rates of participants to the prescribed program (only three studies have explicitly reported adherence rates to training sessions/exercises [Table 1]).[30,48,49] The heterogeneity of interventions, methodological limitations, inconsistency in the magnitude and direction of effects, and imprecision of effect estimates, found in this review, preclude recommendation of one optimal NT intervention for improving proprioception following ACL injury in clinical practice.

For peer review only

### *Risk of Bias in the Included Studies*

Bias in selection of the reported variables/results due to absence of a pre-specified plan of analyses applied to all but one study,[33] and none had published a trial protocol in a scientific journal although two studies were registered in a trial registry.[31,33] A possible reason for the absence of registration for most studies in this review may be that all but three studies were older than five years. Yet, one latest published study did not report trial registration.[32]

Another concern was the method used to measure JPS. For instance, estimates of JPS based on 3-5 repetitions, in clinical trials, may be insufficient.[54] According to Selfe et al. five repetitions in active knee JPS test, and six when performed passively, are necessary to ensure a consistent proprioception score.[55] However, this was only met in two included studies.[29,32]

All studies used absolute angular error (AAE) for measuring JPS acuity which represents a task-oriented approach to studying performance skill, in contrast to a process-orientation in which underlying processes are in focus. The inconsistency in performance, i.e., response variability (variable error), may reflect noise in sensory signal and its processing[56] and thus be a more process-oriented outcome than AAE. To understand possible underlying mechanisms, it would be advantageous to combine task- and process-oriented measures. In general, method descriptions of proprioception tests were short and, in some studies, deficient, lacking information about factors that could influence the results. One such factor was randomization of the order of target positions (cf. Zult et al.),[33] which is required to minimize the effect of memory and reduce motor elements of the test. This is particularly applicable in tests with active positioning, which was the case for most studies, enabling central motor programs.[57] Inadequate reporting of the proprioception tests would hinder their replication and raise risk of bias rating. Moreover, Kaya et al.  reported only post-intervention JPS scores, precluding baseline scores, despite claiming their study to be an RCT.[32]

For peer review only

Among seven RCTs[28-34] investigating changes in JPS following NT, five RCTs were found to have a high risk of bias while the remaining two studies have some concerns based on the Cochrane ROB 2 tool (Table 2). Therefore, included RCTs have been judged to have very serious methodological limitations in the GRADE evidence synthesis.

***Mechanisms Underpinning NT Following ACLR***

Two of the included studies evaluated the effects of WBVT;[29,30] however, only one found a favorable effect on proprioception (JPS – target angle 60°).[29] Two factors may contribute to the different findings between these studies. First, time point at which WBVT was given: Fu et al. employed WBVT at one-month post-ACLR for 2 months and evaluated JPS at 3 and 6 months after the surgery (Table 2). [30] On the other hand, Moezy et al. gave WBVT at 3 months post-ACLR for one-month and assessed JPS at 4 months after the surgery.[29] It seems starting WBVT at 3 months, rather than at one-month, post-ACLR might have better on improving knee JPS. Second, the use of active[29] or passive[30] knee movement when testing JPS. Active tests stimulate both joint and muscle-tendon mechanoreceptors and induce alpha-gamma co-activation while passive tests assess joint receptors to a higher degree[10,58] which potentially could mean a higher sensitivity of the active test.

WBVT has shown effects on body posture, flexibility, proprioception (TTDPM in patients with osteoarthritis), coordination and muscle power.[59-61] It has been promoted as an effective method to induce a reflex muscle contraction in subjects with difficulties to evoke voluntary contractions.[62] The mechanism behind the improvements can be that the mechanical stimuli stimulate primary endings of muscle spindles, especially type II fibers, which activate a-motor neurons. This could potentially stimulate central motor command, which facilitates increased muscle activation and voluntary movements.[59]

For peer review only

Cho et al. showed a significant effect on knee proprioception (JPS and TTDPM) with closed kinetic chain exercises on a balance pad/board.[34] Exercises on a balance board are widely used to improve proprioception.[38,51] In this review, a few NT programs included, amongst other exercises, balance training with or without a balance pad/board.[28,32,34,48,49] Additionally, one study claimed backward walking, a closed kinematic chain exercise, to stimulate joint/muscle receptors and sensory afferents to the CNS and augment proprioceptive and balance training.[31] Among these studies, all but one,[31] did not show significant mean differences between groups in proprioception calculated using the Review Manager 5.3 software (the Cochrane Collaboration) (see Table 1 and supplementary files). Different designs and levels of difficulty of the execution were found (e.g. a simple static balance task [with and without visual input], dynamic exercises performed on the balance board, backward walking on a treadmill, etc.).

There is a challenge to transfer the rehabilitation in the clinic to automatic movements required for athletic activities.[18,63] Wii Fit or similar games have the potential to combine feedback with an external focus in a sport-specific environment,[28] supporting the use of such training tools. However, a study on Nintendo Wii Fit training did not support its use for improving knee proprioception following ACLR.[28] Newer technology with stroboscopic eyewear might have the potential to decrease visual input without fully occluding it, making it possible to use them in sport specific rehabilitation. To prepare the individual for complex athletic environments and reduce re-injury risk, rehabilitation might focus on NT with reduced demands on visual inputs and enhance automatic movement control with cognitive demands included.[18] Whether such NT training improves knee proprioception and, how this should be assessed in the best way,[13] are yet to be determined.

***The Ability of Tests to Discern Changes in Proprioception Following NT***

For peer review only

There is neither a gold standard proprioception test (targeting JPS, kinesthesia, force sense) nor a standard procedure with established psychometric properties to test each proprioception sense following ACL injury. In this review, JPS and TTDPM were commonly reported. The Ruffini and Golgi receptors are slow-adapting receptors, responding to a change in joint position. Nevertheless, the Pacinian receptors that respond to low degrees of joint stress are more sensitive to rapid changes in accelerations and contribute to a low TTDPM.[2,64] JPS has been reported to detect a greater difference in knee proprioception than TTDPM following an ACL injury.[2] However, our findings remain equivocal regarding the outcomes of JPS or TTDPM following NT.

Knee-specific proprioception tests provide an indirect measure of proprioception involving the process of the CNS.[10] Psychosocial factors,[65] pain and preinjury motor skills may influence the central mechanisms and the outcome of such tests following NT. Knee-specific proprioception tests are designed to exclude motor skills, but how successful that exclusion works, remains unclear.

***Limitations and Future Recommendations***

The nine included studies looked at only individuals with ACLR but not those managed conservatively following ACL injury. Owing to clinical heterogeneity of interventions and outcome measurements, meta-analyses were precluded from the GRADE evidence synthesis. The included studies had methodological limitations (high risk of bias or some concerns) and all, but two studies,[31,33] had not pre-registered/published their protocol. There is a need for high quality RCTs with low risk of bias in this area.

Grey literature was not included in the current review which could be seen as a limitation. The most common reason for exclusion of clinical trials in this review was that they did not evaluate the effects of NT following ACLR with a knee-specific proprioception test. Perhaps,

For peer review only

the lack of consensus regarding the most appropriate, valid, reliable and responsive proprioception tests, number of target angles or most responsive target angles (low vs. high) might have precluded such outcomes in these studies. Therefore, psychometric properties of such tests must be established.[13]

When designing rehabilitation programs with long-term follow-up, aberrations in neuromotor control as well as neuroplastic changes should preferably be addressed. To reflect a wide spectrum of individual impairments, further research should investigate differences in individuals with ACL injuries managed with surgical (graft types) or conservative treatment, both sexes, athletes and non-athletes of different ages. Future studies might assess neuromotor control in functional tasks rather than relying on knee-specific proprioception tests, given the challenges of isolating the proprioceptive ability.

For peer review only

## Conclusion

The existing nine studies on individuals with an ACL reconstruction using heterogeneous interventions and knee-specific proprioception measures revealed a very low certainty in current evidence for employing NT programs to improve knee proprioception. The GRADE evidence synthesis revealed a high risk of bias or some concerns, indirect evidence, conflicting findings, and imprecision of effect estimates in the included studies. Well-designed RCTs with homogenous populations (having ACL injury managed with or without reconstruction), novel/well-designed NT interventions, and valid proprioception measures are warranted to substantiate conclusive evidence in this area.

## Contributors

AA and CKH conceived the idea of the project. AA, MB, SM and CKH were responsible for designing the review and conceptualising the initial review protocol. AA led the writing

of the manuscript. MB, SM and CKH contributed to writing the manuscript. AA, MB and CKH have reviewed and revised the manuscript for intellectual content. All authors approved the final version of the manuscript. AA is the guarantor of this work.

**Funding**

The work was supported by the Swedish Research Council (2017-00892); Region Västerbotten (ALF 7003575; Strategic funding VLL-358901; Project. No. 7002795); the Swedish Research Council for Sports Science (CIF P2019 0068); and King Gustaf V and Queen Victoria's Foundation of Freemasons 2019 (Häger). The funders were not involved in the conception, design, execution and writing of the review.

**Data availability statement**

All data relevant to the study are included in the article or uploaded as supplementary information. A detailed protocol of the review has been registered in PROSPERO (CRD42018107349).

**Competing interests**

None declared.

**Patient consent for publication**

Not required.

For peer review only

**Figure caption**

Figure 1. Flow diagram depicting the steps involved in screening and selection of eligible articles

For peer review only

## References


1. Kapreli E, Athanasopoulos S, Gliatis J, et al. Anterior Cruciate Ligament Deficiency Causes Brain Plasticity: A Functional MRI Study. *Am J Sports Med.* 2009;37(12):2419-2426.

2. Relph N, Herrington L, Tyson S. The effects of ACL injury on knee proprioception: a meta-analysis. *Physiotherapy.* 2014;100(3):187-195.

3. Sanders TL, Maradit Kremers H, Bryan AJ, et al. Incidence of anterior cruciate ligament tears and reconstruction: a 21-year population-based study. *Am J Sports Med.* 2016;44(6):1502-1507.

4. Kobayashi H, Kanamura T, Koshida S, et al. Mechanisms of the anterior cruciate ligament injury in sports activities: a twenty-year clinical research of 1,700 athletes. *J Sports Sci Med.* 2010;9(4):669-675.

5. Johnston JT, Mandelbaum BR, Schub D, et al. Video analysis of anterior cruciate ligament tears in professional American football athletes. *Am J Sports Med.* 2018;46(4):862-868.

6. Acevedo RJ, Rivera-Vega A, Miranda G, Micheo W. Anterior cruciate ligament injury: identification of risk factors and prevention strategies. *Curr Sports Med Rep.* 2014;13(3):186-191.

7. Ardern CL, Taylor NF, Feller JA, Whitehead TS, Webster KE. Sports participation 2 years after anterior cruciate ligament reconstruction in athletes who had not returned to sport at 1 year: a prospective follow-up of physical function and psychological factors in 122 athletes. *Am J Sports Med.* 2015;43(4):848-856.

8. Gao F, Zhou J, He C, et al. A Morphologic and Quantitative Study of Mechanoreceptors in the Remnant Stump of the Human Anterior Cruciate Ligament. *Arthroscopy.* 2016;32(2):273-280.

9. Dhillon MS, Bali K, Prabhakar S. Differences among mechanoreceptors in healthy and injured anterior cruciate ligaments and their clinical importance. *Muscles Ligaments Tendons J.* 2012;2(1):38-43.

10. Röijezon U, Clark NC, Treleaven J. Proprioception in musculoskeletal rehabilitation. Part 1: Basic science and principles of assessment and clinical interventions. *Man Ther.* 2015;20(3):368-377.

11. Kapreli E, Athanasopoulos S. The anterior cruciate ligament deficiency as a model of brain plasticity. *Med Hypotheses.* 2006;67(3):645-650.

12. Nakamae A, Adachi N, Ishikawa M, Nakasa T, Ochi M. No evidence of impaired proprioceptive function in subjects with anterior cruciate ligament reconstruction: a systematic review. *J ISAKOS.* 2017;2(4):191.


For peer review only

13. Arumugam A, Strong A, Tengman E, Röijezon U, Häger CK. Psychometric properties of knee proprioception tests targeting healthy individuals and those with anterior cruciate ligament injury managed with or without reconstruction: a systematic review protocol. *BMJ Open.* 2019;9(4):e027241.

14. Stensdotter AK, Tengman E, Olofsson LB, Häger C. Deficits in single-limb stance more than 20 years after ACL injury. *Europ J Physiother.* 2013;15(2):78-85.

15. Fukunaga T, Johnson CD, Nicholas SJ, McHugh MP. Muscle hypotrophy, not inhibition, is responsible for quadriceps weakness during rehabilitation after anterior cruciate ligament reconstruction. *Knee Surg Sports Traumatol Arthrosc.* 2019;27(2):573-579.

16. Zebis MK, Andersen LL, Bencke J, Kjaer M, Aagaard P. Identification of athletes at future risk of anterior cruciate ligament ruptures by neuromuscular screening. *Am J Sports Med.* 2009;37(10):1967-1973.

17. Grooms DR, Page SJ, Nichols-Larsen DS, Chaudhari AM, White SE, Onate JA. Neuroplasticity Associated With Anterior Cruciate Ligament Reconstruction. *J Orthop Sports Phys Ther*. 2017;47(3):180-189.

18. Grooms D, Appelbaum G, Onate J. Neuroplasticity Following Anterior Cruciate Ligament Injury: A Framework for Visual-Motor Training Approaches in Rehabilitation. *J Orthop Sports Phys Ther*. 2015;45(5):381-393.

19. Clark NC, Röijezon U, Treleaven J. Proprioception in musculoskeletal rehabilitation. Part 2: clinical assessment and intervention. *Man Ther*. 2015;20(3):378-387.

20. Risberg MA, Mork M, Jenssen HK, Holm I. Design and implementation of a neuromuscular training program following anterior cruciate ligament reconstruction. *J Orthop Sports Phys Ther*. 2001;31(11):620-631.

21. Moutzouri M, Gleeson N, Billis E, Panoutsopoulou I, Gliatis J. What is the effect of sensori-motor training on functional outcome and balance performance of patients' undergoing TKR? A systematic review. *Physiotherapy.* 2016;102(2):136-144.

22. Ordahan B, Kucuksen S, Tuncay I, Salli A, Ugurlu H. The effect of proprioception exercises on functional status in patients with anterior cruciate ligament reconstruction. *J Back Musculoskelet Rehabil*. 2015;28(3):531-537.

23. Moher, D., Liberati, A., Tetzlaff, J., Altman, D. G., & Prisma Group. (2009). Preferred reporting items for systematic reviews and meta-analyses: the PRISMA statement. *PLoS med*. 2009;6:e1000097.

24. Campbell M, McKenzie JE, Sowden A, et al. Synthesis without meta-analysis (SWiM) in systematic reviews: reporting guideline. *BMJ.* 2020;368.

25. Methley AM, Campbell S, Chew-Graham C, McNally R, Cheraghi-Sohi SJBhsr. PICO, PICOS and SPIDER: a comparison study of specificity and sensitivity in three search tools for qualitative systematic reviews. *BMC Health Serv Res.* 2014;14(1):579.

For peer review only

26. Sterne JAC, Savović J, Page MJ, et al. RoB 2: a revised tool for assessing risk of bias in randomised trials. *BMJ.* 2019;366.

27. Guyatt GH, Oxman AD, Vist GE, et al. GRADE: an emerging consensus on rating quality of evidence and strength of recommendations. *BMJ.* 2008;336(7650):924-926.

28. Baltaci G, Harput G, Haksever B, Ulusoy B, Ozer H. Comparison between Nintendo Wii Fit and conventional rehabilitation on functional performance outcomes after hamstring anterior cruciate ligament reconstruction: prospective, randomized, controlled, double-blind clinical trial. *Knee Surg Sports Traumatol Arthrosc*. 2013;21(4):880-887.

29. Moezy A, Olyaei G, Hadian M, Razi M, Faghihzadeh S. A comparative study of whole body vibration training and conventional training on knee proprioception and postural stability after anterior cruciate ligament reconstruction. *Br J Sports Med.* 2008;42(5):373-385.

30. Fu CLA, Yung SHP, Law KYB, et al. The Effect of Early Whole-Body Vibration Therapy on Neuromuscular Control After Anterior Cruciate Ligament Reconstruction: A Randomized Controlled Trial. *Am J Sports Med*. 2013;41(4):804-814.

31. Shen M, Che S, Ye D, Li Y, Lin F, Zhang Y. Effects of backward walking on knee proprioception after ACL reconstruction. *Physiother Theory Pract.* 2019.

32. Kaya D, Guney-Deniz H, Sayaca C, Calik M, Doral MN. Effects on Lower Extremity Neuromuscular Control Exercises on Knee Proprioception, Muscle Strength, and Functional Level in Patients with ACL Reconstruction. *Biomed Res Int.* 2019:1-7.

33. Zult T, Gokeler A, van Raay J, et al. Cross-education does not accelerate the rehabilitation of neuromuscular functions after ACL reconstruction: a randomized controlled clinical trial. *Eur J Appl Physiol*. 2018;118(8):1609-1623.

34. Cho SH, Bae CH, Gak HB. Effects of closed kinetic chain exercises on proprioception and functional scores of the knee after anterior cruciate ligament reconstruction. *J Phys Ther Sci.* 2013;25(10):1239-1241.

35. Laboute E, Verhaeghe E, Ucay O, Minden A. Evaluation kinaesthetic proprioceptive deficit after knee anterior cruciate ligament (ACL) reconstruction in athletes. *J Exp Orthop.* 2019;6(1).

36. Feil S, Newell J, Minogue C, Paessler HH. The effectiveness of supplementing a standard rehabilitation program with, superimposed neuromuscular electrical stimulation after anterior cruciate ligament reconstruction. *Am J Sports Med.* 2011;39(6):1238-1247.

37. Ihara H, Nakayama A. Dynamic joint control training for knee ligament injuries. / L' entrainement au controle dynamique de l' articulation dans le cas des traumatismes ligamentaires du genou. *Am J Sports Med*.1986;14(4):309-315.

For peer review only

38. Vathrakokilis K, Malliou P, Gioftsidou A, Beneka A, Godolias G. Effects of a balance training protocol on knee joint proprioception after anterior cruciate ligament reconstruction. *J Back Musculoskelet Rehabil.* 2008;21(4):233-237.

39. Saha S, Adhya B, Dhillon MS, Saini A. A Study on the Role of Proprioceptive Training in Non Operative ACL Injury Rehabilitation. *Indian J Physiother Occup Ther.* 2015;9(3):226-231.

40. Lim J-M, Cho J-J, Kim T-Y, Yoon B-C. Isokinetic knee strength and proprioception before and after anterior cruciate ligament reconstruction: A comparison between home-based and supervised rehabilitation. *J Back Musculoskelet Rehabil.* 2019;32(3):421-429.

41. Liu-Ambrose T, Taunton JE, MacIntyre D, McConkey P, Khan KM. The effects of proprioceptive or strength training on the neuromuscular function of the ACL reconstructed knee: a randomized clinical trial. *Scand J Med Sci Sports.* 2003;13(2):115-123.

42. Lee SJ, Ren Y, Chang AH-I, Geiger F, Zhang L-Q. Effects of pivoting neuromuscular training on pivoting control and proprioception. *Med Sci Sports Exerc.* 2014;46(7):1400-1409.

43. Peultier-Celli L, Mainard D, Wein F, et al. Comparison of an innovative rehabilitation, combining reduced conventional rehabilitation with balneotherapy, and a conventional rehabilitation after anterior cruciate ligament reconstruction in athletes. *Front Surg.* 2017;4:61.

44. Faggal MS, Abdelsalam MS, Adel Elhakk SM, Mahmoud NF. Proprioceptive training after ACL reconstruction: Standard versus stump preservation technique. *Physiother Pract Res.* 2019;40(1):69-75.

45. San Martín-Mohr C, Cristi-Sánchez I, Pincheira PA, Reyes A, Berral FJ, Oyarzo C. Knee sensorimotor control following anterior cruciate ligament reconstruction: A comparison between reconstruction techniques. *PLoS One.* 2018;13(11):e0205658.

46. Büyükturan Ö, Büyükturan B, Kurt EE, Yetis M. Effects of Tai Chi on partial anterior cruciate ligament injury: A single-blind, randomized-controlled trial. *Turk J Phys Med Rehabil.* 2019;65(2):160-168.

47. Wang L. Immediate effects of neuromuscular joint facilitation intervention after anterior cruciate ligament reconstruction. *J Phys Ther Sci.* 2016;28(7):2084-2087.

48. Risberg MA, Holm I, Myklebust G, Engebretsen L. Neuromuscular training versus strength training during first 6 months after anterior cruciate ligament reconstruction: a randomized clinical trial. *Phys Ther.* 2007;87(6):737-750.

49. Beynnon BD, Johnson RJ, Naud S, et al. Accelerated versus nonaccelerated rehabilitation after anterior cruciate, ligament reconstruction:A prospective, randomized, double-blind investigation evaluating knee joint laxity using roentgen, stereophotogrammetric analysis. *Am J Sports Med.* 2011;39(12):2536-2548.

For peer review only

50. Hortobágyi T, Garry J, Holbert D, Devita P. Aberrations in the control of quadriceps muscle force in patients with knee osteoarthritis. *Arthritis Care Res*. 2004;51(4):562-569.

51. Cooper RL, Taylor NF, Feller JA. A systematic review of the effect of proprioceptive and balance exercises on people with an injured or reconstructed anterior cruciate ligament. *Res Sports Med.* 2005;13(2):163-178.

52. Baltaci G, Kohl HW. Does proprioceptive training during knee and ankle rehabilitation improve outcome? *Phys Ther Rev.* 2003;8(1):5-16.

53. Iwasa J, Ochi M, Adachi N, Tobita M, Katsube K, Uchio Y. Proprioceptive improvement in knees with anterior cruciate ligament reconstruction. *Clin Orthop Relat Res*. 2000(381):168-176.

54. Han J, Waddington G, Adams R, Anson J, Liu Y. Assessing proprioception: A critical review of methods. *J Sport Health Sci*. 2016;5(1):80-90.

55. Selfe J, Callaghan M, McHenry A, Richards J, Oldham J. An investigation into the effect of number of trials during proprioceptive testing in patients with patellofemoral pain syndrome. . *J Orthop Res.* 2006;24(6):1218-1224.

56. van Beers RJ, Sittig AC, van der Gon Denier JJ. How humans combine simultaneous proprioceptive and visual position information. *Exp Brain Res.* 1996;111(2):253-261.

57. Gandevia SC, Burke D. Does the nervous system depend on kinesthetic information to control natural limb movements? *Behav Brain Sci.* 1992;15:614-614.

58. Clark NC, Akins JS, Heebner NR, et al. Reliability and measurement precision of concentric-to-isometric and eccentric-to-isometric knee active joint position sense tests in uninjured physically active adults. *Phys Ther Sport.* 2016;18:38-45.

59. Avelar NC, Costa SJ, da Fonseca SF, et al. The effects of passive warm-up vs. whole-body vibration on high-intensity performance during sprint cycle exercise. *J Strength Cond Res*. 2012;26(11):2997-3003.

60. Chow DHK, Lee TY, Pope MH. Effects of whole body vibration on spinal proprioception in healthy individuals. *Work.* 2018.

61. Trans T, Aaboe J, Henriksen M, Christensen R, Bliddal H, Lund H. Effect of whole body vibration exercise on muscle strength and proprioception in females with knee osteoarthritis. *Knee.* 2009;16(4):256-261.

62. Herrero AJ, Menendez H, Gil L, et al. Effects of whole-body vibration on blood flow and neuromuscular activity in spinal cord injury. *Spinal cord.* 2011;49(4):554-559.

63. Benjaminse A, Gokeler A, Dowling AV, et al. Optimization of the anterior cruciate ligament injury prevention paradigm: novel feedback techniques to enhance motor learning and reduce injury risk. *J Orthop Sports Phys Ther*. 2015;45(3):170-182.

For peer review only

691 64. Ochi M, Iwasa J, Uchio Y, Adachi N, Sumen Y. The regeneration of sensory neurones in the reconstruction of the anterior cruciate ligament. *J Bone Joint Surg Br*. 1999;81(5):902-906.

694 65. Louw A, Zimney K, Puentedura EJ, Diener I. The efficacy of pain neuroscience education on musculoskeletal pain: A systematic review of the literature. *Physiother Theory Pract*. 2016;32(5):332-355.

For peer review only

698 **Table 1. Summary of study characteristics**

| Study citation | Sample size[a], age (mean ± SD), gender; ACLR (Graft) | Intervention; Adherence to prescribed exercises/ training | Comparator; Adherence to prescribed exercises/ training | Knee-specific proprioception test; outcome | Between-group (experimental vs. control) comparisons of ACL-injured (reconstructed) limb - mean difference (95% confidence interval)[b] |
|---|---|---|---|---|---|
| **Baltaci et al. (2013)** [28] | Exp: n=15, 28.6±6.8 years, 15 men; Com: n=15, 29.3±5.7 years, 15 men; ACLR (hamstring tendon graft). | Nintendo Wii Fit training: 3 times/week; 60 min/session; from week 1-12 after ACLR. **Adherence:** NR | Conventional rehabilitation: Week 1-12 after ACLR; **Adherence:** NR | **Proprioception test:** JPS (ipsilateral replication method); **Body position:** NR; **Instrument:** Monitored Rehab System with a leg press machine and a computer game; **Procedure:** Active-active, with and without blindfolding of the eyes (2 trials each); **Starting angle (SA):** NR; **Target angle (TA):** NR; **Outcome measure:** absolute angular error (AAE; difference between visual and non-visual results for each leg) | **JPS[c] at 12 weeks post-intervention:** 1.90 [-31.20, 35.00] 33.30 [-28.02, 94.62] |

For peer review only

| | | | | | |
|---|---|---|---|---|---|
| **Beynnon et al. (2011)** [49] | Int: n=19, 29.7±10.1 years, 13 males, 6 females; Com: n=17, 30.2±9.9 years, 9 males, 8 females; ACLR (patellar tendon graft) | Accelerated rehabilitation: daily exercises at home + 3 times/week exercises under supervision from week 1-19 after ACLR; **Adherence:** 94% (range, 25%-292%) over 19 weeks | Non-accelerated rehabilitation: daily exercises at home + 3 times/week exercises under supervision from Week 1-32 after ACLR; **Adherence:** 53% (range, 13%-108%) over 32 weeks | **Proprioception test:** TTDPM; **Body position:** Seated; **Instrument:** A customized joint motion detection system; **Procedure:** passive movement of the knee into flexion or extension (3 trials for both ACL-reconstructed and contralateral uninjured knees) with eyes blindfolded; **SA:** NR; **Angular velocity:** 0.1°/s; **Outcome measure:** Threshold angle (difference between the initial angle [SA] and the angle at which the test was stopped) to detect passive knee motion into flexion or extension (mean of the three trials in one direction). | **TTDPM (°)[c] at 24 months post-ACLR:** **SA (NR):** 0.09 [-0.42, 0.60] |
| **Cho et al. (2013)** [34] | Int: n=14, 29.92±5.46 years; 14 males; Com: n=14, 28.78±7.24 years; 14 males; ACLR (NR). | Unstable exercise group: exercises performed on a balance pad or balance board; 60 min/session; 3 times/week early after injury, for 6 weeks; **Adherence:** NR | Stable exercise group: exercises performed on a stable floor: 3 times/week Early after injury, for 6 weeks; **Adherence:** NR | **Proprioception test:** JPS; **Body position:** seated (?); **Instrument:** Biodex dynamometer; **Procedure:** NR-active, with eyes blindfolded; **SA:** 90°; **TA:** 15°, 45°; **Outcome measure:** AAE (mean of the three trials at each angle). | **JPS (°)[d] at 6 weeks post intervention:** TA 15°: 0.14 [-0.69, 0.97] TA 45°: -0.87 [-1.91, 0.17] |

For peer review only - http://bmjopen.bmj.com/site/about/guidelines.xhtml

| | | | | | |
|---|---|---|---|---|---|
| **Fu et al. (2013)** [30] | Int: n=24, 23.3±5.2 years; Com: n=24, 25.2±7.3 years; ACLR (hamstring graft). | Conventional rehabilitation program + Whole-body vibration therapy: 2 times/week from week 5-13 after ACLR; **Adherence:** 83.2% over 12 weeks | Conventional rehabilitation program: week 5-13 after ACLR; **Adherence:** 84.4% over 12 weeks | **Proprioception test:** JPS; **Body position:** seated; **Instrument:** Biodex dynamometer; **Procedure:** passive-passive, eyes blindfolded; **SA:** 90°; **TA:** 30°, 60°; **Outcome measure:** AAE (mean of the three trials at each angle) | **JPS (°)[c] at 6 months post-ACLR:** **TA 30°:** -0.82 [-2.69, 1.05] **TA 60°:** -0.70 [-2.31, 0.91] |
| **Kaya et al. (2019)** [32] | Int (Group 1): n=20; 29.35±9.71 years; 20 males; Com (Group 2): n=20; 31.60±8.45 years; 20 males; ACLR (tibialis anterior allograft). | Standard rehabilitation program (0-2 weeks) + neuromuscular control exercises (3-36 weeks); **Adherence:** NR | Standard rehabilitation program (0-36 weeks); **Adherence:** NR | **Proprioception test:** JPS; **Body position:** seated (?); **Instrument:** Biodex dynamometer; **Procedure:** passive-active, eyes blindfolded; **SA:** 90°; **TA:** 15°, 45°, 75°; **Outcome measure:** AAE (mean of six trials at each angle) | **JPS (°)[c] at 24 months post-ACLR:** **TA 15°:** -1.51 [-3.30, 0.28] **TA 45°:** -1.69 [-5.06, 1.68] **TA 75°:** -1.30 [-3.34, 0.74] |
| **Moezy et al. (2008)** [29] | Int: n=12, 24.51±3.38 years; Com: n=11, 22.70±3.77 years; ACLR (patellar tendon graft) | Whole-body vibration therapy: 3 times/week from week 12-16 after ACLR; **Adherence:** NR | Conventional strengthening exercises program: 3 sessions/week Week 12-16 after ACLR; **Adherence:** NR | **Proprioception test:** JPS; **Body position:** seated; **Instrument:** Biodex dynamometer; **Procedure:** active-active, eyes blindfolded; **SA:** 90°; **TA:** 30°, 60°; **Outcome measure:** AAE (mean of five trials at each angle for both ACL- | **JPS (°)[e,d] at 4 months post-ACLR:** **TA 30°:** 1.66 [-0.40, 3.72] **TA 60°:** 3.03 [1.54, 4.52] |

| | | | | | |
|---|---|---|---|---|---|
| | | | | reconstructed and contralateral uninjured knees) | |
| **Risberg et al. (2007)** [48] | Int: n = 39; 3 females - 27.2 (range: 20.6-37.9) years and 26 males - 27.7 (16.7-39.6) years; Com: n=35, 14 females - 26.5 (19.8-38.0) years and 21 males - 31.2 (19.4-40.3) years; ACLR (patellar tendon graft) | Neuromuscular training program: 2-3 times/week from week 1-24 after ACLR; **Adherence:** 71% over ~20 weeks | Traditional strength training: 2-3 times/week from week 1-24 after ACLR; **Adherence:** 91% over ~20 weeks | **Proprioception test:** TTDPM; **Body position:** NR; **Instrument:** a customized TTDPM device; **Procedure:** passive movement of the knee into flexion and extension (three trials for each direction for both ACL-injured knees and contralateral uninjured knees); no information on blindfolding of eyes; **SA:** 15°; **Angular velocity:** 0.5°/s; **Outcome measure:** Threshold angle (difference between the SA and the angle at which the test was stopped) to detect passive knee motion into flexion or extension mean of the three trials in one direction (mean of three trials for each angle in each direction). | **TTDPM (°)[c] at 6 months post-ACLR:** **SA 15°:** -0.02 [-0.39, 0.35] (Note: TTDPM data were available only for the first 47 participants out of 74 in total). |
| **Shen et al. (2019)** [31] | Int (A): n=10; 36.6±12.1 years; 5 male, 5 females. Int (B): n=11; 37.5±9.39 years; 6 male, 5 females. Int (C): n=11; 34±10.29 years; 7 male, 4 females. | Standard rehabilitation + backward walking on the treadmill: Int. groups A, B, C, and D underwent backward walking | Standard rehabilitation with range of motion exercises, power exercises, walking, and cycling (duration and other | **Proprioception test 1:** JPS; **Body position:** supine lying; **Instrument:** continuous passive motion device; **Procedure:** passive-passive, eyes blindfolded; **SA:** 0°; **TA:** 20°, 50°, 80°, **Outcome measure:** AAE (mean of the three trials at each angle for ACL-injured knees?). | **Int (A) vs. Com group at 1 month post-intervention[d]:** **JPS (°)[c]:** **TA 20°:** -1.40 [-2.59, -0.21] **TA 50°:** -1.36 [-2.35, -0.37] **TA 80°:** -1.28 [-2.31, -0.25] **TTDPM (°)[c]:** |

| | | | | | |
|---|---|---|---|---|---|
| | Int (D): (n=10); 32.9±11.45 years; 6 male, 4 females. Com: n=10; 35.5±10.1 years; 7 male, 3 females;<br><br>ACLR (patellar tendon graft, hamstring tendon graft, allograft) | training at 1.3 km/h at different inclination angles of the treadmill (0°, 5°, 10°, and 15°, respectively); 20 min/day, 5 days/week for 4 weeks; **Adherence:** NR | parameters: NR); **Adherence:** NR | **Proprioception test 2:** TTDPM; **Body position:** Supine lying; **Instrument:** continuous passive motion device; **Procedure:** passive movement of the knee into flexion (3 times for each angle for ACL-injured knees?) with eyes blindfolded; **SA:** 20°, 50°, 80°; **Angular velocity:** 1°/s; **Outcome measure:** Threshold angle to detect passive knee motion into flexion (mean of three trials for each angle in one direction). | **SA 20°:** -1.34 [-2.11, -0.57] **SA 50°:** -1.40 [-2.05, -0.75] **SA 80°:** -1.29 [-2.00, -0.58] |
| **Zult et al. (2018)** [33] | Int: n =29 (22), 28±9 years; Com: n = 26 (21), 28±10 years n=24 males n=20 females<br><br>ACLR (patellar tendon graft/ hamstring tendon graft (SSG)/ Artificial) | Standard rehabilitation + Strength training of the quadriceps of the non-injured leg; 2 quadriceps exercises, 8–12 reps. maximum, 3 sets; 2 times/week from week 1-12 after ACLR; **Adherence:** NR explicitly; | Standard rehabilitation: 2 times/week from week 1-12 after ACLR; **Adherence:** NR explicitly; however, two participants who performed <26 sessions was excluded from analysis after week 26 | **Proprioception test 1:** JPS[g] **Body position:** seated (?); **Instrument:** Biodex dynamometer (?); **Procedure:** passive-active, eyes blindfolded (?); **SA:** 90° (?); **TA:** 15°, 30°, 45°, and 60°; **Outcome measure:** AAE (one trial at each angle).<br><br>**Proprioception test 2:** Quadriceps force control (QFC); **Body position:** seated (?); **Instrument:** Biodex dynamometer (?); **Procedure:** A target force matching task with the target set at 20% MVC for | **JPS (°)[e] at 26 weeks post-ACLR:** **TA 15°:** 1.00 [-1.12, 3.12] **TA 30°:** 2.00 [-0.12, 4.12] **TA 45°:** -1.00 [-3.39, 1.39] **TA 60°:** -1.00 [-2.79, 0.79]<br><br>**QFC (Nm)[e,f] at 6 months (26 weeks) post-ACLR:** **Concentric 60°/s:** 6.00 [0.67, 11.33] **Eccentric 60°/s:** -1.00 [-3.99, 1.99] |

| | | | |
|---|---|---|---|
| | however, one participant who performed <26 sessions was excluded from analysis after week 26 | three isometric trials (at 65° of knee flexion [5 s duration]) and 40 Nm for dynamic trials (four concentric and eccentric trials at 20°/s from 10°-90° knee flexion) (20°/s between 10° and 90° of knee flexion); **Outcome measure:** force accuracy (absolute error) determined over the terminal 3 s data for isometric trials (at 65° knee flexion) and over the middle 2 s data for concentric and eccentric trials. | **Isometric:** 1.00 [-0.76, 2.76] |

[a]Included in analysis;
[b]Calculated with Review Manager (RevMan) 5.3 (The Cochrane Collaboration 2014, Nordic Cochrane Centre Copenhagen, Denmark);
[c]Mean difference between groups were calculated based on post-intervention/final follow-up scores reported by the authors;
[d]Difference between four intervention groups and the comparator group were same and so only one comparison is presented.
[e]Mean difference between groups were calculated based on change scores from baseline (pre- vs. post-intervention) reported by the authors;
[f]Quadriceps force accuracy; both legs (within each group) showed improved force control (22–34%) at 26 weeks post-surgery ($p < 0.050$) according to the authors;
[g]JPS method has been presumed based on authors' reference to the method employed by Hortobagyi et al.[50];
ACLR - anterior cruciate ligament reconstruction, Int – intervention group; com – comparator group; JPS - joint position sense, NR- not reported, TTDPM - threshold to detection of passive motion, min. - minutes, reps – repetitions.

For peer review only

**Table 2. Risk of bias assessment of included studies according to the Revised Cochrane risk-of-bias tool for randomized trials (RoB 2) - judgements in five domains and an overall judgement using the descriptors of low risk of bias (low), some concerns, and high risk of bias (High).**

| Included studies | Outcome variable | 1. Bias from the randomization process | 2. Bias due to deviations from intended interventions | 3. Bias due to missing outcome data | 4. Bias in measurement of the outcome | 5. Bias in selection of the reported result | Overall judgement |
|---|---|---|---|---|---|---|---|
| **Baltaci et al. 2013** [28] | JPS | High | Some concerns | Low | High | High | High |
| **Beynnon et al. 2011** [49] | TTDPM | Low | Low | Low | Low | Some concerns | Some concerns |
| **Cho et al. 2013**[34] | JPS | Some concerns | Some concerns | Low | High | Some concerns | High |
| **Fu et al. 2013**[30] | JPS | Low | Low | Low | Low | Some concerns | Some concerns |
| **Kaya et al. 2019**[32] | JPS | Some concerns | High | High | Low | Some concerns | High |
| **Moezy et al. 2008**[29] | JPS | Some concerns | Low | Low | Some concerns | High | High |
| **Risberg et al. 2007** [48] | TTDPM | Low | Low | Low | Low | Some concerns | Some concerns |
| **Shen et al. 2019**[31] | JPS | Some concerns | Low | Low | Low | Some concerns | Some concerns |
| | TTDPM | Some concerns | Low | Low | Low | Some concerns | Some concerns |
| **Zult et al. 2018**[33] | JPS | Low | Some concerns | Low | High | Some concerns | High |
| | QFC | Low | Some concerns | Low | Low | Some concerns | Some concerns |

JPS - joint position sense, TTDPM - threshold to detect passive motion, QFC - quadriceps force control.

For peer review only - http://bmjopen.bmj.com/site/about/guidelines.xhtml

**Table 3. Applying the GRADE approach to rate the certainty in evidence found in the review**

| Certainty assessment | | | | | | | № of patients | | Certainty |
|---|---|---|---|---|---|---|---|---|---|
| № of studies | Study design | Risk of bias | Inconsistency | Indirectness | Imprecision | Other considerations | Neuromuscular Training | Comparator Intervention | |
| **Knee joint position sense (JPS)** | | | | | | | | | |
| 7 | Randomized trials | very serious[a] | serious[b] | serious[c] | serious[d] | none | 139 | 105 | ⊕◯◯◯ VERY LOW |
| **Knee joint threshold to detect passive motion (TTDPM)** | | | | | | | | | |
| 3 | Randomized trials | serious[a] | serious[b] | serious[c] | serious[d] | none | 84 | 51 | ⊕◯◯◯ VERY LOW |
| **Quadriceps force control (QFC)** | | | | | | | | | |
| 1 | Randomized trial | serious[a] | serious[e] | not serious | serious[e] | none | 22 | 21 | ⊕◯◯◯ VERY LOW |

Note: GRADE domains are explained further in Table 4.

a. Included studies had a high risk of bias or some concerns based on the Cochrane ROB2 tool;
b. The direction and/or magnitude of effect was inconsistent across trials;
c. Clinical heterogeneity (of participants, interventions, and method of assessing outcome measures);
d. Number of participant <400 and/or wide 95% confidence intervals of effect size estimates;
e. Available population, the magnitude and direction of effect, and effect estimates come from only one study.

For peer review only

**Table 4. GRADE evaluation of the certainty in evidence for knee joint position sense (JPS)**

| GRADE domain | Reviewer judgment | Concerns about GRADE domains |
|---|---|---|
| | **Knee joint position sense (JPS)** | |
| **Risk of bias (methodological limitations)** | Among seven RCTs[28-34] reporting changes in JPS following neuromuscular training, five RCTs were found to have a high risk of bias while the remaining two studies have some concerns based on the Cochrane ROB 2 tool (see Table 2). Indeed, we judged that the included RCTs have very serious methodological limitations. | Very serious |
| **Inconsistency** | The direction and/or magnitude of effect on JPS was inconsistent across most of the included RCTs. In summary, the between-group comparisons of five RCTs showed borderline or no change in JPS angular errors of the ACLR knee for one or more target angles following interventions. We noted significant differences in reduction of JPS angular errors for all target angles favoring the intervention groups (backward treadmill walking or motor control exercises) in only two RCTs as reported by the authors.[31,32] In fact, Kaya et al. had reported only post-intervention scores but they neither reported nor compared the baseline scores (post-operative scores).[32] Two other studies [29,34] presented with insignificant effects at a low target angle (15° or 30°) and significant effects at a high target angle (45° or 60°) of JPS favoring the intervention group (whole-body vibration therapy[29] or exercises on a balance pad[34]). When we calculated mean differences for author-reported post-operative[32] or change (pre- vs. post-intervention) scores,[29,34] between groups for the ACLR leg with the Review Manager 5.3 software (the Cochrane Collaboration), their 95% confidence intervals revealed no effects. Overall, we judged the evidence to have serious inconsistency in the direction and/or magnitude of effects. | Serious |
| **Indirectness** | The participants (with ACLR [different grafts]), different neuromuscular training and comparator interventions, and knee specific JPS measures in the included studies provide evidence to the research question. However, the heterogeneity of interventions precludes recommendation of one optimal neuromuscular training intervention for clinical practice. In addition, variations in the methods of JPS measurements (active vs. passive angle reproduction, low vs. high target angles, etc.) precluded a meta-analysis. We judged the evidence to have serious indirectness especially owing to variations in | Serious |

For peer review only

| | | |
|---|---|---|
| | the interventions and outcome measures. | |
| **Imprecision** | A total of 244 patients was included from seven RCTs reporting changes in JPS following neuromuscular training (n = 139) or comparator interventions (n = 105). Most of the included trials reported non-significant results with wider 95% confidence intervals for one or more JPS (target) angles (see Table 1). Therefore, we judged the evidence to have serious imprecision. | Serious |
| **Other considerations** | Since negative and positive findings have been published, and a comprehensive search for RCTs has been done, we did not suspect a publication bias. | None |
| | **Knee joint threshold to detect passive motion (TTDPM)** | |
| **Risk of bias (methodological limitations)** | Three RCTs[31, 48, 49] reporting changes in TTDPM following neuromuscular training were found to show some concerns in risk of bias based on the Cochrane ROB 2 tool (see Table 2). We judged the included RCTs to be of serious methodological limitations. | Serious |
| **Inconsistency** | The direction and/or magnitude of effect was conflicting between the three RCTs. As two trials reported insignificant effects and one[41] reported significant effects (see Table 1), we judged the evidence to have serious inconsistency in the direction and/or magnitude of effects. | Serious |
| **Indirectness** | The participants (with ACLR [different grafts]), different neuromuscular training and comparator interventions, and knee specific TTDPM measures in the included studies provide some evidence to the research question in hand. However, the heterogeneity of interventions and TTDPM measurements (starting angles, angular velocity, etc.) precluded a meta-analysis. We judged the evidence to have serious indirectness especially owing to variations in the interventions and TTDPM methods. | Serious |
| **Imprecision** | A total of 135 patients was included in three RCTs reporting the effects of neuromuscular training (n = 84) or comparator interventions (n = 51) on TTDPM. Two trials[48,49] reported non-significant results while another one[31] reported significant effects which is evident with their confidence intervals (see Table 1). However, Shen et al. (2019) reporting significant effects on TTDPM included only 10 to 11 participants in each group while the other two studies with a relatively larger sample size declared | Serious |

For peer review only

| | | |
|---|---|---|
| | no significant effects on TTDPM. Therefore, we judged the evidence to have serious imprecision. | |
| **Other considerations** | As both negative and positive findings have been published, and a comprehensive search for RCTs has been done, we did not suspect a publication bias. | None |

ACLR - anterior cruciate ligament reconstruction, RCTs – randomized controlled trials, ROB – risk of bias

For peer review only

 

734 **Box 1. A list of acronyms used in the review**

| Acronym | Definition |
|---|---|
| ACL | Anterior cruciate ligament |
| ACLR | Anterior cruciate ligament reconstruction |
| AAE | Absolute angular error |
| CNS | Central nervous system |
| CT | Controlled Clinical trial |
| GRADE | Grading of recommendations, assessment, development and evaluation |
| JPS | Joint position sense |
| NT | Neuromuscular training |
| PRISMA | Preferred reporting items for systematic review and meta-analysis |
| PICOS | Participants, intervention, comparator, outcome measures, study design |
| QFC | Quadriceps force control |
| RCT | Randomized controlled trial |
| ROB | Risk of bias |
| TTDPM | Thresholds to detect passive motion |
| WBVT | Whole-body vibration therapy |

735

For peer review only

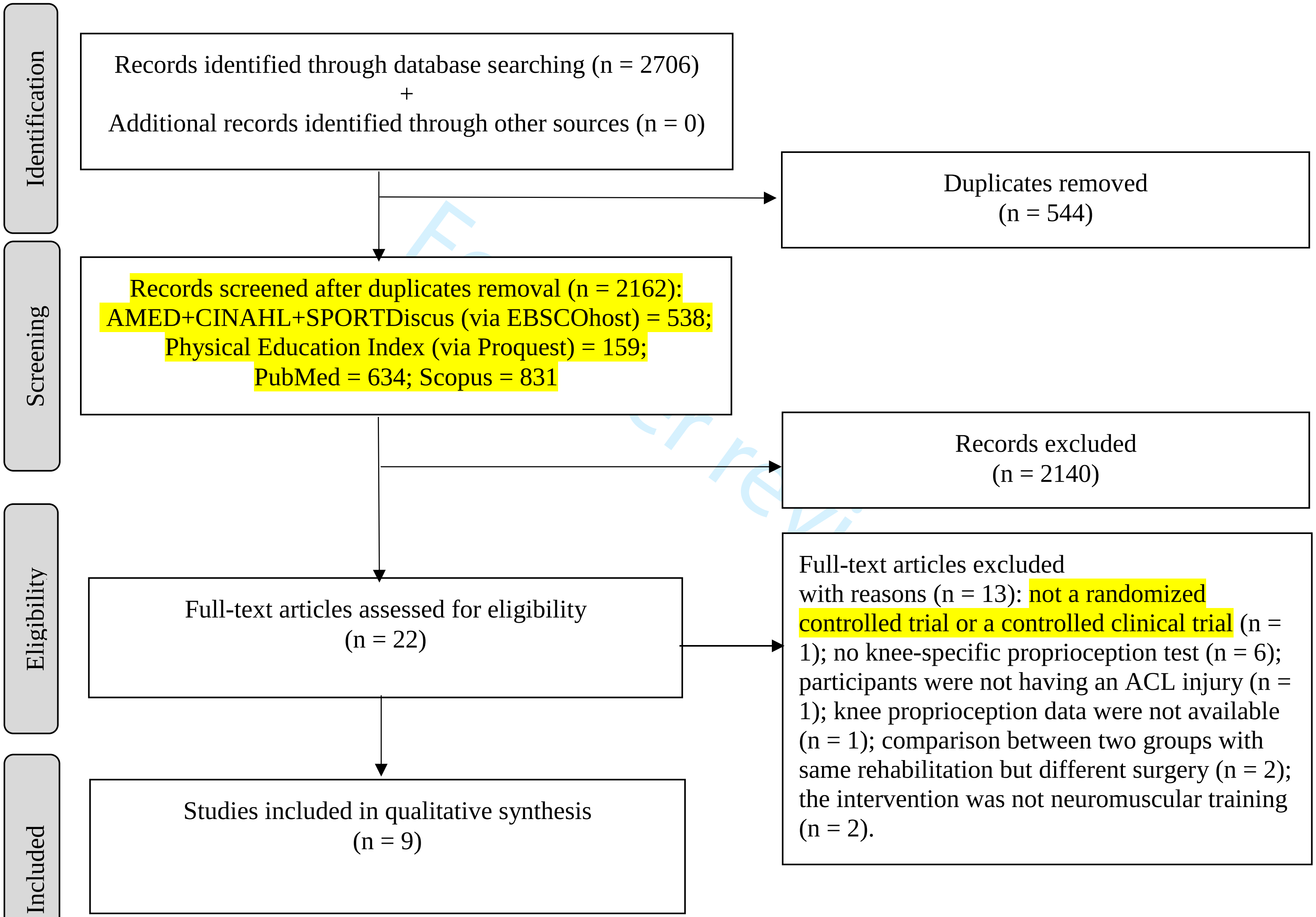


Figure 1. Flow diagram depicting the steps involved in screening and selection of eligible articles

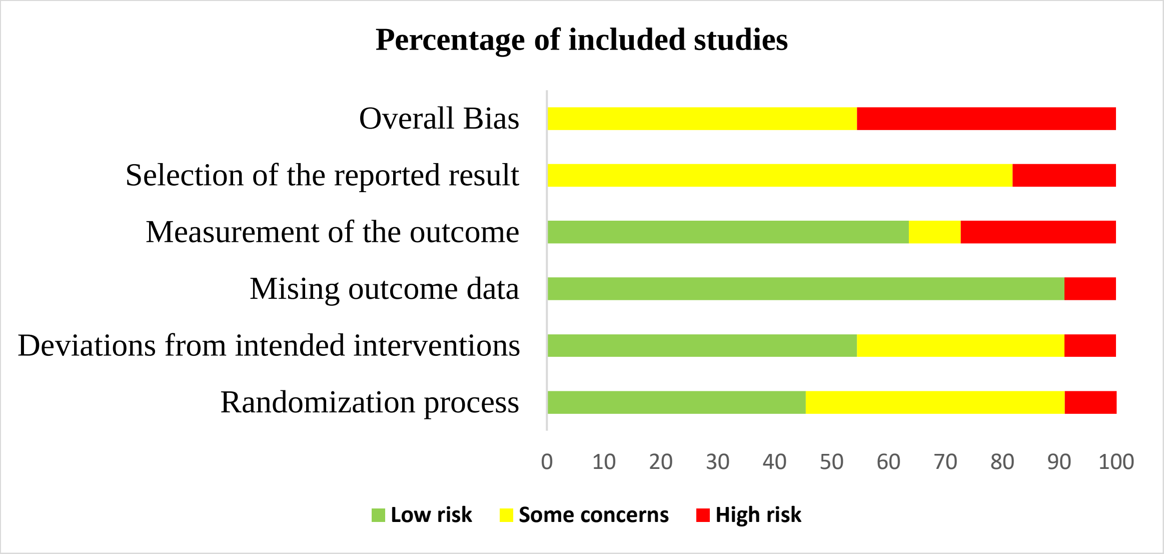


**Online supplemental figure 1.** Risk of bias assessment in each of the five domains and overall bias. Percentage of studies showing low risk of bias, some concerns and high risk of bias.

Note: For studies having more than one relevant outcome, each outcome is considered separately for risk of bias assessment.

For peer review only

**Online supplemental file 1.**

**Database-specific search strategies**

**AMED**

(Propriocep* OR (ZU "proprioception") OR Kinesthe* OR (ZU "kinesthesis") OR sensorimotor OR sensory-motor OR "joint position sense" OR "joint position detection" OR "threshold to detect passive motion" OR "passive motion direction discrimination" OR "passive motion detection threshold" OR "threshold for motion detection" OR "threshold hunting" OR "detection threshold" OR "discrimination threshold" OR "ipsilateral matching" OR "contralateral matching" OR "joint angle error" OR "distance estimation error" OR "passive recognition" OR "direction accuracy" OR "active reproduction" OR "joint reposition" OR "force sense" OR "force perception" OR "velocity sense" OR "active movement extent discrimination") AND (S1 AND S2 AND S3 AND S4)
S1: “Anterior Cruciate Ligament” OR (ZU "anterior cruciate ligament") OR “Knee joint” OR (ZU "knee joint)
S2: Injur* OR (ZU "injuries") OR (ZU "anterior cruciate ligament injuries") OR Reconstruction OR (ZU "anterior cruciate ligament reconstruction") OR
S3: Propriocep* OR (ZU "proprioception") OR Neuromuscular OR sensorimotor OR sensory-motor OR "Kinetic chain" OR (ZU "kinetics") OR Coordination OR Balance OR (ZU "balance") OR Plyometric (ZU "plyometric exercise") OR Vibration OR (ZU "vibration") OR Exercise* OR (ZU "exercise") OR Intervention OR Training OR Rehabilitation OR (ZU "rehabilitation") OR Therap* OR (ZU "therapy") OR Treatment
S4: Propriocep* OR (ZU "proprioception") OR Kinesthe* OR (ZU "kinesthesis") OR sensorimotor OR sensory-motor OR "joint position sense" OR "joint position detection" OR "threshold to detect passive motion" OR "passive motion direction discrimination" OR "passive motion detection threshold" OR "threshold for motion detection" OR "threshold hunting" OR "detection threshold" OR "discrimination threshold" OR "ipsilateral matching" OR "contralateral matching" OR "joint angle error" OR "distance estimation error" OR "passive recognition" OR "direction accuracy" OR "active reproduction" OR "Joint reposition" OR "force sense" OR "force perception" OR "velocity sense" OR "active movement extent discrimination"
Limiters - Language: English, Expanders - Apply related words, Search modes - Find any of my search terms, Interface - EBSCOhost Research Databases, Search Screen - Advanced Search, Database - AMED - The Allied and Complementary Medicine Database

**CINAHL**

Limiters - Peer Reviewed; Human; Language: English, Expanders - Apply related words, Search modes - Find any of my search terms, Interface - EBSCOhost Research Databases, Search Screen - Advanced Search, Database - CINAHL with Full Text
(Propriocep* OR (MH "Proprioception+") OR Kinesthe* OR (MH "Kinesthesis") OR sensorimotor OR sensory-motor OR "joint position sense" OR "joint position detection" OR "threshold to detect passive motion" OR "passive motion direction discrimination" OR "passive motion detection threshold" OR "threshold for motion detection" OR "threshold hunting" OR "detection threshold" OR "discrimination threshold" OR "ipsilateral matching" OR "contralateral matching" OR "joint angle error" OR "distance estimation error" OR "passive recognition" OR

For peer review only

"direction accuracy" OR "active reproduction" OR "Joint reposition" OR "Active movement extent discrimination" OR "force sense" OR "force perception" OR "velocity sense") AND (S6 AND S7 AND S8 AND S9)
S6: “Anterior Cruciate Ligament” OR (MH "Anterior Cruciate Ligament") “Knee joint” OR (MH "Knee Joint+"
S7: Injur* OR (MH "Anterior Cruciate Ligament Injuries") OR Reconstruction OR (MH "Anterior Cruciate Ligament Reconstruction") OR Rupture OR Tear OR (MH "Rupture+") OR Conservative OR Deficiency OR “Joint instability” OR (MH "Joint Instability+"
S8: Propriocep* OR (MH "Proprioception+") OR Neuromuscular OR (MH "Neuromuscular Control") OR sensorimotor OR "sensory-motor" OR "Kinetic chain" OR (MH "Closed Kinetic Chain Exercises") OR (MH "Open Kinetic Chain Exercises") OR Coordination OR Balance OR (MH "Balance Training, Physical") OR (MH "Balance, Postural") OR Plyometric OR Vibration OR (MH "Vibration" OR Exercise* OR (MH "Exercise+") OR Intervention OR Training OR Rehabilitation OR Therapy OR (MH "Physical Therapy+") OR Treatment
S9: Propriocep* OR (MH "Proprioception+") OR Kinesthe* OR (MH "Kinesthesis") OR sensorimotor OR sensory-motor OR "joint position sense" OR "joint position detection" OR "threshold to detect passive motion" OR "passive motion direction discrimination" OR "passive motion detection threshold" OR "threshold for motion detection" OR "threshold hunting" OR "detection threshold" OR "discrimination threshold" OR "ipsilateral matching" OR "contralateral matching" OR "joint angle error" OR "distance estimation error" OR "passive recognition" OR "direction accuracy" OR "active reproduction" OR "Joint reposition" OR "force sense" OR "force perception" OR "velocity sense" OR "Active movement extent discrimination"

**Physical Education Index (ProQuest)**

((("Anterior Cruciate Ligament" OR "Knee joint") AND (Injur* OR Trauma OR Reconstruct* OR Ruptur* OR Tear OR Conservative OR Deficienc* OR "Joint instabilit*") AND (Propriocep* OR Kinesthes* OR neuromuscular OR sensorimotor OR sensory-motor OR "Kinetic chain" OR Coordination OR Balance OR Plyometric OR Vibration OR Exercise* OR Intervention OR Training OR Rehabilitation OR Therap* OR Treatment) AND (Propriocep* OR Kinesthes* OR sensorimotor OR sensory-motor OR "joint position sense" OR "joint position detection" OR "threshold to detect passive motion" OR "passive motion direction discrimination" OR "passive motion detection threshold" OR "threshold for motion detection" OR "threshold hunting" OR "detection threshold" OR "discrimination threshold" OR "ipsilateral matching" OR "contralateral matching" OR "joint angle error" OR "distance estimation error" OR "passive recognition" OR "direction accuracy" OR "active reproduction" OR "Joint reposition" OR "active movement extent discrimination" OR "force sense" OR "force perception" OR "velocity sense"))) AND at.exact("Article") AND la.exact("ENG") AND PEER(yes)

**PubMed**

(((((Anterior Cruciate Ligament[Text Word] OR "Anterior Cruciate Ligament"[Mesh] OR Knee joint[Text Word] OR "knee joint"[MeSH Terms]) AND "loattrfull text"[sb]) AND (((injury[All Fields]) OR Reconstruction[Text Word] OR "Anterior Cruciate Ligament Reconstruction"[Mesh] OR "Anterior Cruciate Ligament Injuries"[Mesh] OR Rupture[Text Word] OR Tear[Text Word] OR "Rupture"[Mesh] OR Conservative[Text Word] OR

"Conservative Treatment"[Mesh] OR Deficiency[Text Word] OR Joint instability[Text Word] OR "Joint Instability"[Mesh]))) AND (((proprioception[All Fields]) OR "Proprioception"[Mesh] OR Neuromuscular[Text Word] OR sensorimotor[Text Word] OR sensory-motor[Text Word] OR Kinetic chain[Text Word] OR Coordination[Text Word] OR "Psychomotor Performance"[Mesh] OR Balance[Text Word] OR "Postural Balance"[Mesh] OR Plyometric[Text Word] OR "Plyometric Exercise"[Mesh] OR ("exercise"[MeSH Terms] OR "exercises"[All Fields] OR "exercise therapy"[MeSH Terms]) OR "Exercise Therapy"[Mesh] OR Intervention[Text Word] OR Training[Text Word] OR "Resistance Training"[Mesh] OR Rehabilitation[Text Word] OR "Rehabilitation"[Mesh] OR Therapy[Text Word] OR Treatment[Text Word] OR "Treatment Outcome"[Mesh]))) AND (((proprioception[All Fields]) OR "Proprioception"[Mesh] OR ("kinesthesis"[MeSH Terms] OR "kinesthesis"[All Fields]) OR "Kinesthesis"[Mesh] OR joint position sense[Text Word] OR (("joints"[MeSH Terms] OR "joints"[All Fields] OR "joint"[All Fields]) AND position detection[Text Word]) OR threshold to detect passive motion[Text Word] OR (passive[All Fields] AND motion direction discrimination[Text Word]) OR (passive[All Fields] AND motion detection threshold[Text Word]) OR (threshold[All Fields] AND motion detection[Text Word]) OR threshold hunting[Text Word] OR detection threshold[Text Word] OR discrimination threshold[Text Word] OR (ipsilateral[All Fields] AND matching[Text Word]) OR contralateral matching[Text Word] OR joint angle error[Text Word] OR distance estimation error[Text Word] OR passive recognition[Text Word] OR direction accuracy[Text Word] OR active reproduction[Text Word] OR Joint reposition[Text Word] OR force sense[Text Word] OR force perception[Text Word] OR velocity sense[Text Word] OR (active[All Fields] AND ("movement"[MeSH Terms] OR "movement"[All Fields]) AND extent[All Fields] AND ("discrimination (psychology)"[MeSH Terms] OR ("discrimination"[All Fields] AND "(psychology)"[All Fields]) OR "discrimination (psychology)"[All Fields] OR "discrimination"[All Fields])) OR sensorimotor[Text Word] OR sensory-motor[Text Word]) AND "loattrfull text"[sb])) AND "loattrfull text"[sb] AND ("loattrfull text"[sb] AND English[lang]) AND English[lang]

**Scopus**

( "Anterior Cruciate Ligament" OR "Knee joint" ) AND ( injur* OR trauma OR reconstruct* OR ruptur* OR tear OR conservative OR deficienc* OR "Joint instabilit*" ) AND ( propriocep* OR kinesthes* OR neuromuscular OR sensorimotor OR sensory-motor OR "Kinetic chain" OR coordination OR balance OR plyometric OR vibration OR exercise* OR intervention OR training OR rehabilitation OR therap* OR treatment ) AND ( propriocep* OR kinesthes* OR "joint position sense" OR "joint position detection" OR "threshold to detect passive motion" OR "passive motion direction discrimination" OR "passive motion detection threshold" OR "threshold for motion detection" OR "threshold hunting" OR "detection threshold" OR "discrimination threshold" OR "ipsilateral matching" OR "contralateral matching" OR "joint angle error" OR "distance estimation error" OR "passive recognition" OR "direction accuracy" OR "active reproduction" OR "Joint reposition" OR "active movement extent discrimination" OR "force sense" OR "force perception" OR "velocity sense" OR sensorimotor OR sensory-motor ) AND NOT INDEX (medline) AND (LIMIT-TO ( SRCTYPE , "j")) AND (LIMIT-TO ( DOCTYPE , "ar")) AND (LIMIT-TO ( SUBJAREA , "MEDI") OR LIMIT-TO ( SUBJAREA , "HEAL" ) OR LIMIT-TO ( SUBJAREA , "NEUR")) AND (LIMIT-TO ( LANGUAGE , "English")) AND ( LIMIT-TO (EXACTKEYWORD , "Human") OR LIMIT-TO (EXACTKEYWORD ,

For peer review only

"Article") OR LIMIT-TO (EXACTKEYWORD , "Male" ) OR LIMIT-TO ( EXACTKEYWORD , "Female") OR LIMIT-TO (EXACTKEYWORD , "Controlled Study") OR LIMIT-TO (EXACTKEYWORD , "Proprioception"))

**SPORTDiscus**

Limiters - Peer Reviewed; Language: English; Publication Type: Academic Journal; Document Type: Article, Expanders - Apply related words, Search modes - Find any of my search terms, Interface - EBSCOhost Research Databases, Search Screen - Advanced Search, Database - SPORTDiscus
(Propriocep* OR (DE "PROPRIOCEPTION") OR Kinesthe* OR sensorimotor OR sensory-motor OR "joint position sense" OR "joint position detection" OR "threshold to detect passive motion" OR "passive motion direction discrimination" OR "passive motion detection threshold" OR "threshold for motion detection" OR "threshold hunting" OR "detection threshold" OR "discrimination threshold" OR "ipsilateral matching" OR "contralateral matching" OR "joint angle error" OR "distance estimation error" OR "passive recognition" OR "direction accuracy" OR "active reproduction" OR "Joint reposition" OR "force sense" OR "force perception" OR "velocity sense" OR "active movement extent discrimination") AND (S1 AND S2 AND S3 AND S4)
S1: Anterior Cruciate Ligament OR (DE "CRUCIATE ligaments") OR (DE "ANTERIOR cruciate ligament") “Knee joint” OR (DE "KNEE"
S2: Injur* OR (DE "ANTERIOR cruciate ligament injuries") OR (DE "CRUCIATE ligament injuries) OR Reconstruction OR Rupture OR Tear OR Conservative OR Deficiency OR “Joint instabilit*”
S3: Propriocep* OR (DE "PROPRIOCEPTION") OR Neuromuscular OR sensorimotor OR sensory-motor OR Kinetic chain OR Coordination OR (DE "MOTOR ability") OR Balance OR Plyometric OR (DE "PLYOMETRICS) OR Vibration OR Exercise* OR Intervention OR Training OR Rehabilitation OR (DE "TREATMENT programs") OR (DE "REHABILITATION") OR Therap* OR Treatment OR (DE "KNEE injuries -- Treatment")
S4: Propriocep* OR (DE "PROPRIOCEPTION") OR Kinesthe* OR sensorimotor OR sensory-motor OR "joint position sense" OR "joint position detection" OR "threshold to detect passive motion" OR "passive motion direction discrimination" OR "passive motion detection threshold" OR "threshold for motion detection" OR "threshold hunting" OR "detection threshold" OR "discrimination threshold" OR "ipsilateral matching" OR "contralateral matching" OR "joint angle error" OR "distance estimation error" OR "passive recognition" OR "direction accuracy" OR "active reproduction" OR "Joint reposition" OR "force sense" OR "force perception" OR "velocity sense" OR "active movement extent discrimination"

For peer review only

**Online supplemental file 2.**

**Screening protocol – to screen eligible studies at the title, abstract, and full-text screening stages**

**Questions for all stages: title, abstract and full-text screening (follow stages 1-9):**

1) Is the study published in a scientific journal or published as a dissertation/thesis?
   a. No - exclude
   b. Yes or uncertain - go to step 2
2) Is the study written in English?
   a. No - exclude
   b. Yes or uncertain - go to step 3
3) Does the study deal with individuals who are 15 years of age and above?
   a. No - exclude
   b. Yes or uncertain - go to step 4
4) Does this study investigate individuals with an anterior cruciate ligament injury managed with conservative treatment or surgical reconstruction?
   a. No - exclude
   b. Yes or uncertain - go to step 5
5) Is the study a primary study (i.e. no letter to the editor, book reviews, published study designs/trial protocols, commentaries, editorials, interviews, newspaper articles, patient education handouts, consensus statements or clinical practice guidelines)?
   a. No - exclude
   b. Yes or uncertain - go to step 6
6) Does the intervention group in the study undergo neuromuscular training rehabilitation?
   a. No - exclude
   b. Yes or uncertain - go to step 7
7) Is the comparator/control group in the study include any of the following: any other therapy, conventional training, usual care, placebo or sham therapy?
   a. No - exclude
   b. Yes or uncertain - go to step 8
8) Does the study evaluate knee proprioception using a specific test (joint position sense, joint position detection, threshold to detect passive motion, passive motion direction discrimination, passive motion detection threshold, threshold for motion detection, threshold hunting, detection threshold, discrimination threshold, ipsilateral matching, contralateral matching, joint angle error, distance estimation error, passive recognition, direction accuracy, active reproduction, active movement extent discrimination, force sense, force perception, velocity sense or any other related tests)- before and after the intervention?
   a. No - exclude
   b. Yes or uncertain - go to step 9
9) Does the study report (objective) focal measures of knee proprioception for any of the specific tests mentioned in point 8?
   a. No - exclude
   b. Yes or uncertain - choose one of the following options:

For peer review only - http://bmjopen.bmj.com/site/about/guidelines.xhtml

i. Title and abstract screening stage - include

ii. Full-text screening stage - follow step 10-11

**Additional questions for full-text stage only:**

10) Does the study use at least one (appropriate) statistical test to compare the intervention and comparator/control groups for knee proprioception?
    a. No - exclude
    b. Yes or uncertain - go to step 11
11) Are the points 1-10 scored as “yes or uncertain”
    a. If all “yes” - include
    b. If any “uncertain” - discuss with another reviewer to come to an agreement whether to include the study or not

For peer review only

**Online supplemental file 3.**
**Data extraction template**

| | |
|---|---|
| Publication details | Study citation, clinical trial registration, and published study protocol if available |
| Aim of the study | Primary and/or secondary aims relevant for the review. |
| Eligibility criteria | Inclusion and exclusion criteria for participants |
| Randomized controlled trial or controlled clinical trial | Randomization method? |
| Participant allocation | Concealed or not? |
| Number of participants identified | Identified, included and excluded? |
| All participants accounted for entire study | Yes or no? |
| Experimental group | Experimental intervention (type of neuromuscular rehabilitation training) given. |
| Comparator group | Comparator intervention given. |
| Assessment method, equipment used, and outcome measure(s) of interest | Those related to knee-specific proprioception senses. |
| Method(s) used for measuring the outcome(s) appropriate? | Authors quoted any data on reliability and validity based on the previous literature or their own data? |
| Multiple measurements of the same outcome measure within the outcome domain? | Different methods measuring same proprioception sense and different time points? |
| Participant characteristics | Anthropometric, demographic, physical activity and function levels, and any other relevant information to ACL injury and/or surgery. |
| Groups were similar at baseline | Anthropometrics, demographics, outcome measure(s) of interest, and any other prognostic indicators. |
| Blinding | Participants, investigators, therapists/clinicians/those delivering the interventions, and outcome assessors. |
| The outcome measure of interest was obtained from more than 85% of the participants initially allocated to groups | For continuous outcomes, availability of data from 95% (or possibly 90%) of the participants would often be sufficient. |
| If data were missing, how they were handled | 'Last observation carried forward', 'baseline observation carried forward' or any other method? |
| Analyses preplanned | Information available from Registered trial protocol or any other relevant information available? |
| Between-group statistical comparisons | Statistical analysis for measurement of proprioception was done by "intention to treat" or "per-protocol" analysis? Multiple analysis of data? Corrected for multiple analysis of data? Selective reporting of analysis? |
| Results | Selective reporting of a particular outcome measurement? |
| Conclusion | Authors' conclusions |

For peer review only

For peer review only

**The PRISMA for Abstracts Checklist**

| TITLE | CHECKLIST ITEM | REPORTED ON PAGE # |
|---|---|---|
| 1. Title: | Identify the report as a systematic review, meta-analysis, or both. | 2 |
| **BACKGROUND** | | |
| 2. Objectives: | The research question including components such as participants, interventions, comparators, and outcomes. | 2 |
| **METHODS** | | |
| 3. Eligibility criteria: | Study and report characteristics used as criteria for inclusion. | |
| 4. Information sources: | Key databases searched and search dates. | 2 |
| 5. Risk of bias: | Methods of assessing risk of bias. | |
| **RESULTS** | | |
| 6. Included studies: | Number and type of included studies and participants and relevant characteristics of studies. | |
| 7. Synthesis of results: | Results for main outcomes (benefits and harms), preferably indicating the number of studies and participants for each. If meta-analysis was done, include summary measures and confidence intervals. | 2 |
| 8. Description of the effect: | Direction of the effect (i.e. which group is favoured) and size of the effect in terms meaningful to clinicians and patients. | |
| **DISCUSSION** | | |
| 9. Strengths and Limitations of evidence: | Brief summary of strengths and limitations of evidence (e.g. inconsistency, imprecision, indirectness, or risk of bias, other supporting or conflicting evidence) | 3 |
| 10. Interpretation: | General interpretation of the results and important implications | |
| **OTHER** | | |
| 11. Funding: | Primary source of funding for the review. | 1 |
| 12. Registration: | Registration number and registry name. | 3 |

# PRISMA 2009 Checklist

| Section/topic | # | Checklist item | Reported on page # |
|---|---|---|---|
| **TITLE** | | | |
| Title | 1 | Identify the report as a systematic review, meta-analysis, or both. | Page 1 |
| **ABSTRACT** | | | |
| Structured summary | 2 | Provide a structured summary including, as applicable: background; objectives; data sources; study eligibility criteria, participants, and interventions; study appraisal and synthesis methods; results; limitations; conclusions and implications of key findings; systematic review registration number. | Pages 2-3 |
| **INTRODUCTION** | | | |
| Rationale | 3 | Describe the rationale for the review in the context of what is already known. | Pages 5-7 |
| Objectives | 4 | Provide an explicit statement of questions being addressed with reference to participants, interventions, comparisons, outcomes, and study design (PICOS). | Page 7 |
| **METHODS** | | | |
| Protocol and registration | 5 | Indicate if a review protocol exists, if and where it can be accessed (e.g., Web address), and, if available, provide registration information including registration number. | PROSPERO registration number CRD42018107349 |
| Eligibility criteria | 6 | Specify study characteristics (e.g., PICOS, length of follow-up) and report characteristics (e.g., years considered, language, publication status) used as criteria for eligibility, giving rationale. | Pages 7-8 |
| Information sources | 7 | Describe all information sources (e.g., databases with dates of coverage, contact with study authors to identify additional studies) in the search and date last searched. | Page 8 |
| Search | 8 | Present full electronic search strategy for at least one database, including any limits used, such that it could be repeated. | Online supplemental file 1 |
| Study selection | 9 | State the process for selecting studies (i.e., screening, eligibility, included in systematic review, and, if applicable, included in the meta-analysis). | Pages 9-10, online supplemental file 2 |
| Data collection process | 10 | Describe method of data extraction from reports (e.g., piloted forms, independently, in duplicate) and any processes for obtaining and confirming data from investigators. | Page 9, Online supplemental file 3. Data extraction template |

For peer review only

# PRISMA 2009 Checklist

| | | | |
|---|---|---|---|
| Data items | 11 | List and define all variables for which data were sought (e.g., PICOS, funding sources) and any assumptions and simplifications made. | Online supplemental file 3. Data extraction template |
| Risk of bias in individual studies | 12 | Describe methods used for assessing risk of bias of individual studies (including specification of whether this was done at the study or outcome level), and how this information is to be used in any data synthesis. | Pages 9-10 |
| Summary measures | 13 | State the principal summary measures (e.g., risk ratio, difference in means). | Pages 9-11 |
| Synthesis of results | 14 | Describe the methods of handling data and combining results of studies, if done, including measures of consistency (e.g., $I^2$) for each meta-analysis. | Pages 9-11 |



| Section/topic | # | Checklist item | Reported on page # |
|---|---|---|---|
| Risk of bias across studies | 15 | Specify any assessment of risk of bias that may affect the cumulative evidence (e.g., publication bias, selective reporting within studies). | Pages 9-10 |
| Additional analyses | 16 | Describe methods of additional analyses (e.g., sensitivity or subgroup analyses, meta-regression), if done, indicating which were pre-specified. | NA |
| **RESULTS** | | | |
| Study selection | 17 | Give numbers of studies screened, assessed for eligibility, and included in the review, with reasons for exclusions at each stage, ideally with a flow diagram. | Figure 1 |
| Study characteristics | 18 | For each study, present characteristics for which data were extracted (e.g., study size, PICOS, follow-up period) and provide the citations. | Pages 11-12, Table 1 |
| Risk of bias within studies | 19 | Present data on risk of bias of each study and, if available, any outcome level assessment (see item 12). | Pages 12, Table 2 |
| Results of individual studies | 20 | For all outcomes considered (benefits or harms), present, for each study: (a) simple summary data for each intervention group (b) effect estimates and confidence intervals, ideally with a forest plot. | Table 1 |
| Synthesis of results | 21 | Present results of each meta-analysis done, including confidence intervals and measures of consistency. | NA |
| Risk of bias across studies | 22 | Present results of any assessment of risk of bias across studies (see Item 15). | Pages 11-12, Table |

For peer review only - http://bmjopen.bmj.com/site/about/guidelines.xhtml

# PRISMA 2009 Checklist

| | | | |
|---|---|---|---|
| | | | 2, Table 3, Table 4 |
| Additional analysis | 23 | Give results of additional analyses, if done (e.g., sensitivity or subgroup analyses, meta-regression [see Item 16]). | NA |
| **DISCUSSION** | | | |
| Summary of evidence | 24 | Summarize the main findings including the strength of evidence for each main outcome; consider their relevance to key groups (e.g., healthcare providers, users, and policy makers). | Pages 15-16 |
| Limitations | 25 | Discuss limitations at study and outcome level (e.g., risk of bias), and at review-level (e.g., incomplete retrieval of identified research, reporting bias). | Page 17-22 |
| Conclusions | 26 | Provide a general interpretation of the results in the context of other evidence, and implications for future research. | Page 22 |
| **FUNDING** | | | |
| Funding | 27 | Describe sources of funding for the systematic review and other support (e.g., supply of data); role of funders for the systematic review. | Page 23 |

*From:* Moher D, Liberati A, Tetzlaff J, Altman DG, The PRISMA Group (2009). Preferred Reporting Items for Systematic Reviews and Meta-Analyses: The PRISMA Statement. PLoS Med 6(7): e1000097. doi:10.1371/journal.pmed1000097

For more information, visit: **www.prisma-statement.org**.



For peer review only

# Synthesis Without Meta-analysis (SWiM) reporting items

The citation for the Synthesis Without Meta-analysis explanation and elaboration article is: Campbell M, McKenzie JE, Sowden A, Katikireddi SV, Brennan SE, Ellis S, Hartmann-Boyce J, Ryan R, Shepperd S, Thomas J, Welch V, Thomson H. Synthesis without meta-analysis (SWiM) in systematic reviews: reporting guideline BMJ 2020;368:l6890 http://dx.doi.org/10.1136/bmj.l6890

| **SWiM is intended to complement and be used as an extension to PRISMA** | | | |
|---|---|---|---|
| **SWiM reporting item** | **Item description** | **Page in manuscript where item is reported** | **Other*** |
| *Methods* | | | |
| **1** Grouping studies for synthesis | 1a) Provide a description of, and rationale for, the groups used in the synthesis (e.g., groupings of populations, interventions, outcomes, study design) | Lines 95-117, 123-143; further groupings of populations, interventions, outcomes, study design are explained in pages 12-15. | NA |
| | 1b) Detail and provide rationale for any changes made subsequent to the protocol in the groups used in the synthesis | NA | NA |
| **2** Describe the standardised metric and transformation methods used | Describe the standardised metric for each outcome. Explain why the metric(s) was chosen, and describe any methods used to transform the intervention effects, as reported in the study, to the standardised metric, citing any methodological guidance consulted | Line 200-202, 698-704; Table 1 | NA |
| **3** Describe the synthesis methods | Describe and justify the methods used to synthesise the effects for each outcome when it was not possible to undertake a meta-analysis of effect estimates | Lines 200-202 | NA |
| **4** Criteria used to prioritise results for | Where applicable, provide the criteria used, with supporting justification, to select the particular studies, or a particular study, for the main synthesis or to draw conclusions from the synthesis (e.g., based on study design, risk of bias assessments, directness in relation to the review question) | Lines 123-143, 176-187 | NA |



For peer review only - http://bmjopen.bmj.com/site/about/guidelines.xhtml

## Synthesis Without Meta-analysis (SWiM) reporting items

| | | | |
|---|---|---|---|
| summary and synthesis | | | |
| **SWiM reporting item** | **Item description** | **Page in manuscript where item is reported** | **Other*** |
| **5** Investigation of heterogeneity in reported effects | State the method(s) used to examine heterogeneity in reported effects when it was not possible to undertake a meta-analysis of effect estimates and its extensions to investigate heterogeneity | Heterogeneity was examined using GRADE domains (inconsistency and imprecision of findings and indirectness of evidence). Please see line 317–333. Tables 3 and 4. | NA |
| **6** Certainty of evidence | Describe the methods used to assess certainty of the synthesis findings | Lines 176-187 | NA |
| **7** Data presentation methods | Describe the graphical and tabular methods used to present the effects (e.g., tables, forest plots, harvest plots).<br><br>Specify key study characteristics (e.g., study design, risk of bias) used to order the studies, in the text and any tables or graphs, clearly referencing the studies included | Narrative summary on pages 11-16; Table 1 (study characteristics); Table 2 (risk of bias summary); Tables 3 and 4 (GRADE evidence synthesis); Figure 1 (screening of studies) | NA |
| *Results* | | | |

For peer review only

## Synthesis Without Meta-analysis (SWiM) reporting items

| | | | |
|---|---|---|---|
| **8** Reporting results | For each comparison and outcome, provide a description of the synthesised findings, and the certainty of the findings. Describe the result in language that is consistent with the question the synthesis addresses, and indicate which studies contribute to the synthesis | Lines 300-333; Tables 3 and 4 (GRADE evidence synthesis) | NA |
| *Discussion* | | | |
| **9** Limitations of the synthesis | Report the limitations of the synthesis methods used and/or the groupings used in the synthesis, and how these affect the conclusions that can be drawn in relation to the original review question | Lines 188-202 | NA |

PRISMA=Preferred Reporting Items for Systematic Reviews and Meta-Analyses.
*If the information is not provided in the systematic review, give details of where this information is available (e.g., protocol, other published papers (provide citation details), or website (provide the URL)).

For peer review only
